# Looking inside ourselves: a culture of kindness*

**DOI:** 10.5195/jmla.2018.478

**Published:** 2018-07-01

**Authors:** Julia Sollenberger

**Affiliations:** Director Emerita, Medical Center Libraries & Technologies, University of Rochester Medical Center; 601 Elmwood Avenue; Rochester, NY 14642

## Abstract

Looking inside ourselves—being present and attentive to our own and others’ words and feelings—helps us communicate and interact with a mindful, open heart. Mindfulness and patient-centeredness help caregivers provide higher quality care. Historical background on a predecessor of mindfulness—the biopsychosocial model of health and disease, developed at the University of Rochester—provides context for the mindfulness “movement” in health care. A culture of mindfulness, supported by mindfulness and meditation training for physicians and other health care providers, helps practitioners show greater compassion, kindness, and humanity, all qualities that patients need and deserve. In the health care world, many organizations have been created that focus on aspects of mindfulness. Some have a more clinical emphasis and others focus on behavioral or neuroscience research as it relates to meditation, mindfulness, compassion, and kindness. Mindfulness is also being taught in business schools and corporations. Leaders who approach their teams with respect, integrity, honesty, and kindness are more effective leaders. Organizations like Google, Nike, and Aetna, among others, use the concept of mindfulness, as well as emotional and social intelligence, to build interpersonal competencies and create more people-centered workplaces. As medical libraries live in the health care environment and medical library leaders are key to libraries’ present and future, there are strong reasons to address the concepts of mindfulness and kindness and put them to work in the medical library workplaces. A mindfulness meditation exercise closes the lecture, sending the attendees out into their day with calm and open minds and hearts.

Watch the video of the 2017 Janet Doe Lecture.

## SUPPLEMENTAL FILES

Appendix A Slides from the 2017 Janet Doe Lecture

Appendix B Transcript of the 2017 Janet Doe Lecture

