# Supplementary material for: Looking inside ourselves: a culture of kindness
Source: J Med Libr Assoc. 2018 Jul 1;106(3):283. doi: 10.5195/jmla.2018.478 (PMC6013138; doi:10.5195/jmla.2018.478)
Supplement: Appendix A [file jmla-106-283-s001.pdf]

Journal of the Medical Library Association
Supplemental content to J Med Libr Assoc. Jan;106(3): dx.doi.org/10.5195/jmla.2018.478
www.mlanet.org
© Sollenberger 2018

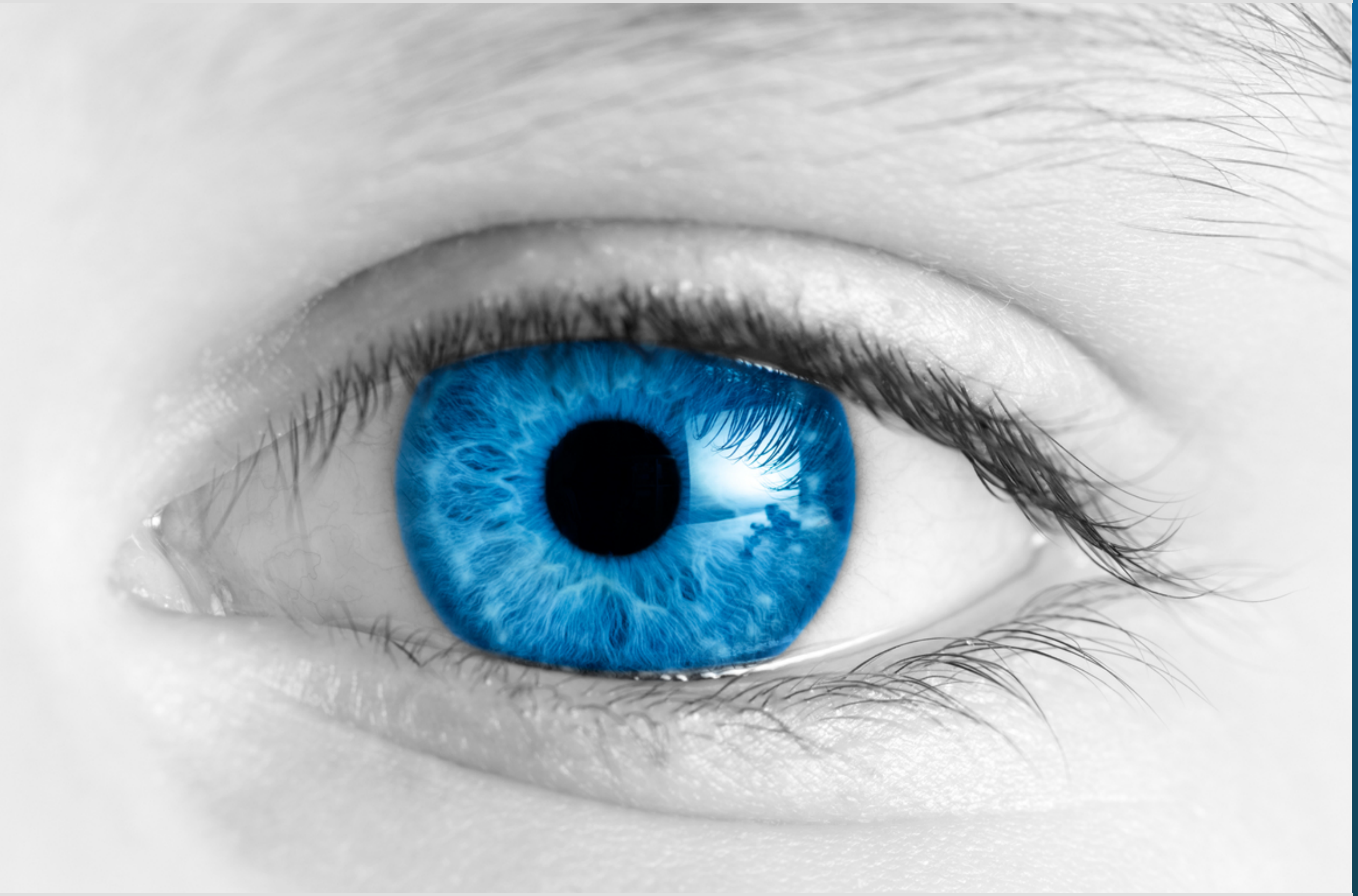

Looking Inside Ourselves: A Culture of Kindness

Julia Sollenberger
Director Emeritus
Edward G. Miner Library
University of Rochester

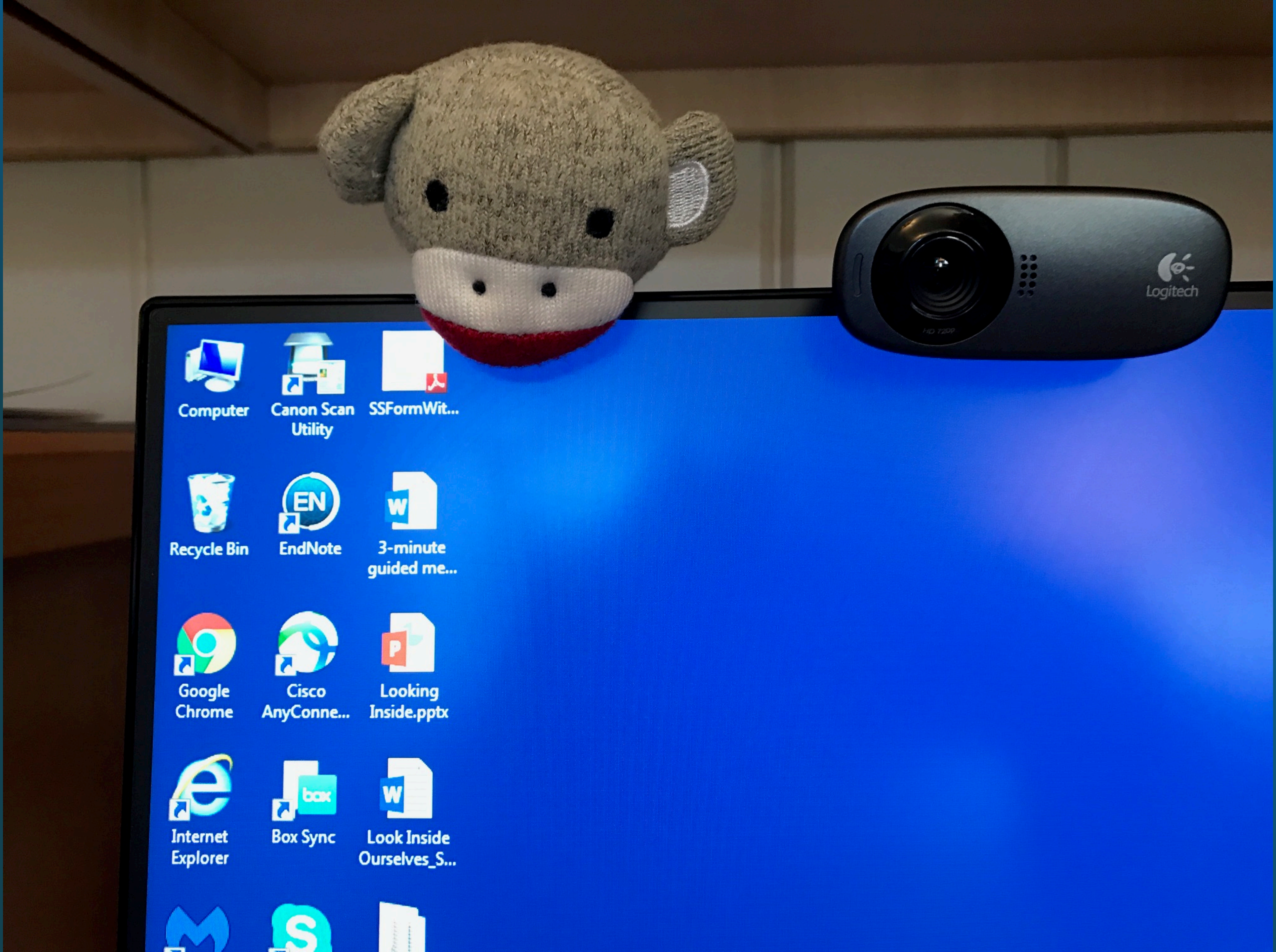

Computer
Canon Scan Utility
SSFormWit...
Recycle Bin
EndNote
3-minute guided me...
Google Chrome
Cisco AnyConne...
Looking Inside.pptx
Internet Explorer
Box Sync
Look Inside Ourselves_S...
Logitech

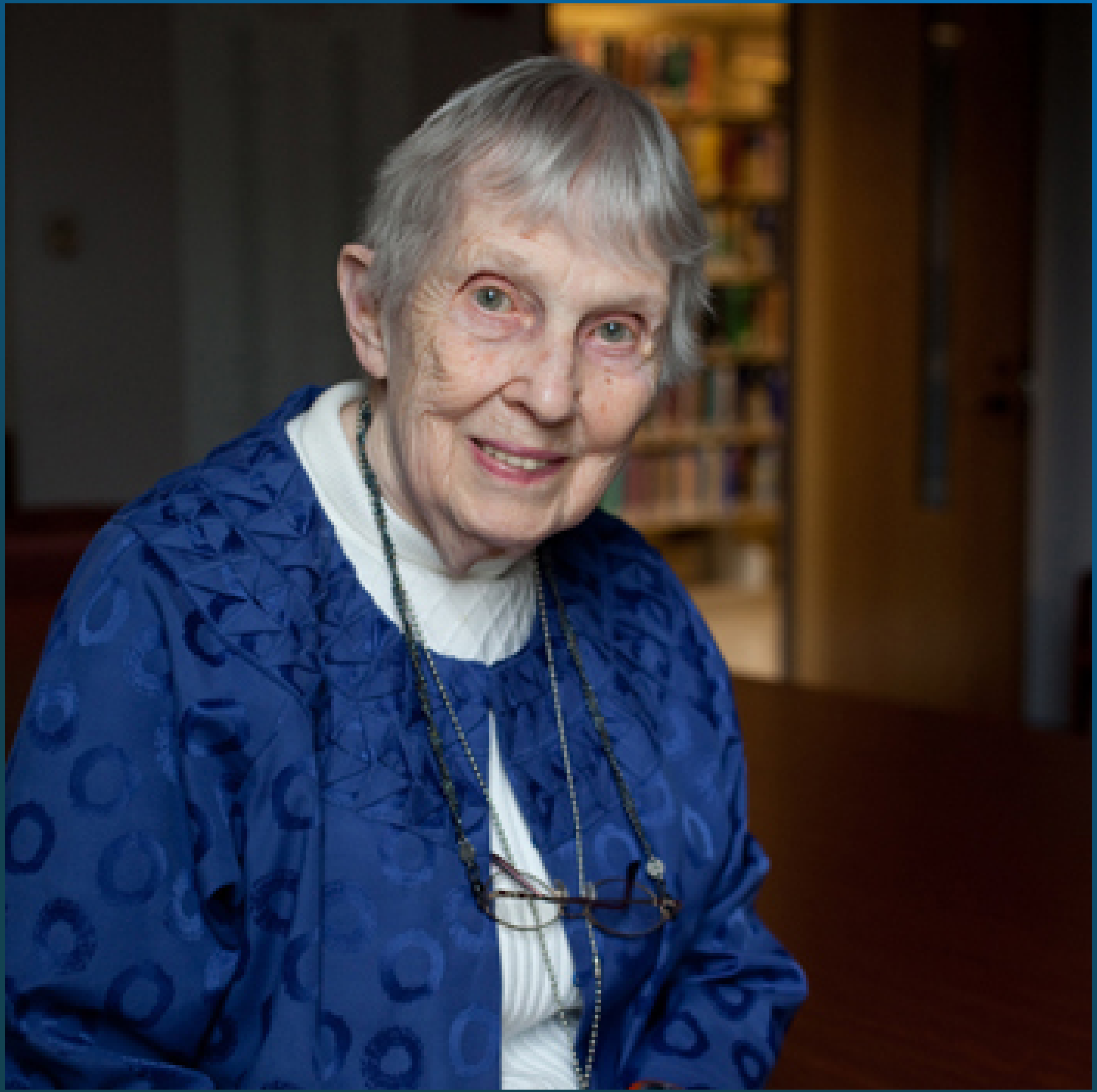

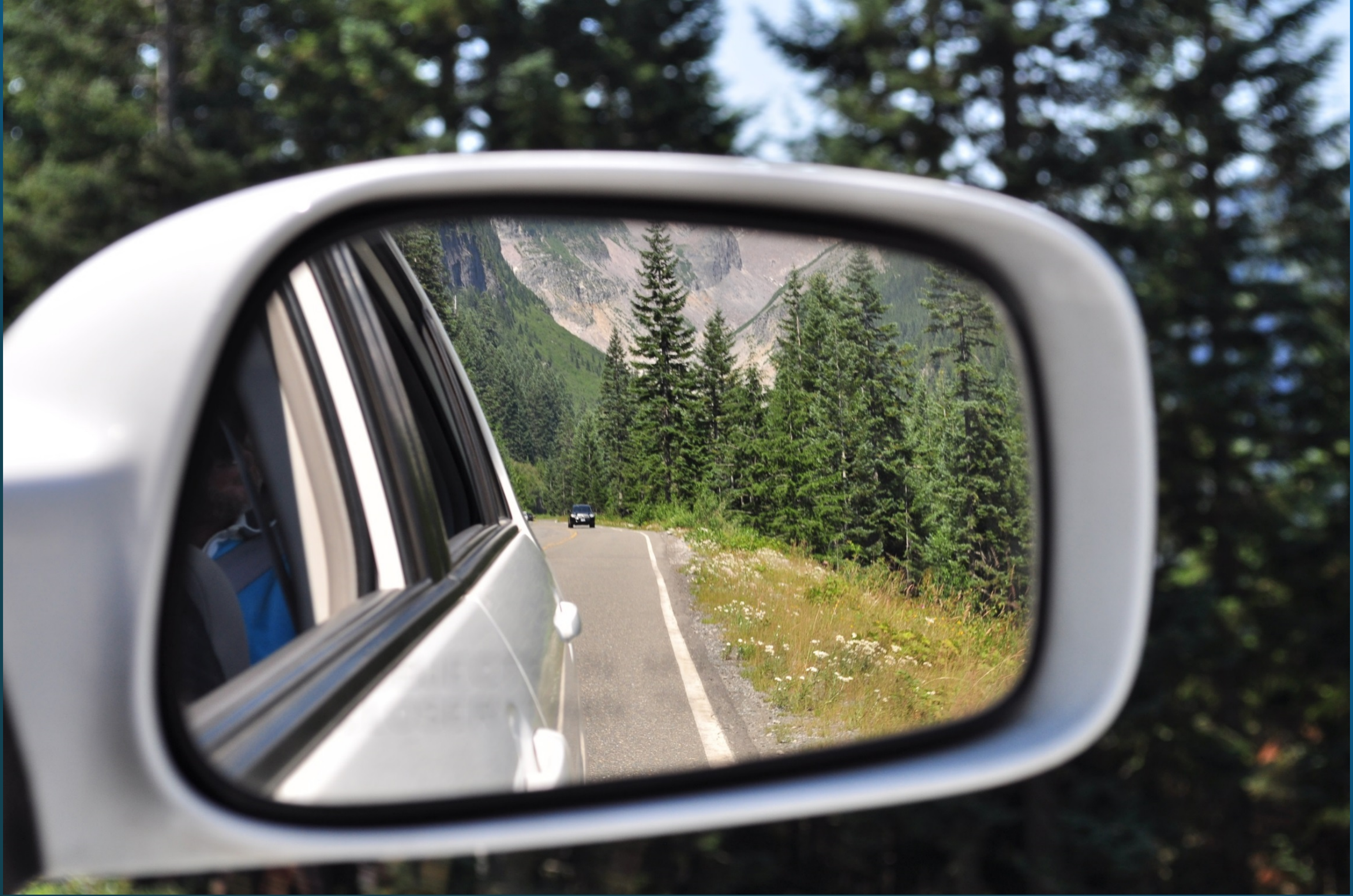

Photo by Joe Mabel, August 3, 2014

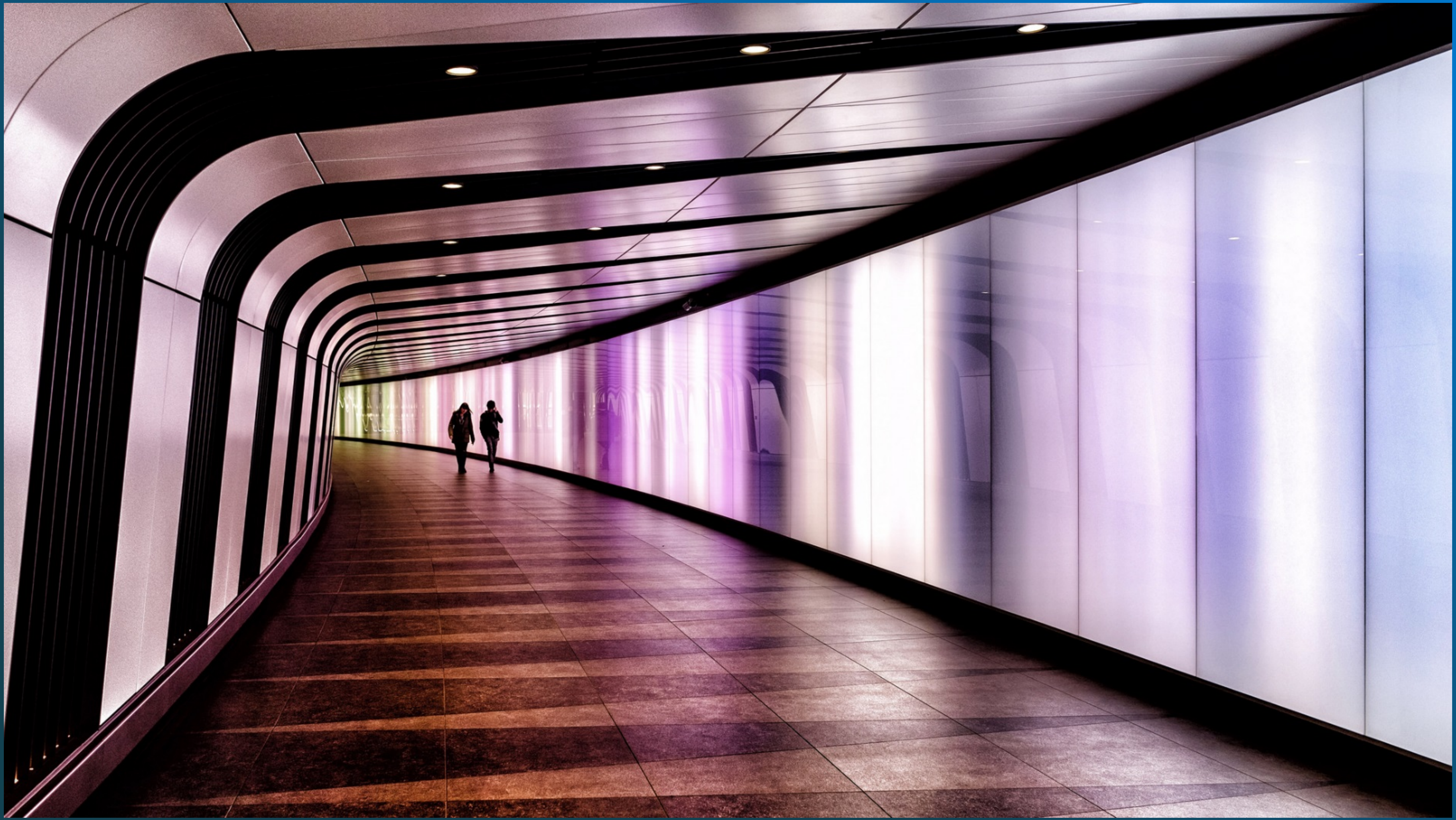

Photo by Dragan; November 23, 2015

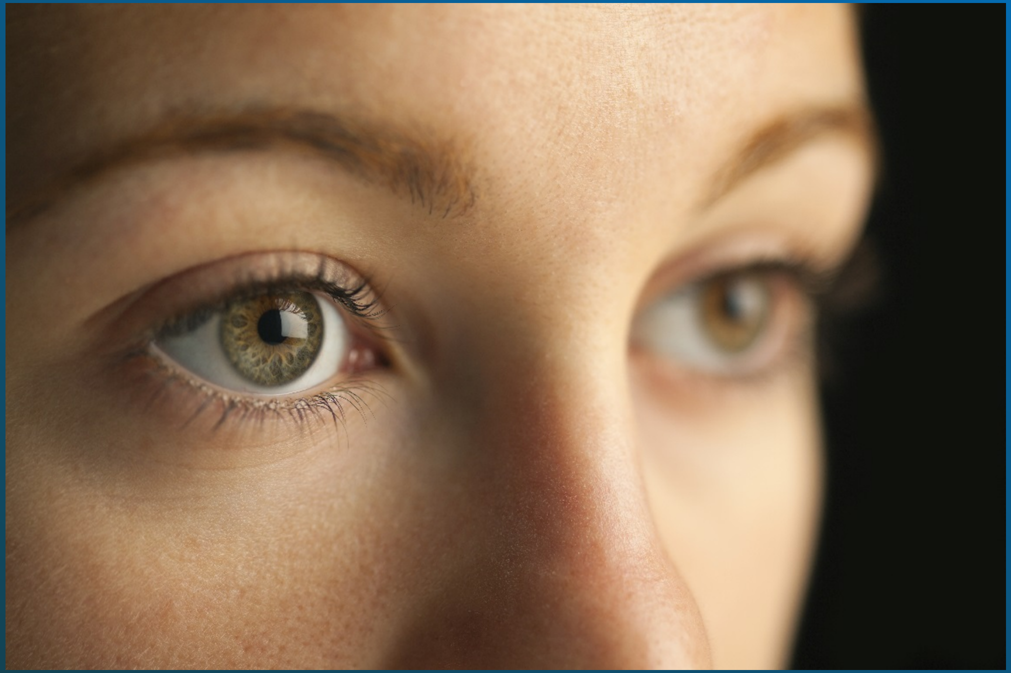

What lies behind us and what lies ahead of us are tiny matters to what lies within us.

-- Attributed to Ralph Waldo Emerson

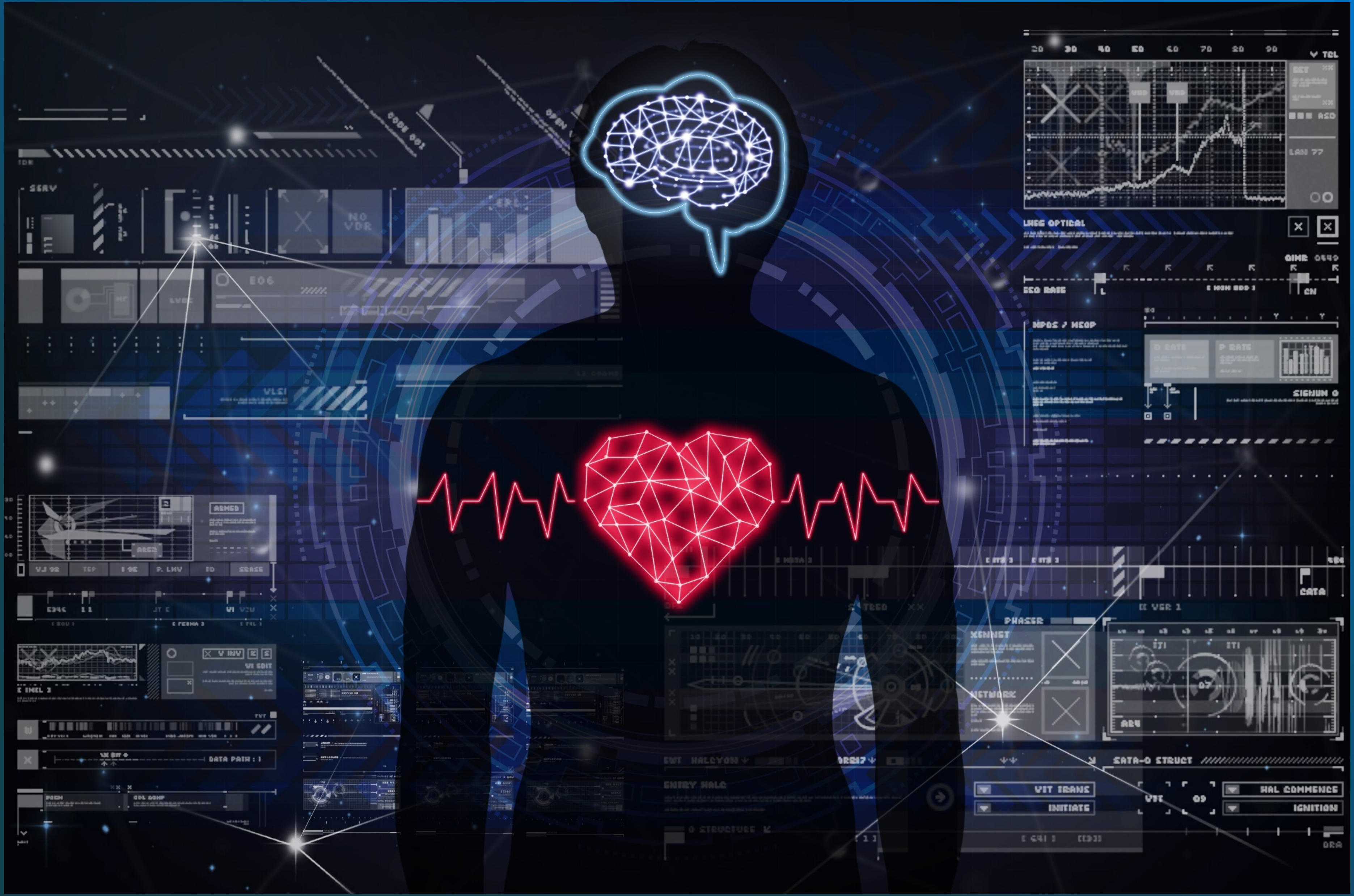

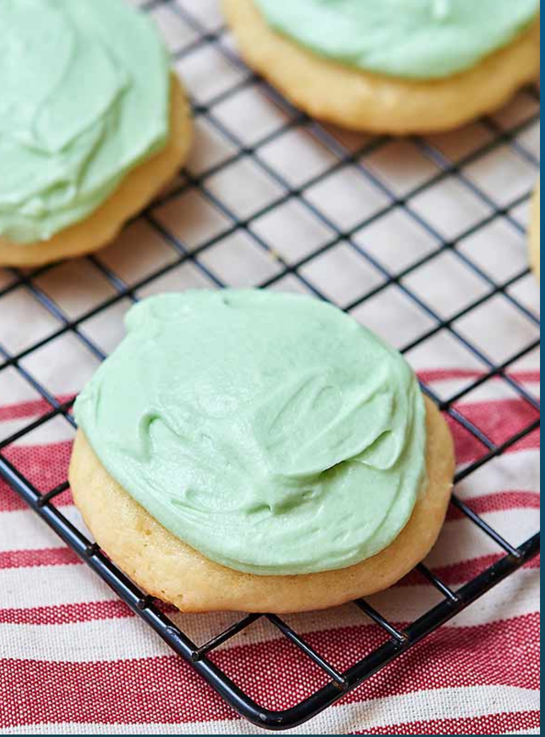
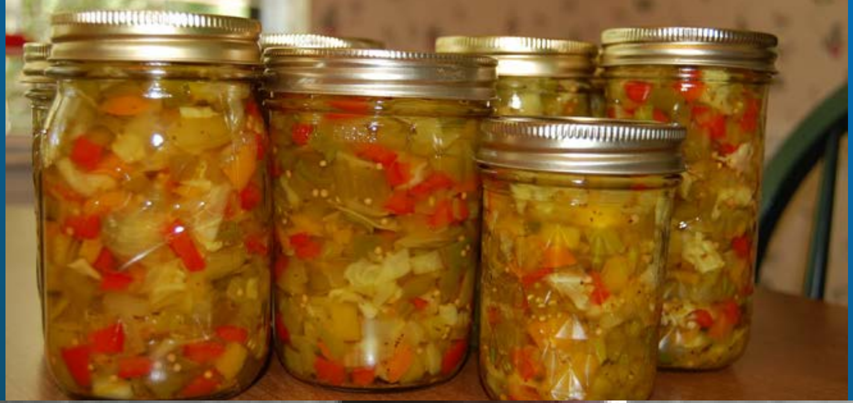
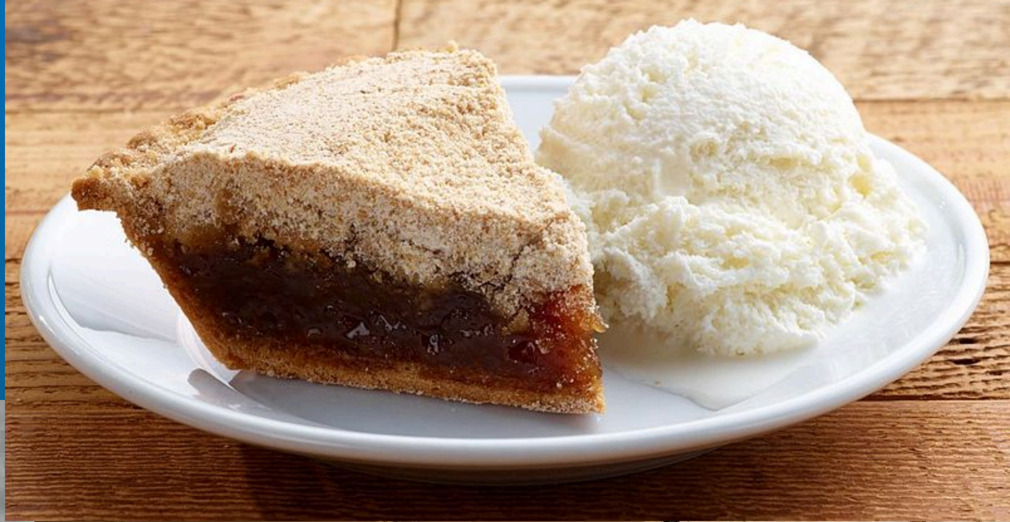
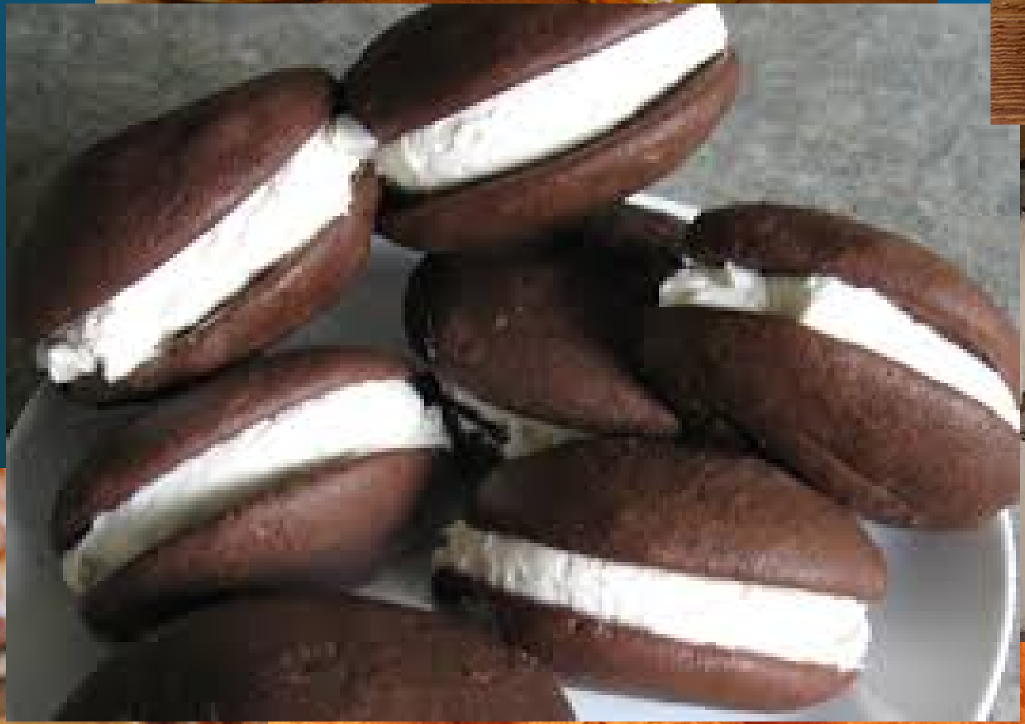
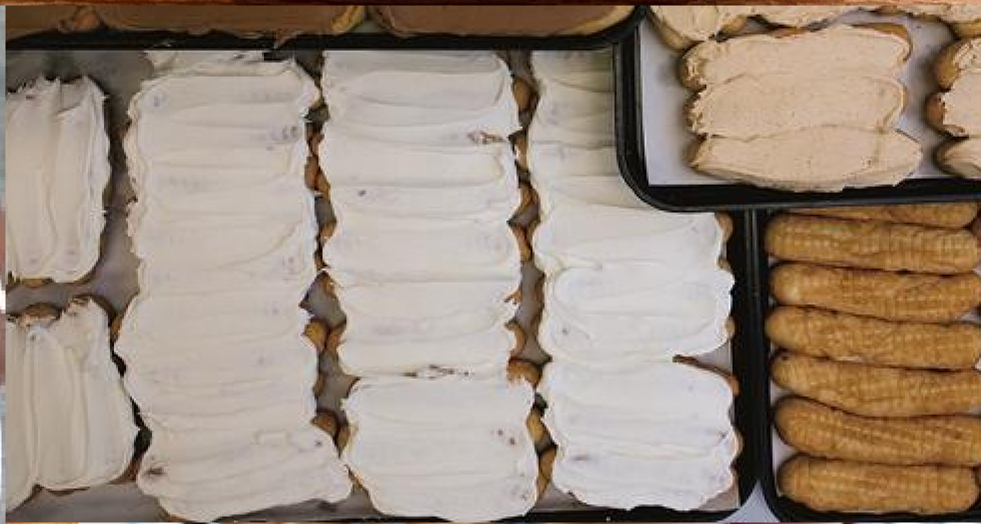
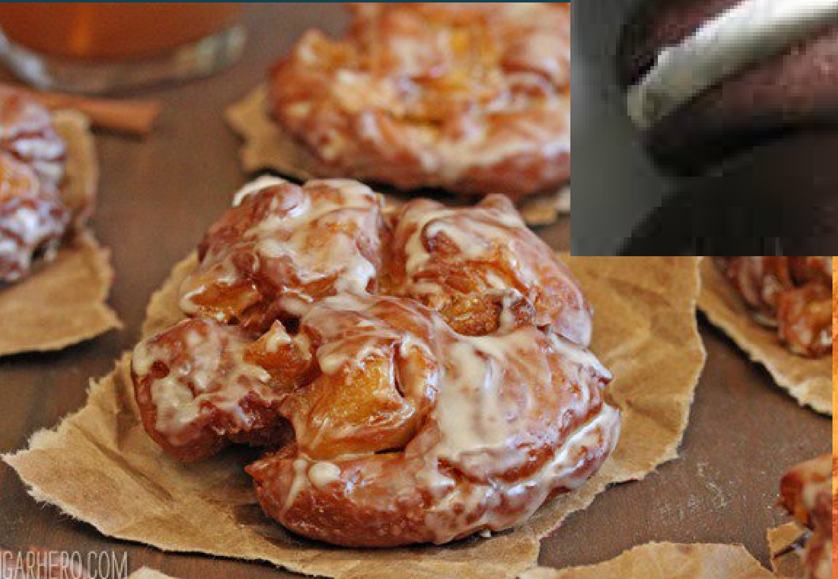
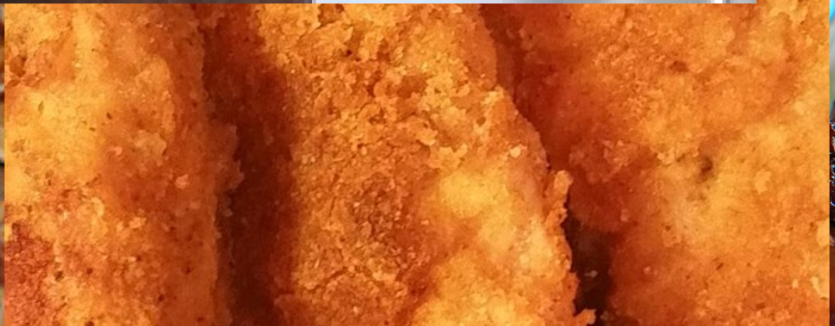
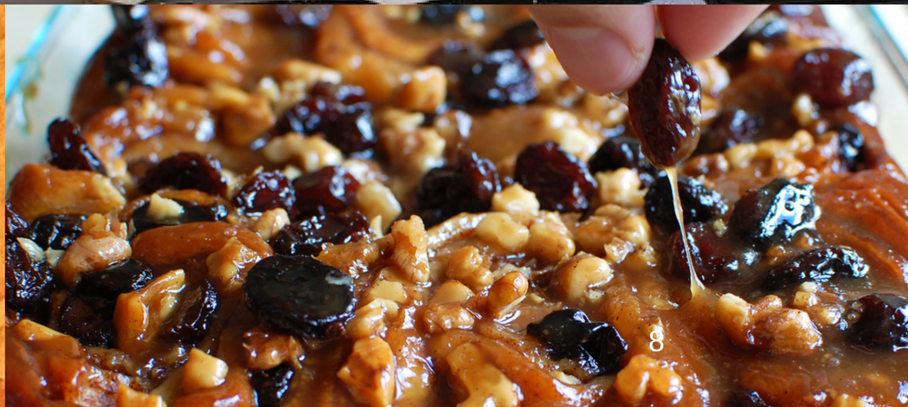

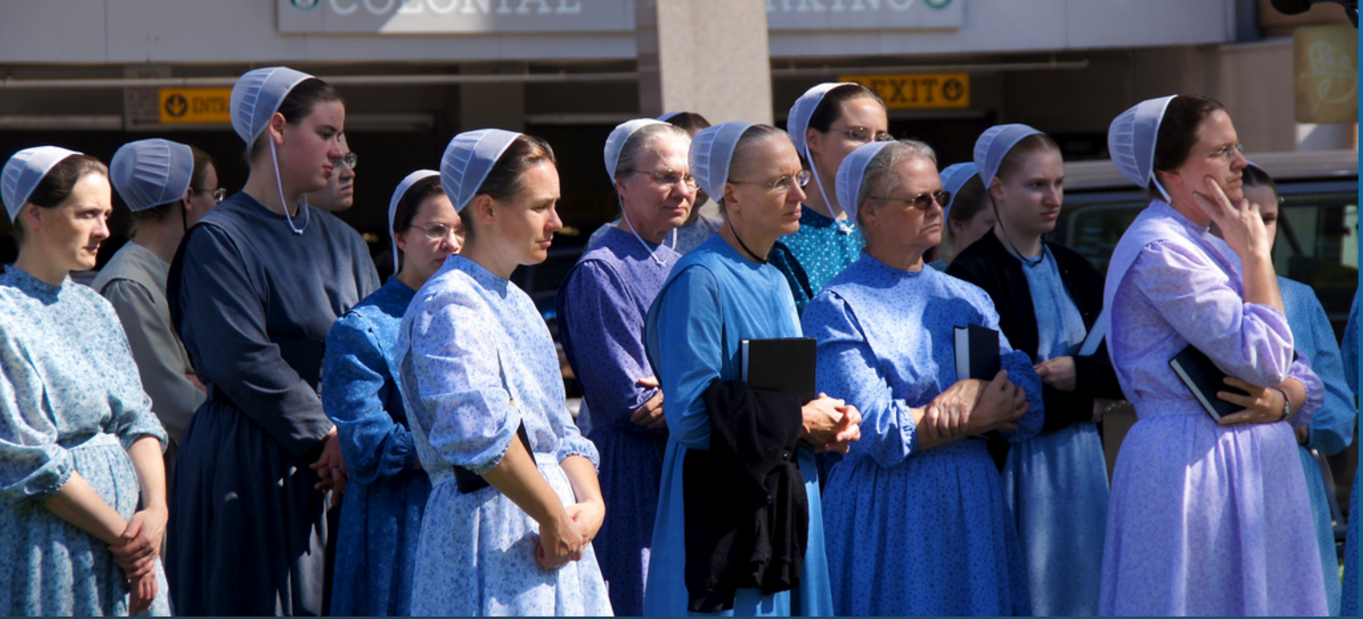
EXIT
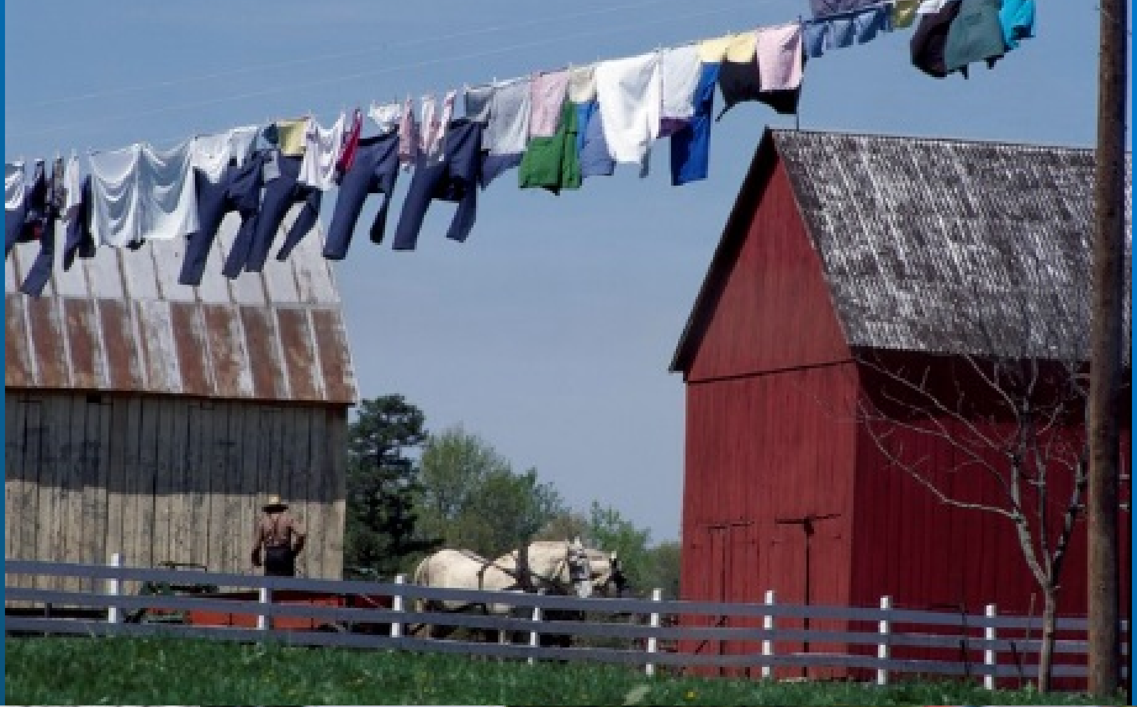
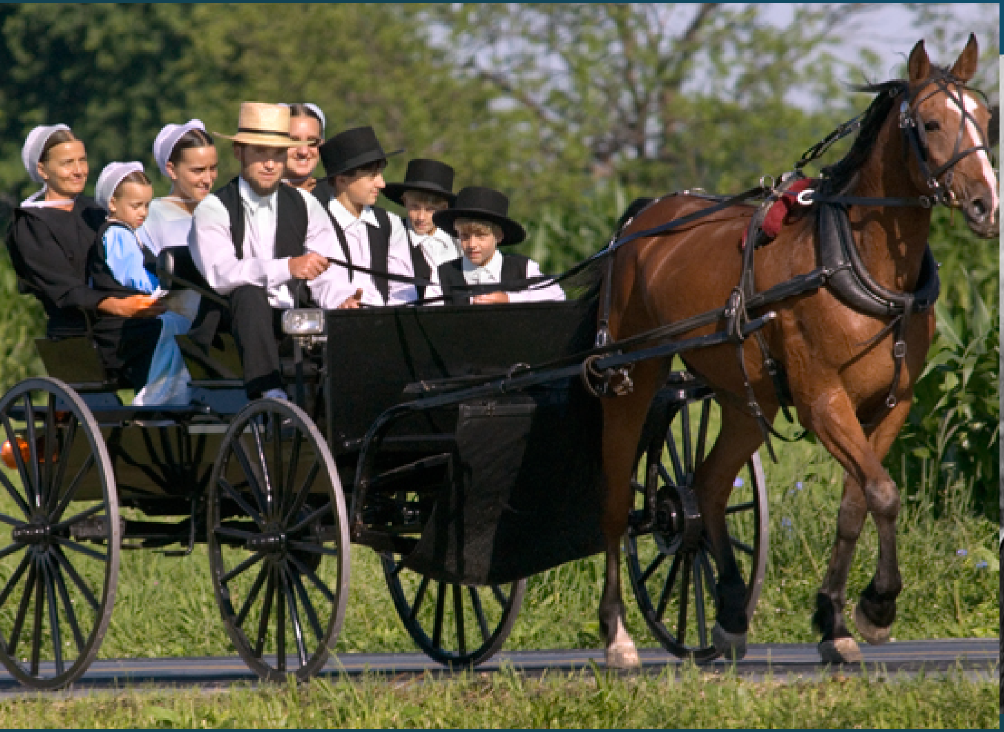
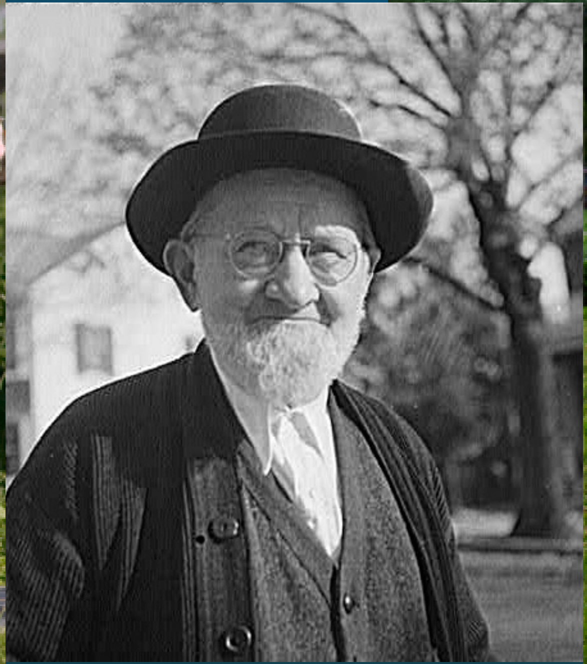
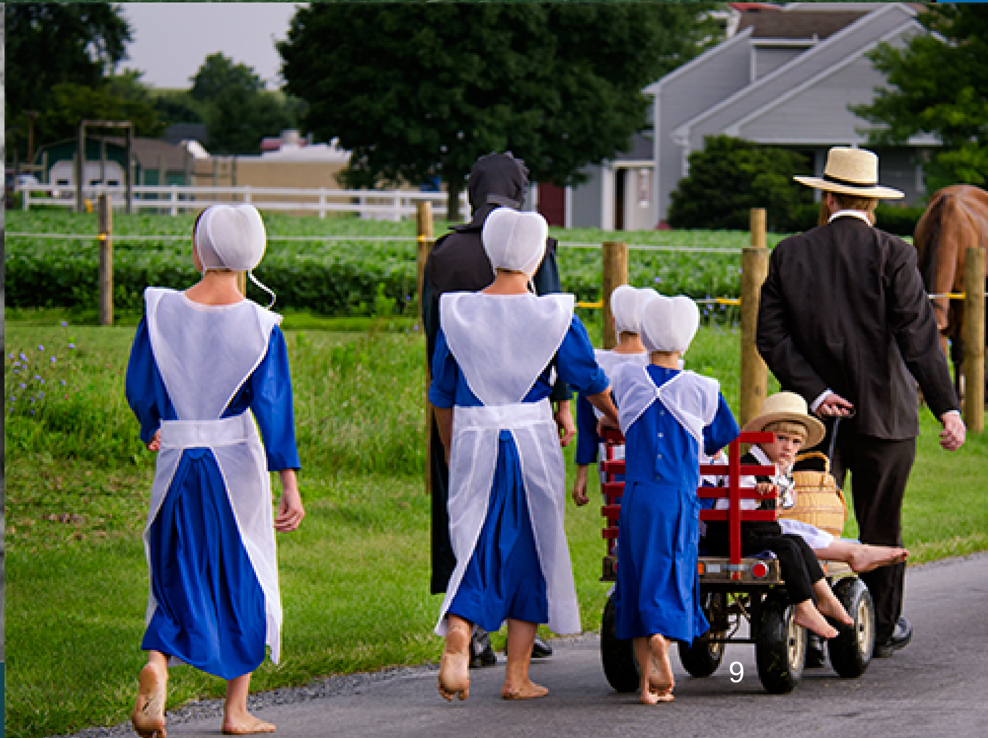

Why?

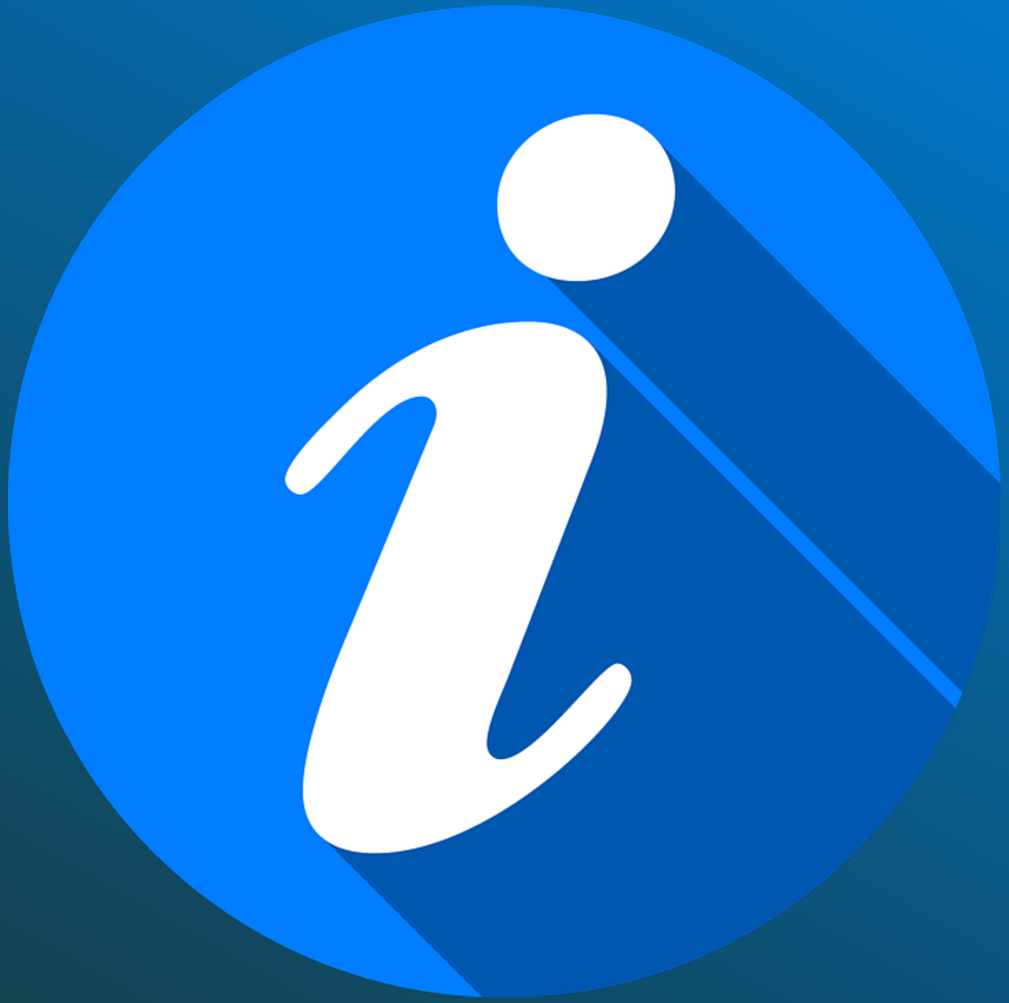

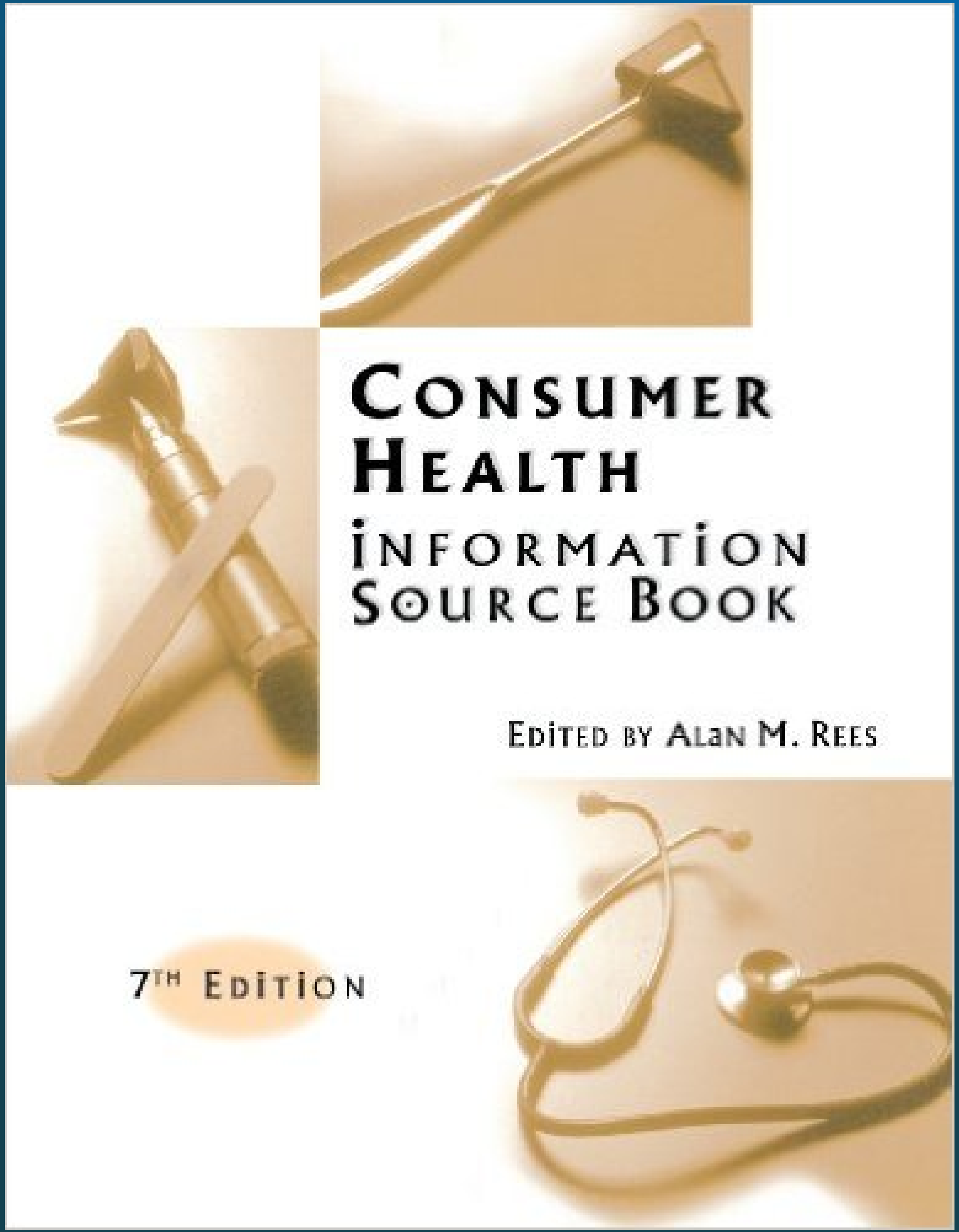

Alan Rees,
“Communication in the Physician-Patient Relationship,”

Janet Doe Lecture, 1992.

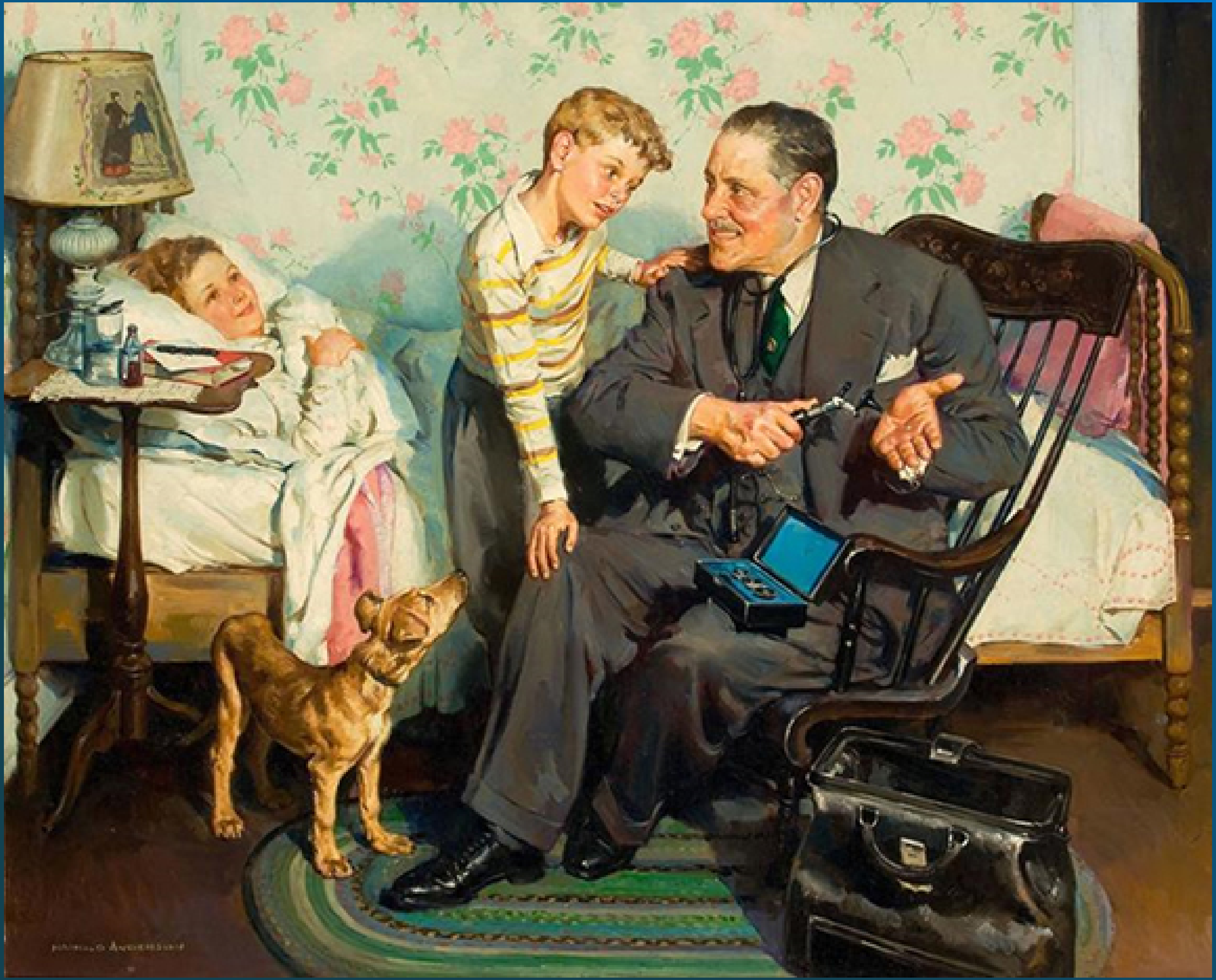

Illustration by Harold Anderson, 1894–1973

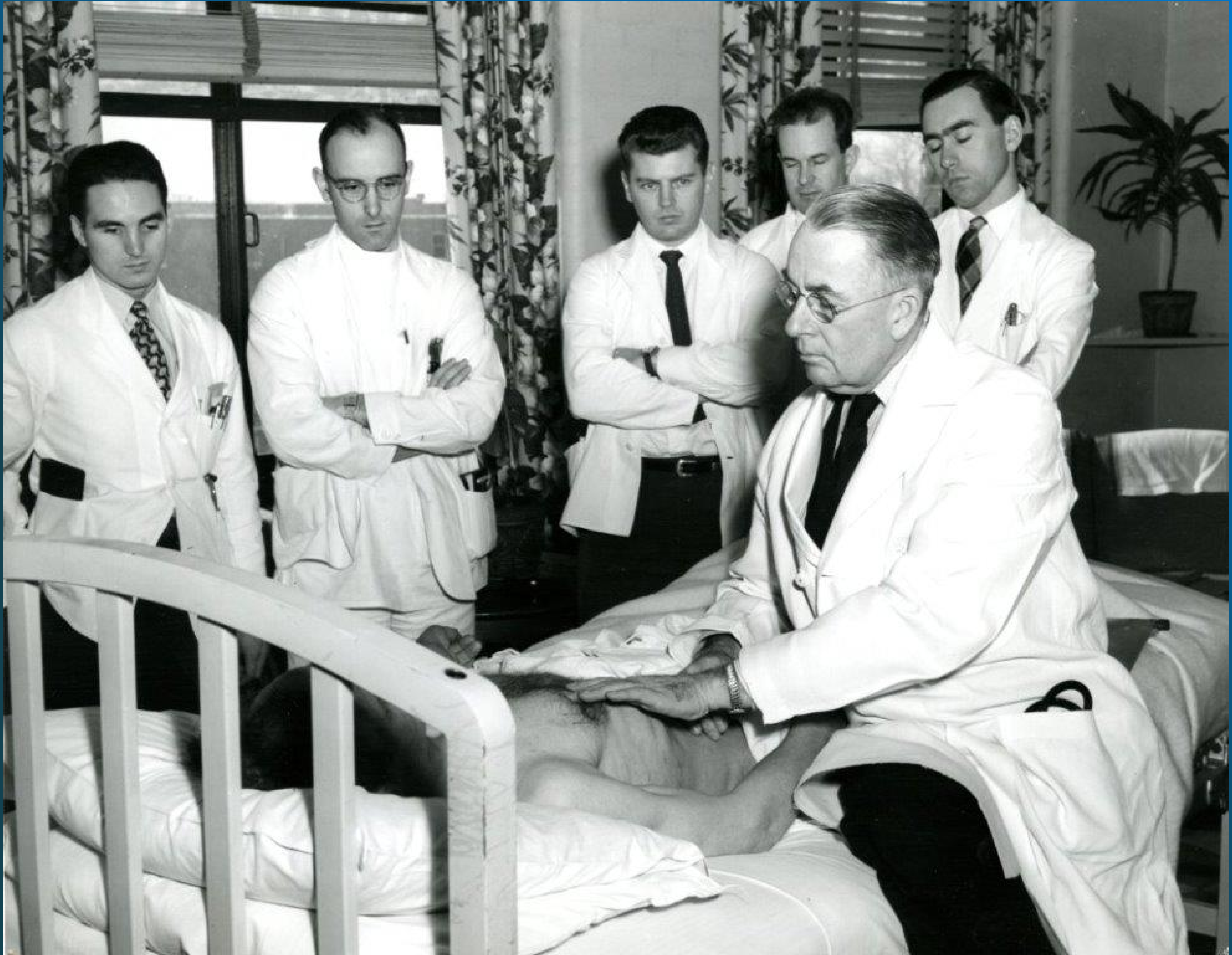

Biopsychosocial (Bio-psycho-social) Model of Health and Disease

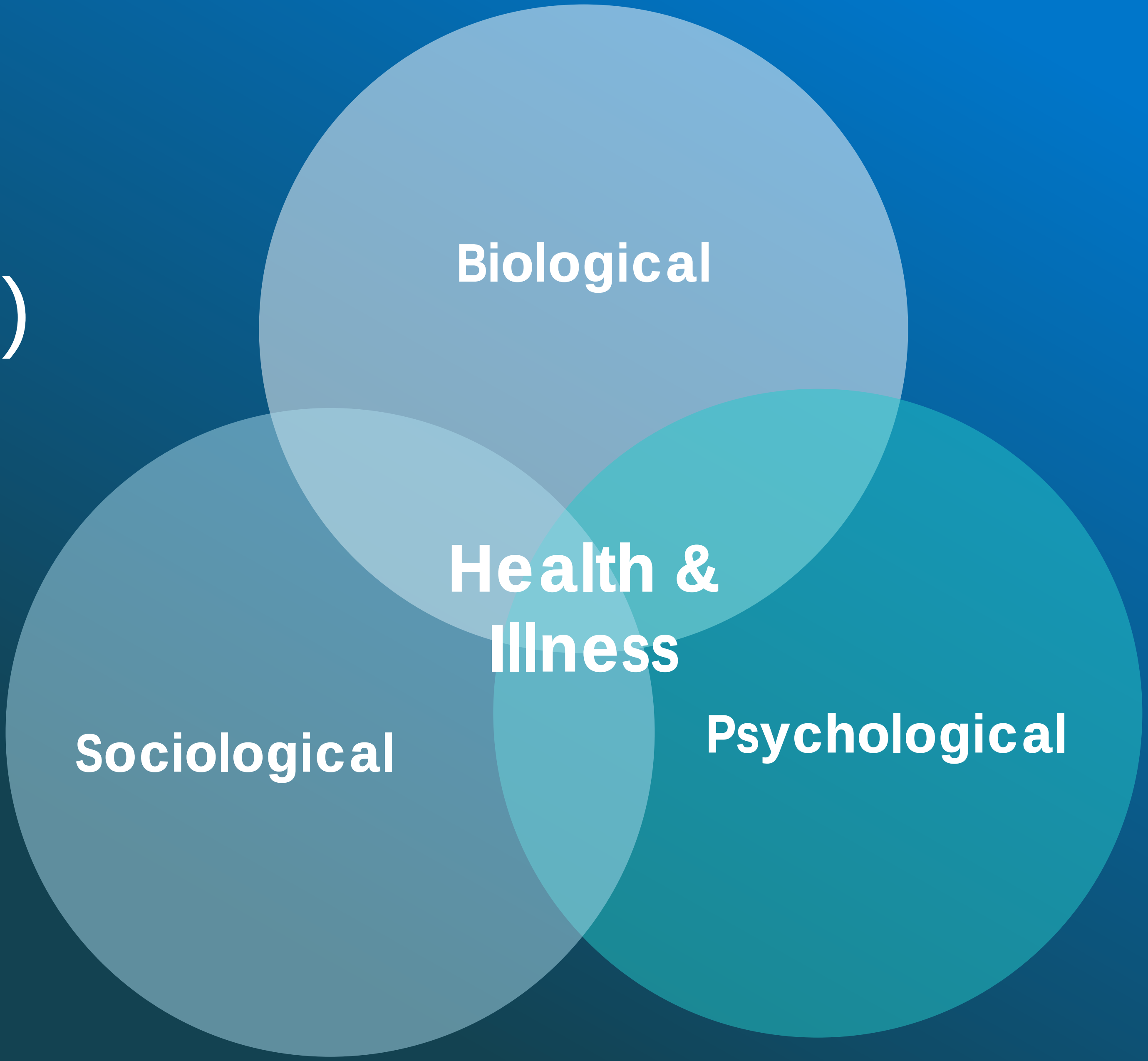

Biopsychosocial Approach

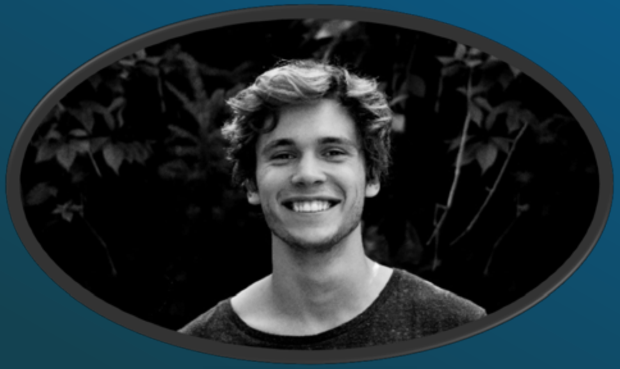

Biosphere
Society/Nation
Culture
Community
Family
Relationships

Person/Patient

Nervous System
Organs
Tissues
Cells
Molecules
Atoms

George Engel, MD

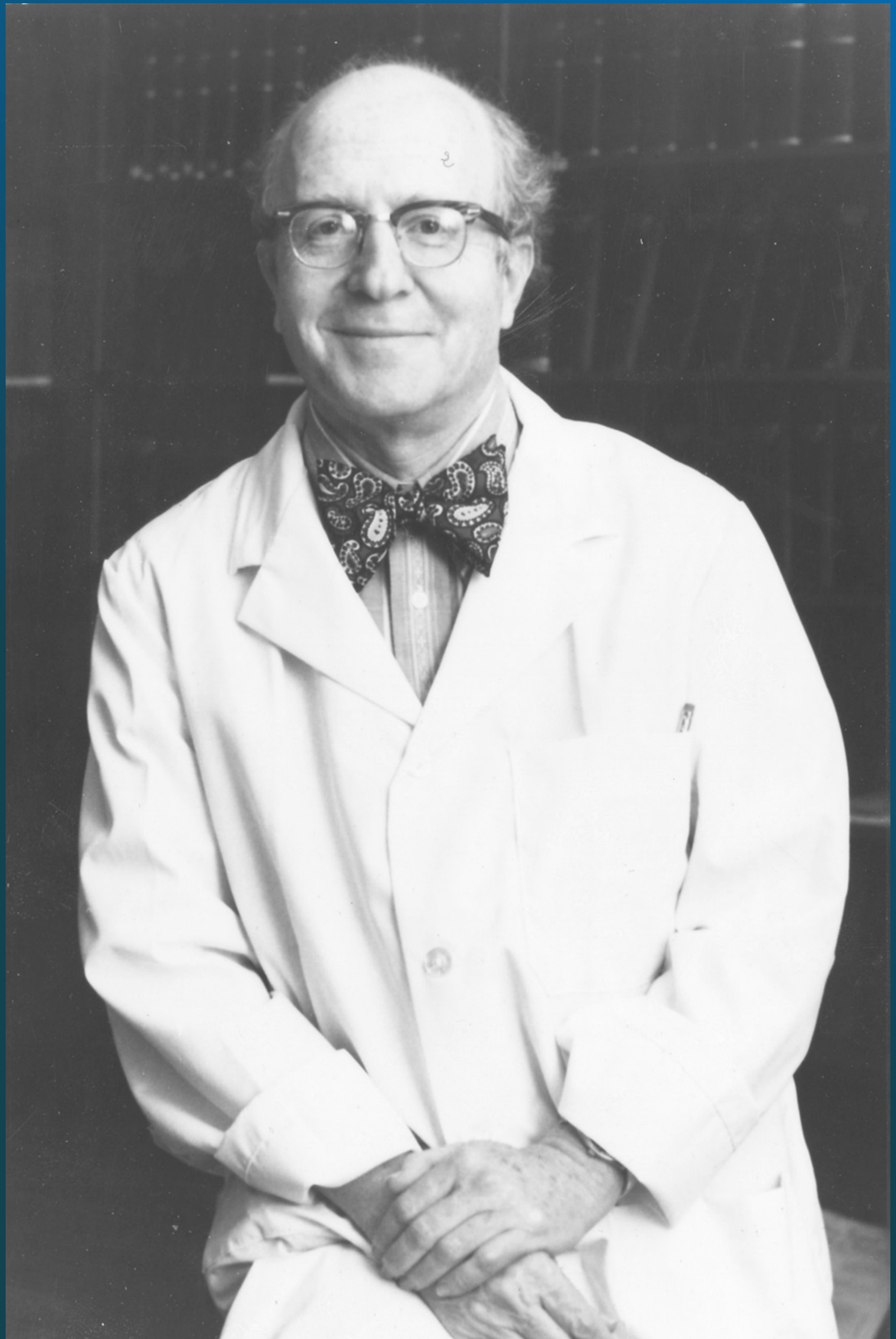

John Romano, MD

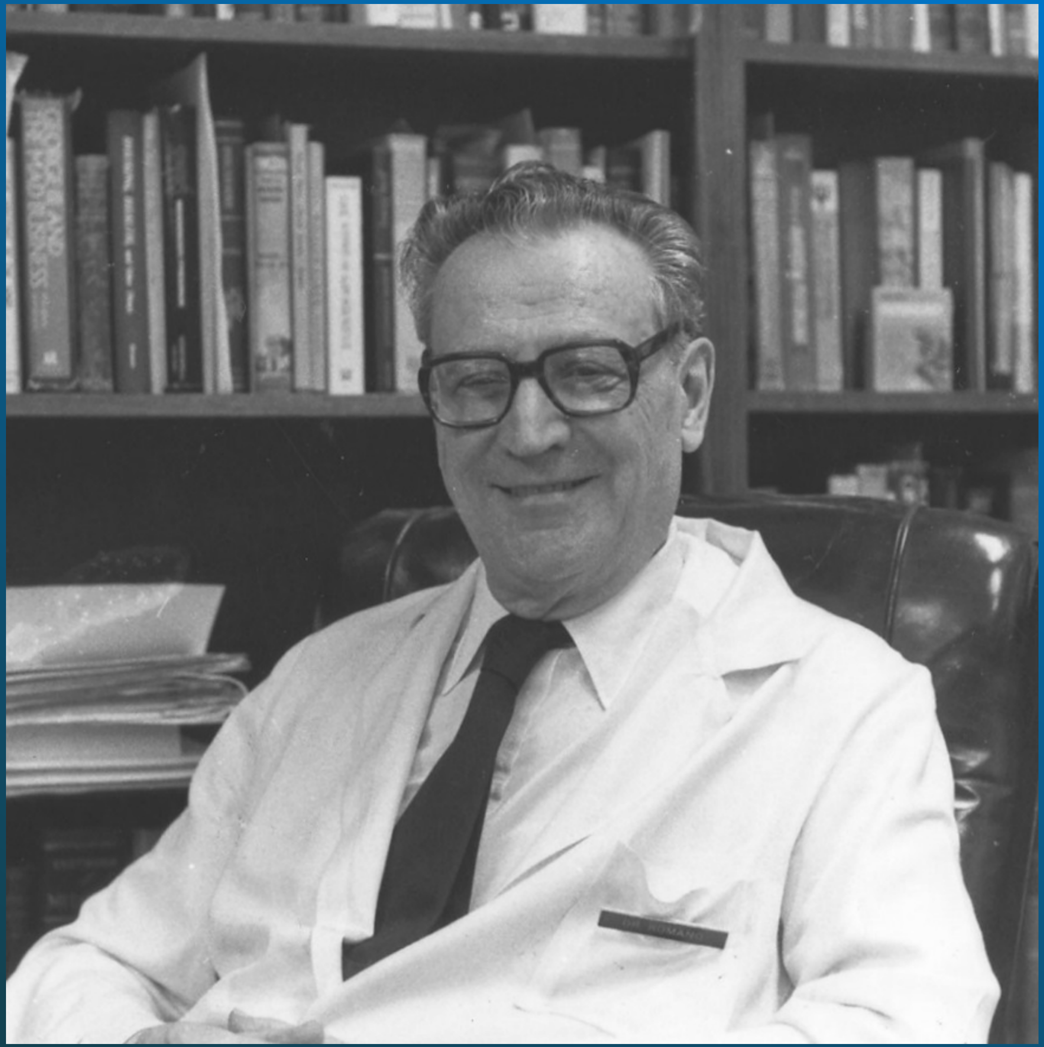

George Engel, MD

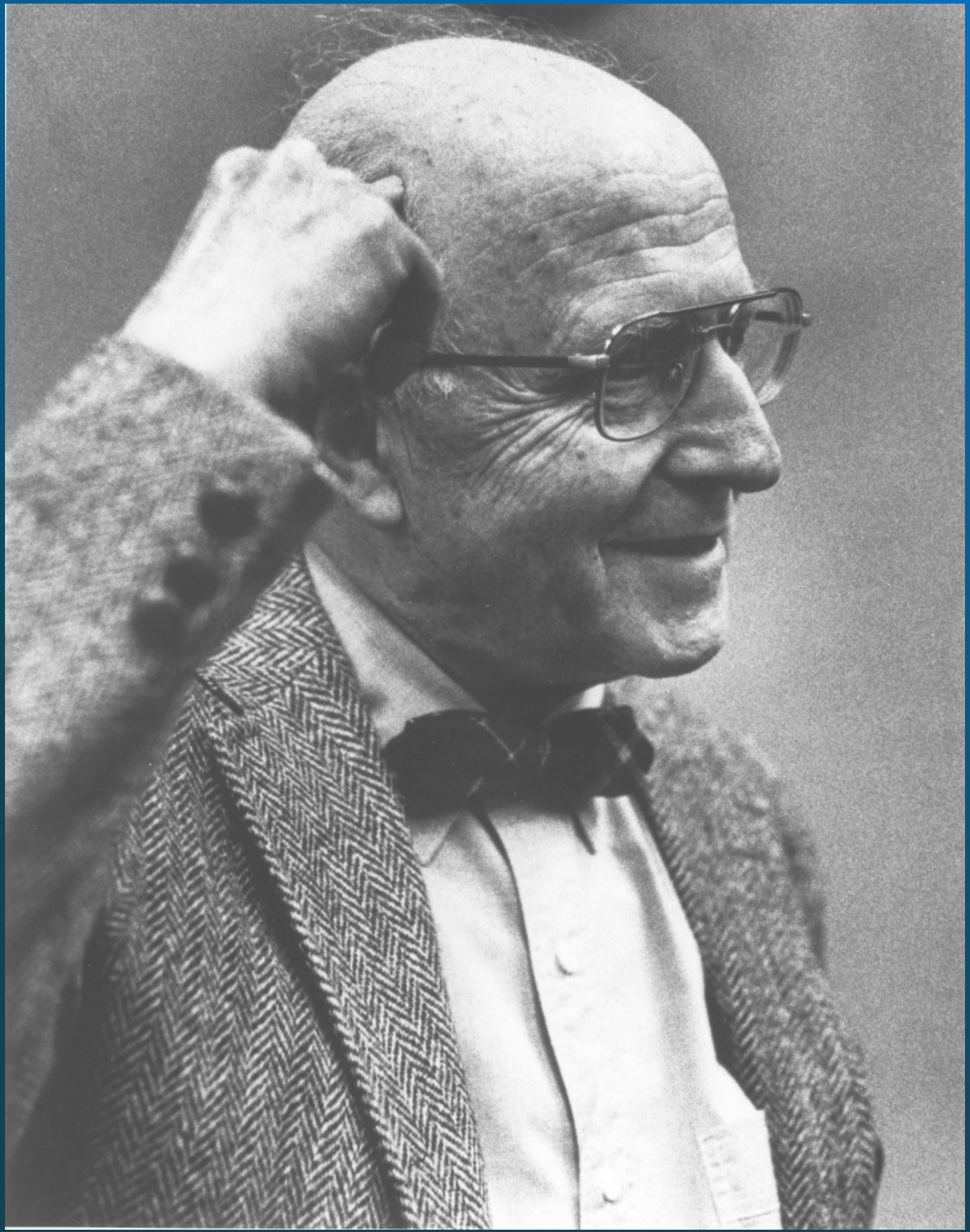

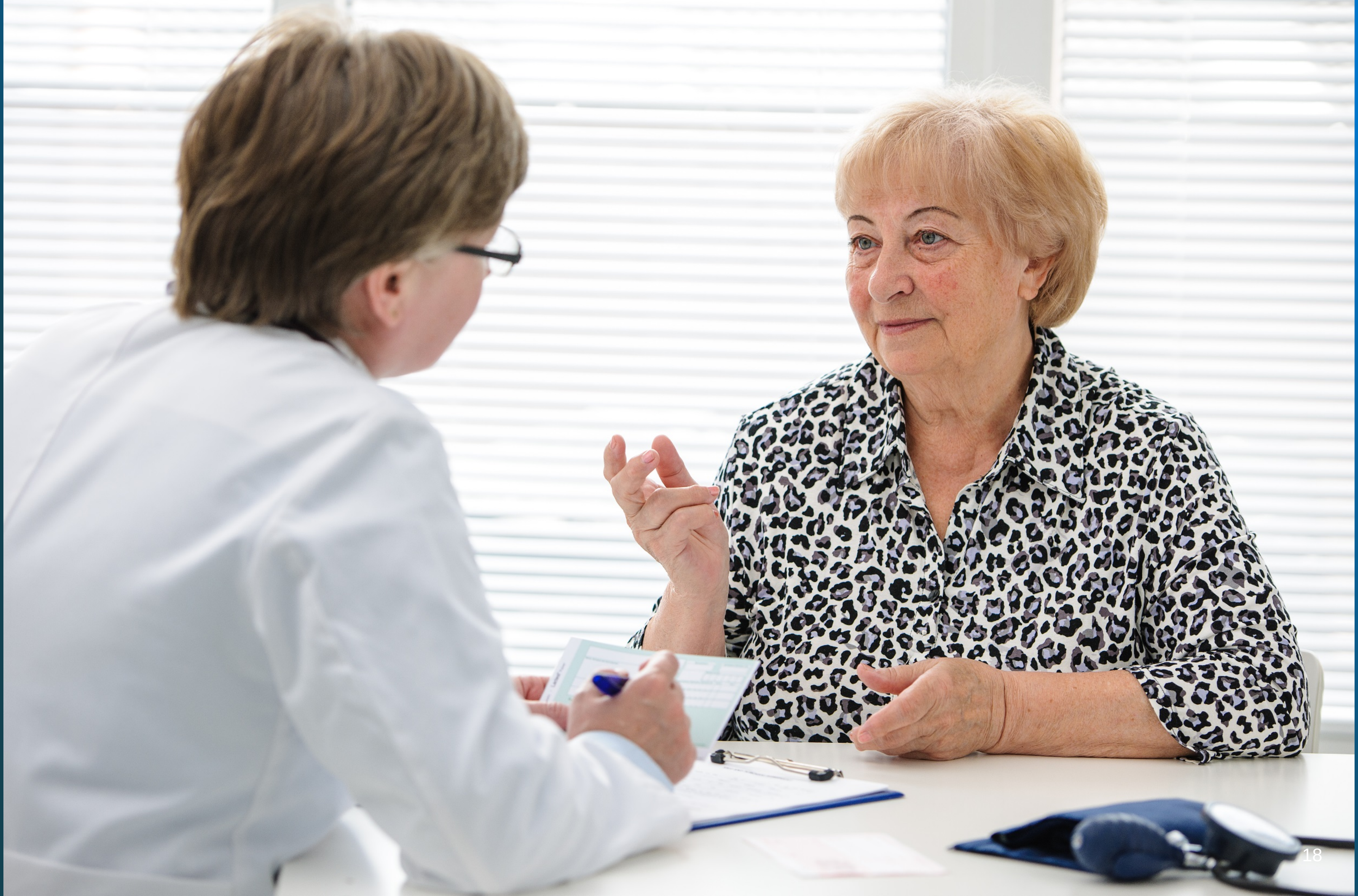

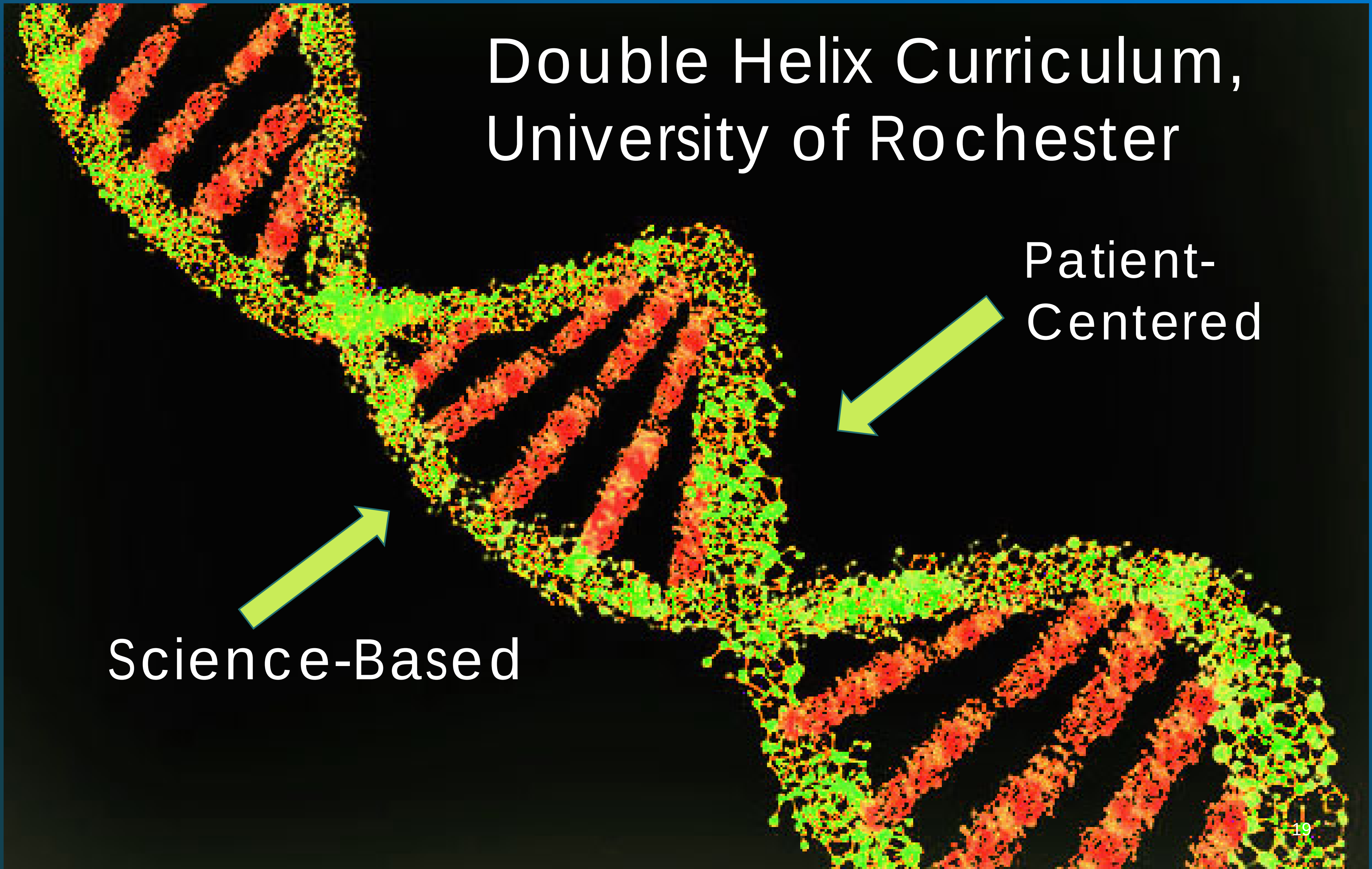
Double Helix Curriculum,
University of Rochester
Patient-
Centered
Science-Based

Ronald M. Epstein, MD – Mindful Practice

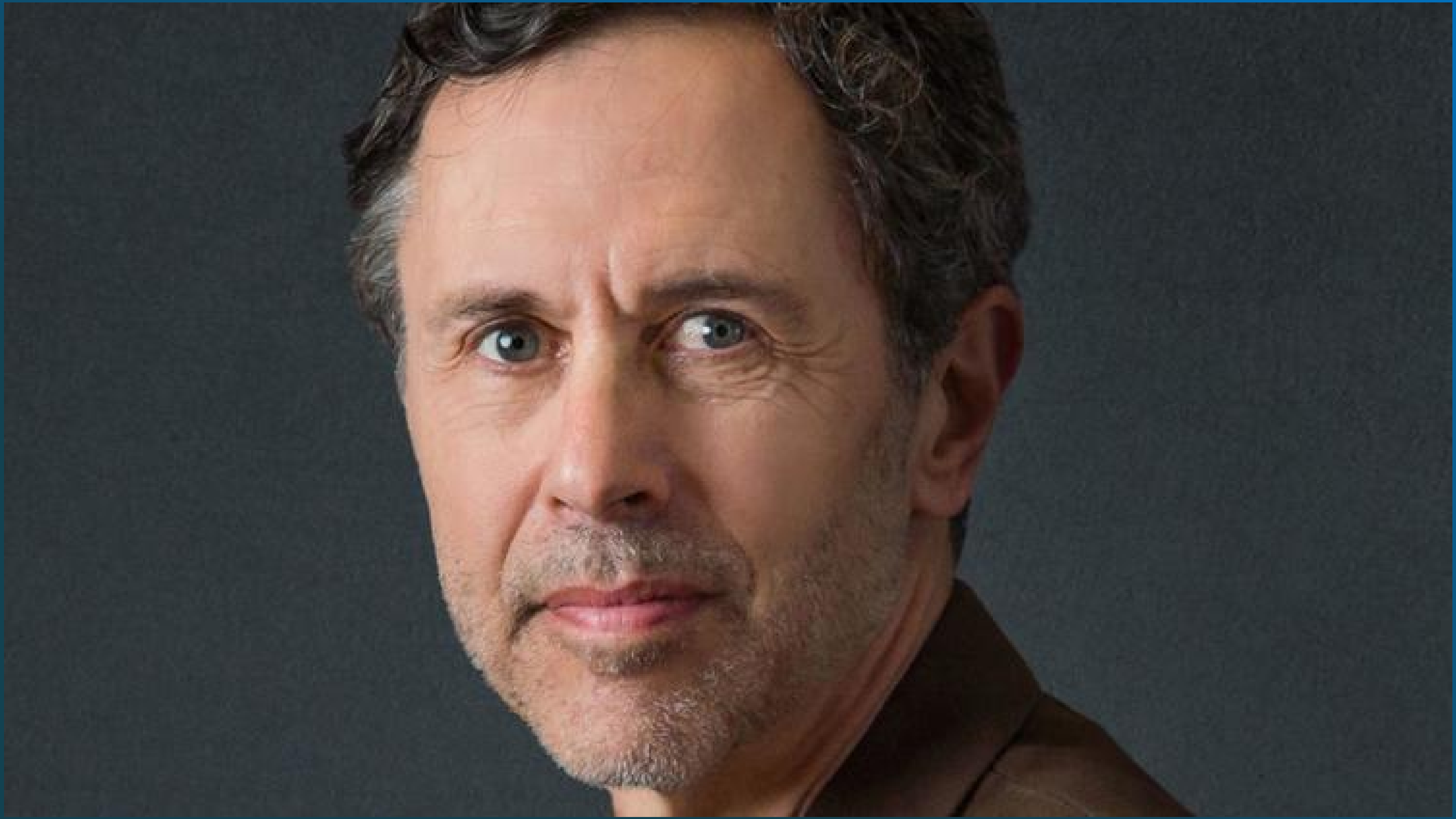

Stephen S. Reardon, photographer

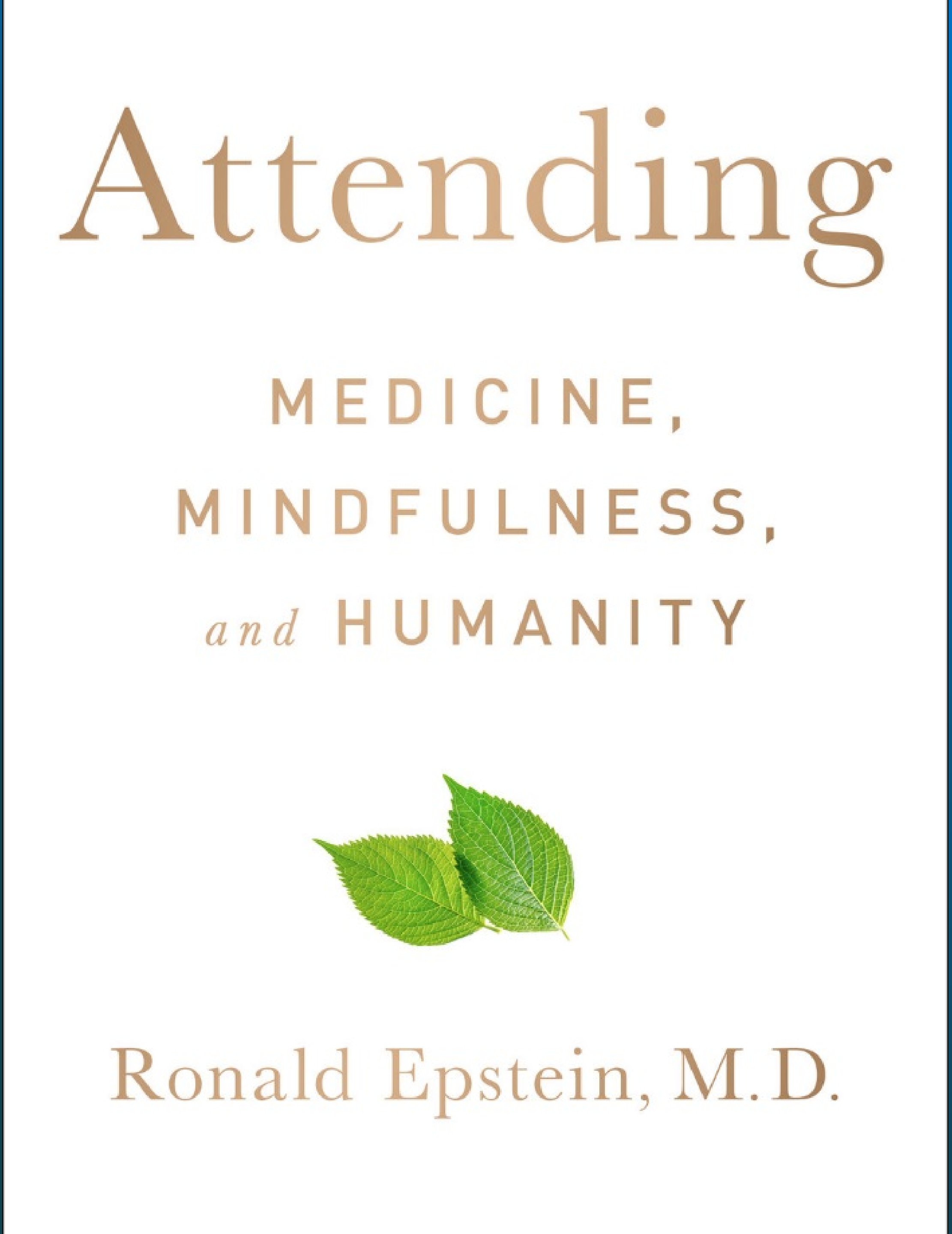
Attending
MEDICINE,
MINDFULNESS,
and HUMANITY
Ronald Epstein, M.D.

Register Today!

Mindful Practice Workshop
Enhancing Quality of Care, Quality of Caring and Resilience

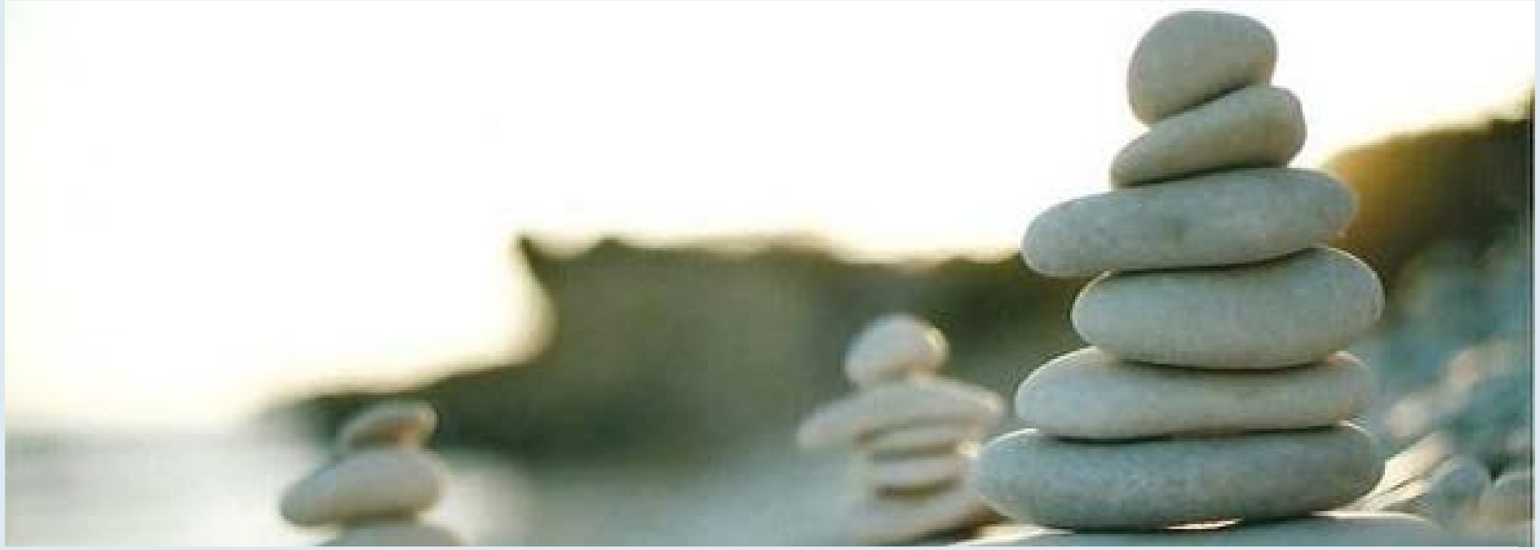

October 26-29, 2016

Sponsored by: The Department of Family Medicine - Mindful Practice Programs
Hosted by: The Center for Experiential Learning

A 4-day, retreat-like workshop designed to improve the quality of care that clinicians provide while improving their own resilience and well-being. It offers an experiential learning environment, with a focus on developing the capacity for self-awareness in stressful and demanding situations.

[illegible]

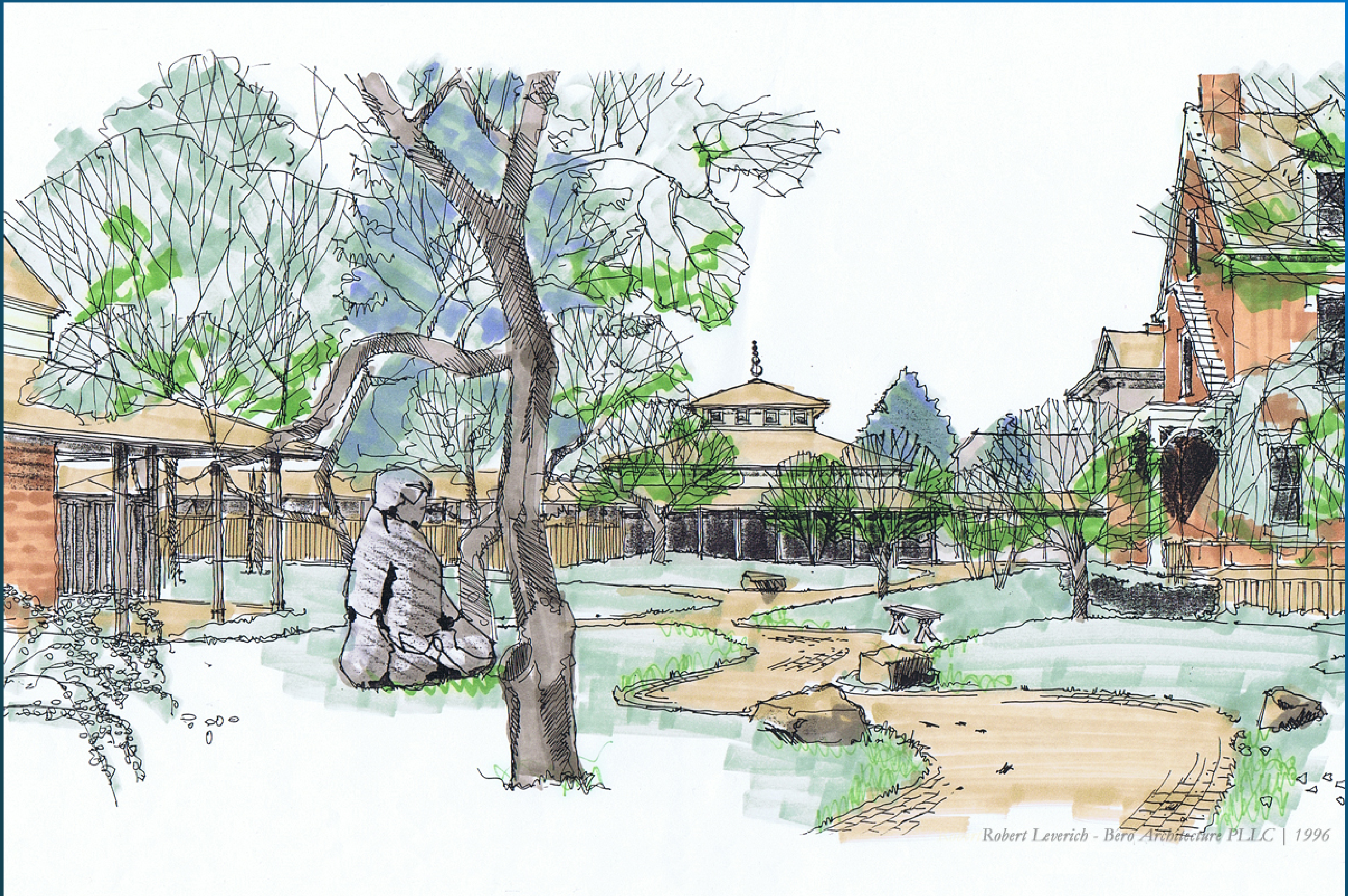

Robert Leverich; Bero Architecture PLLC; 1996; pre-design

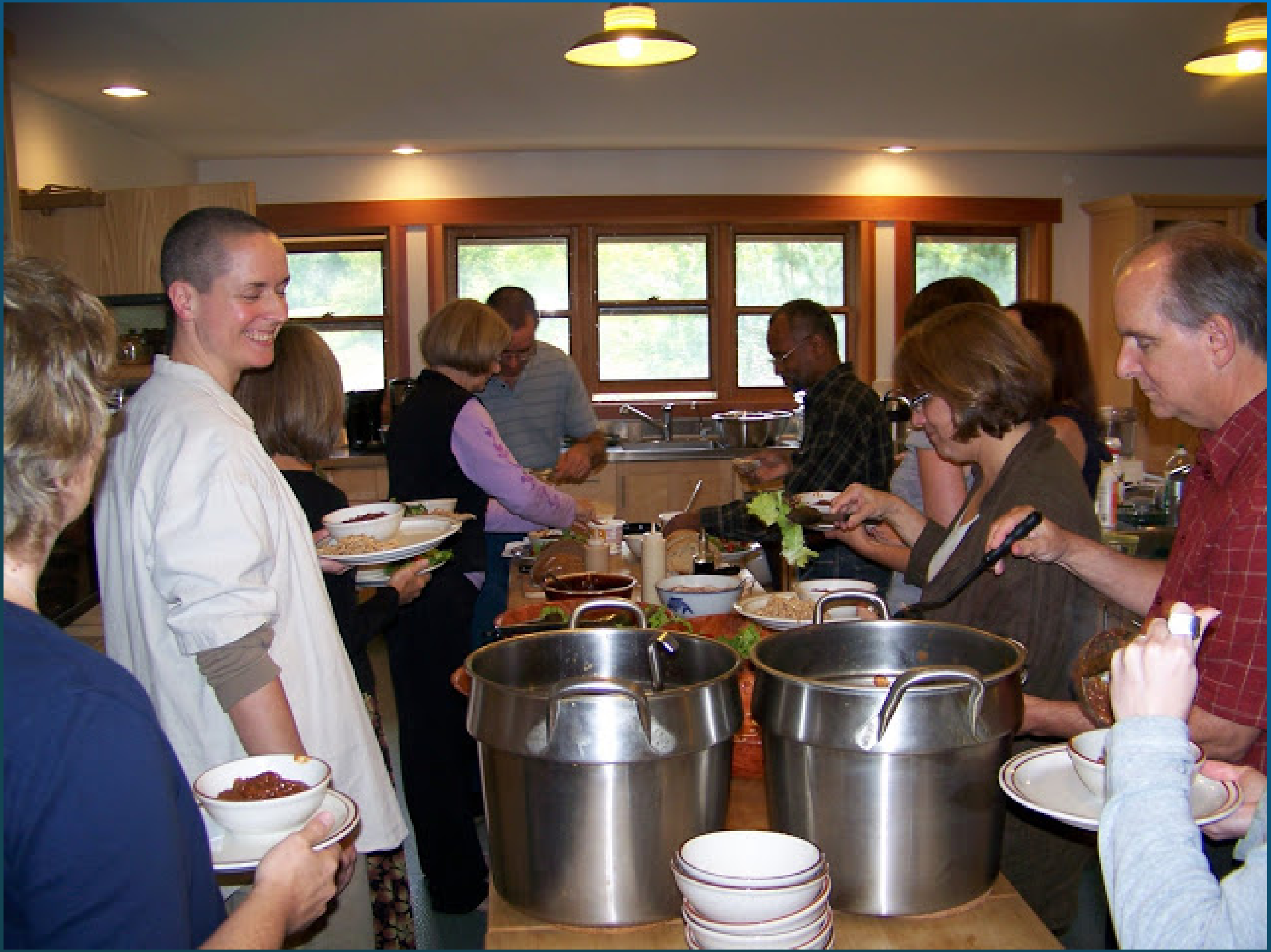

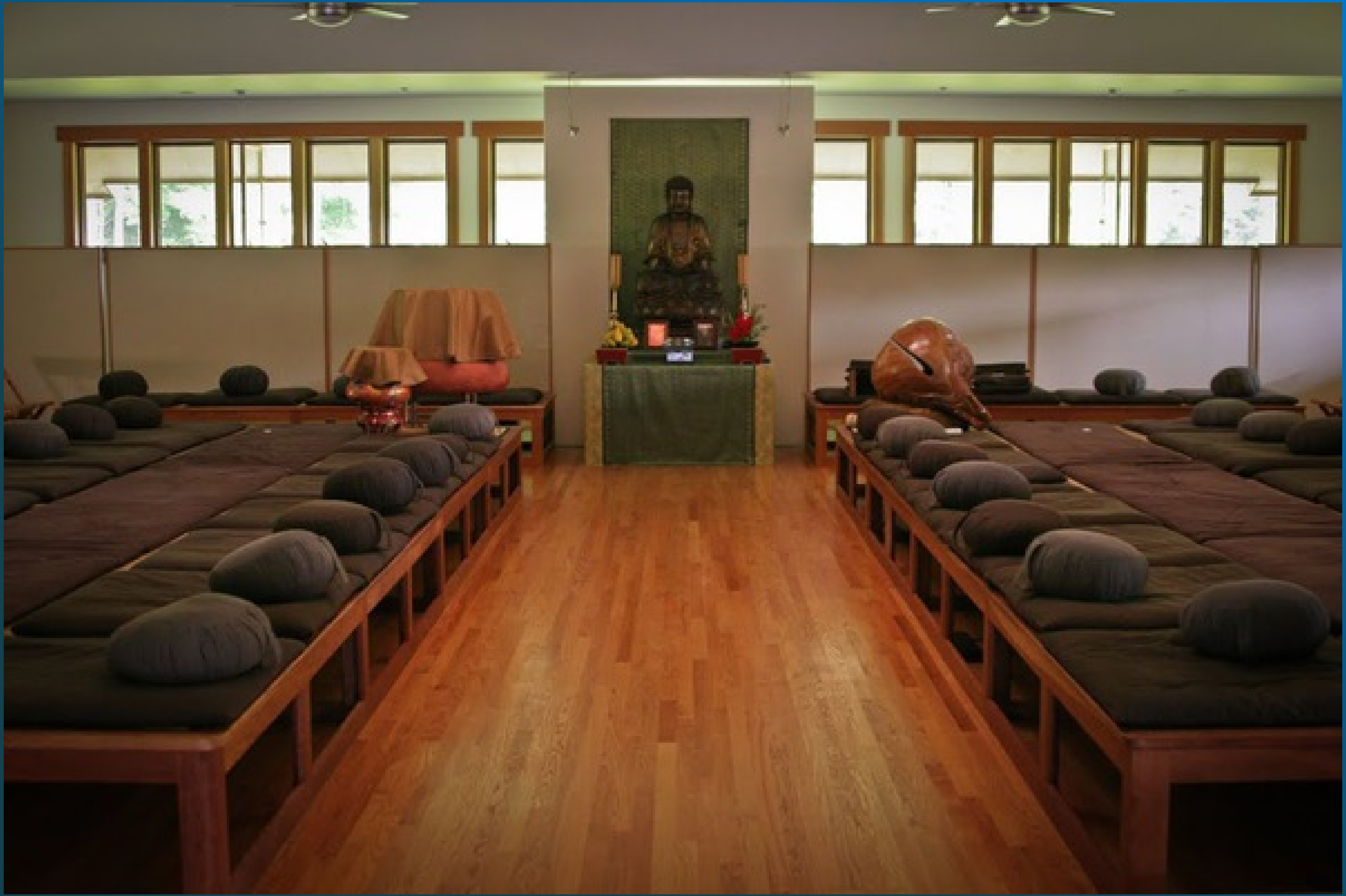

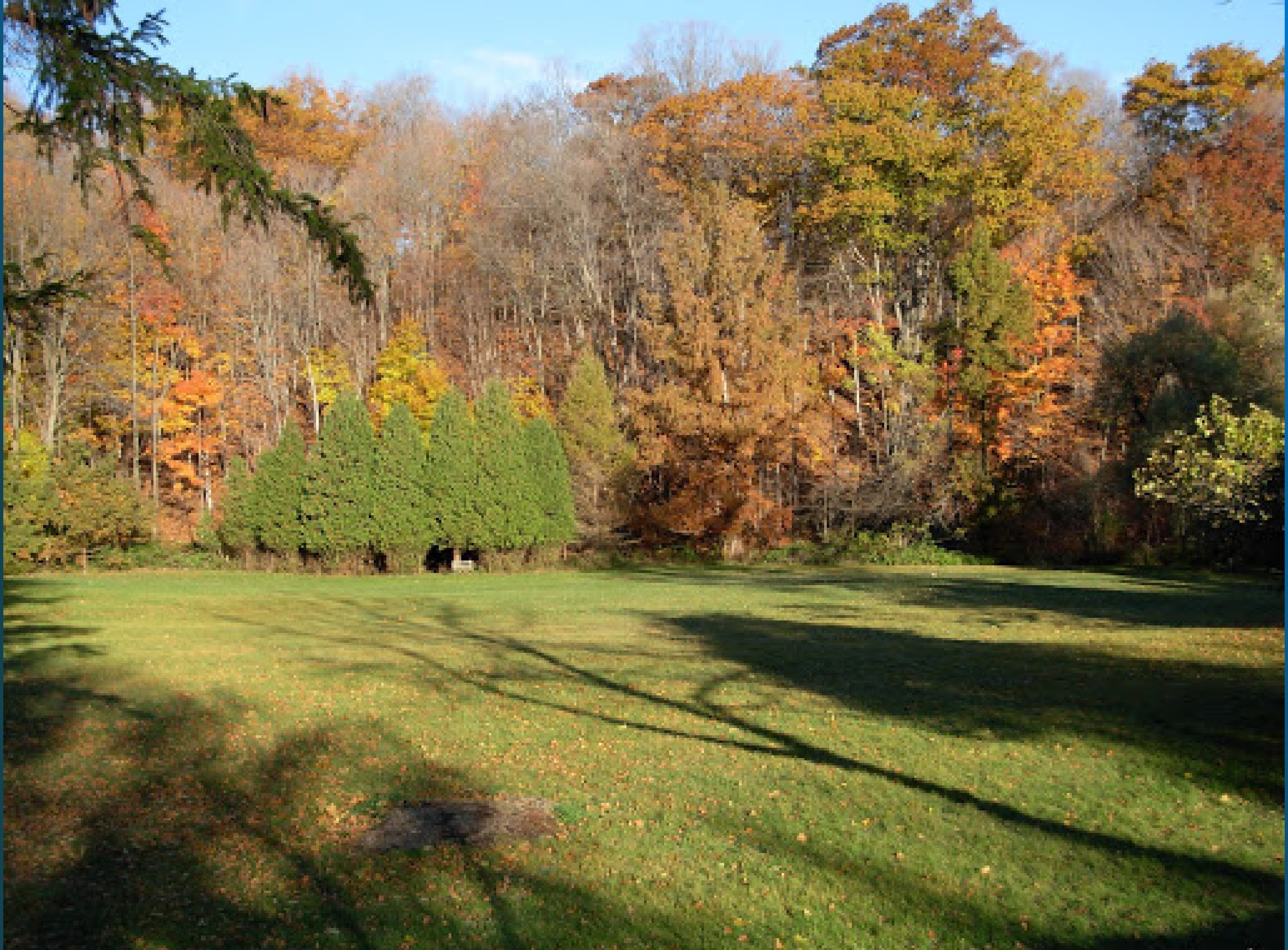

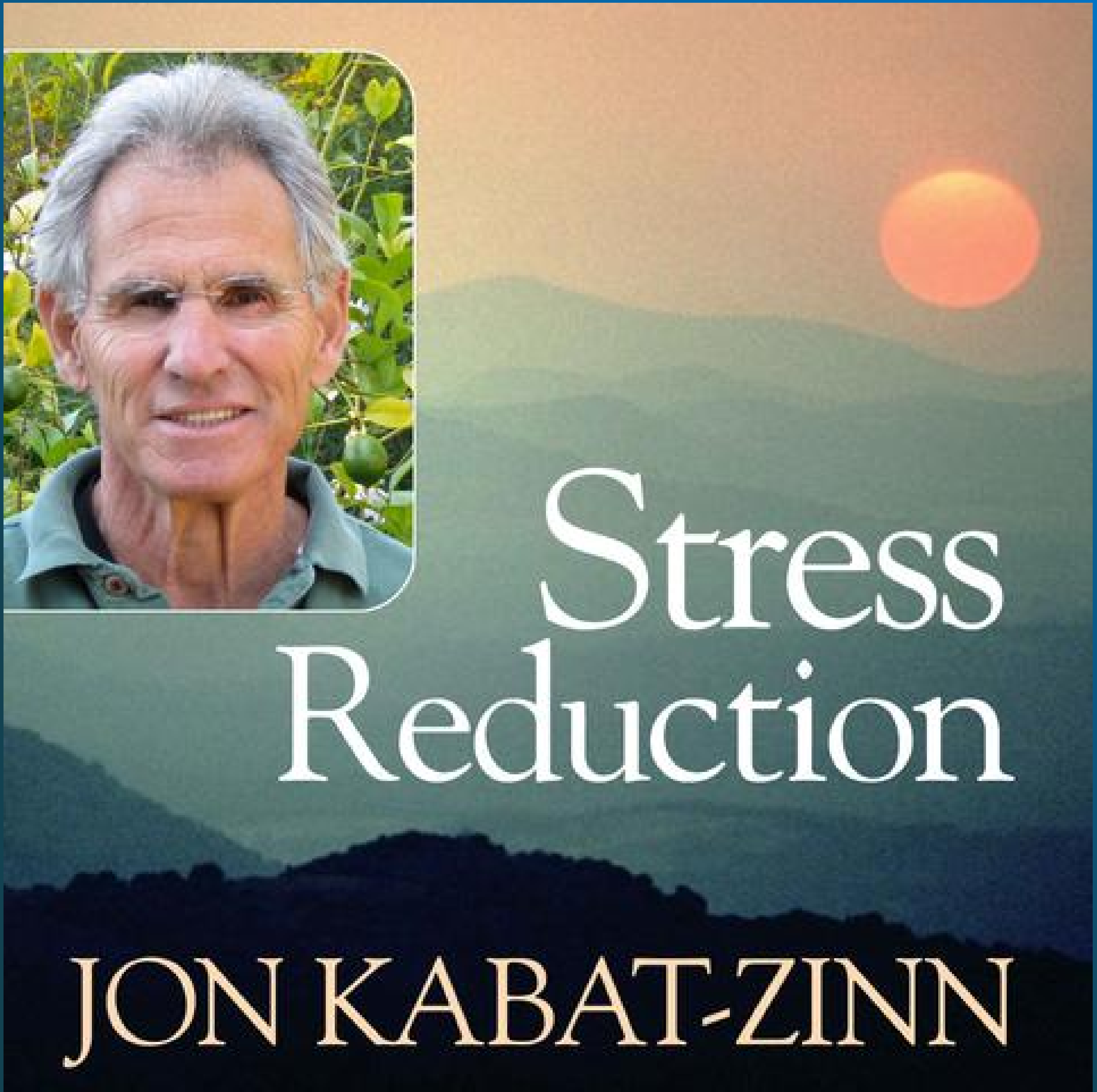
Stress
Reduction
JON KABAT-ZINN

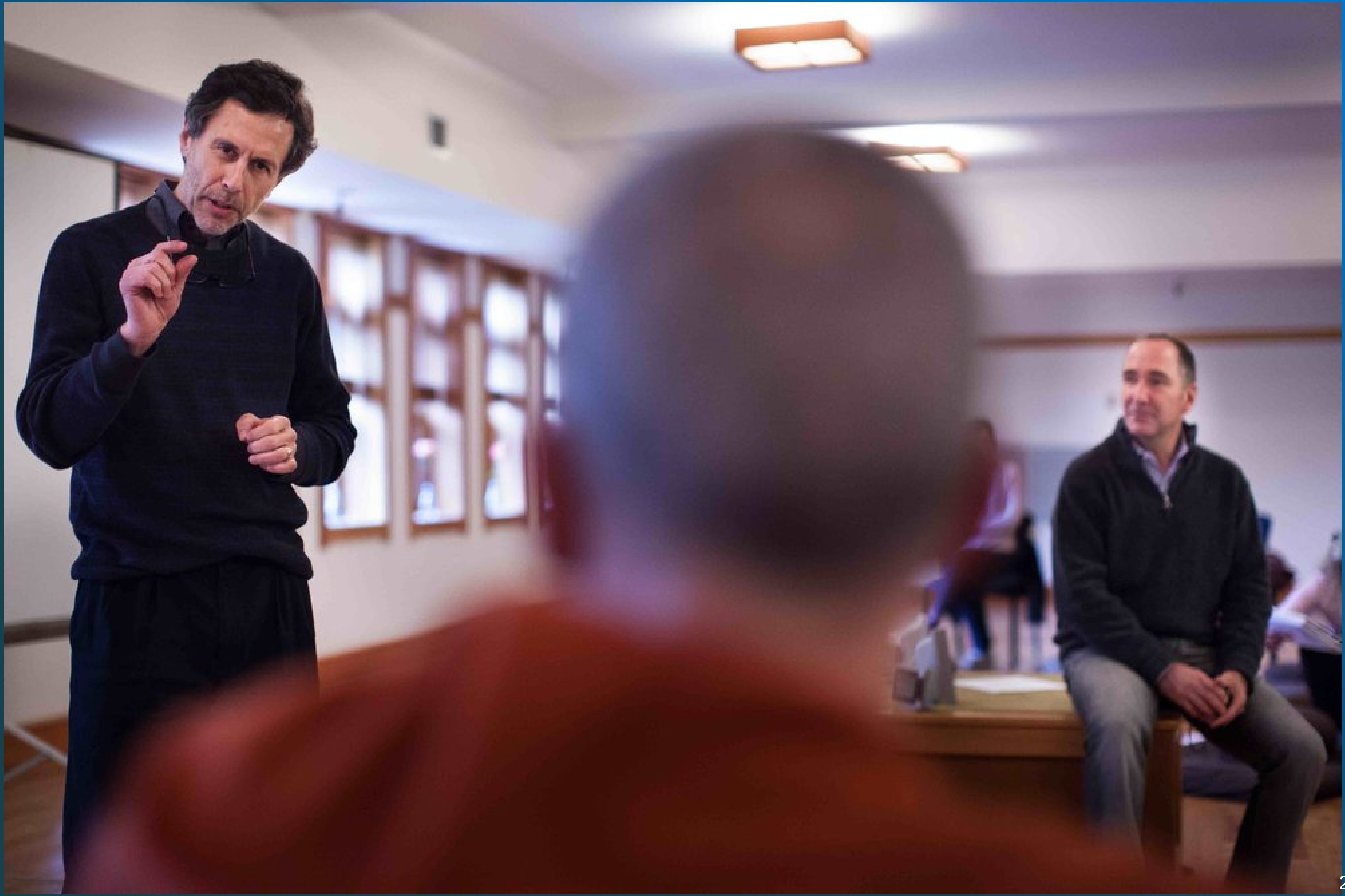

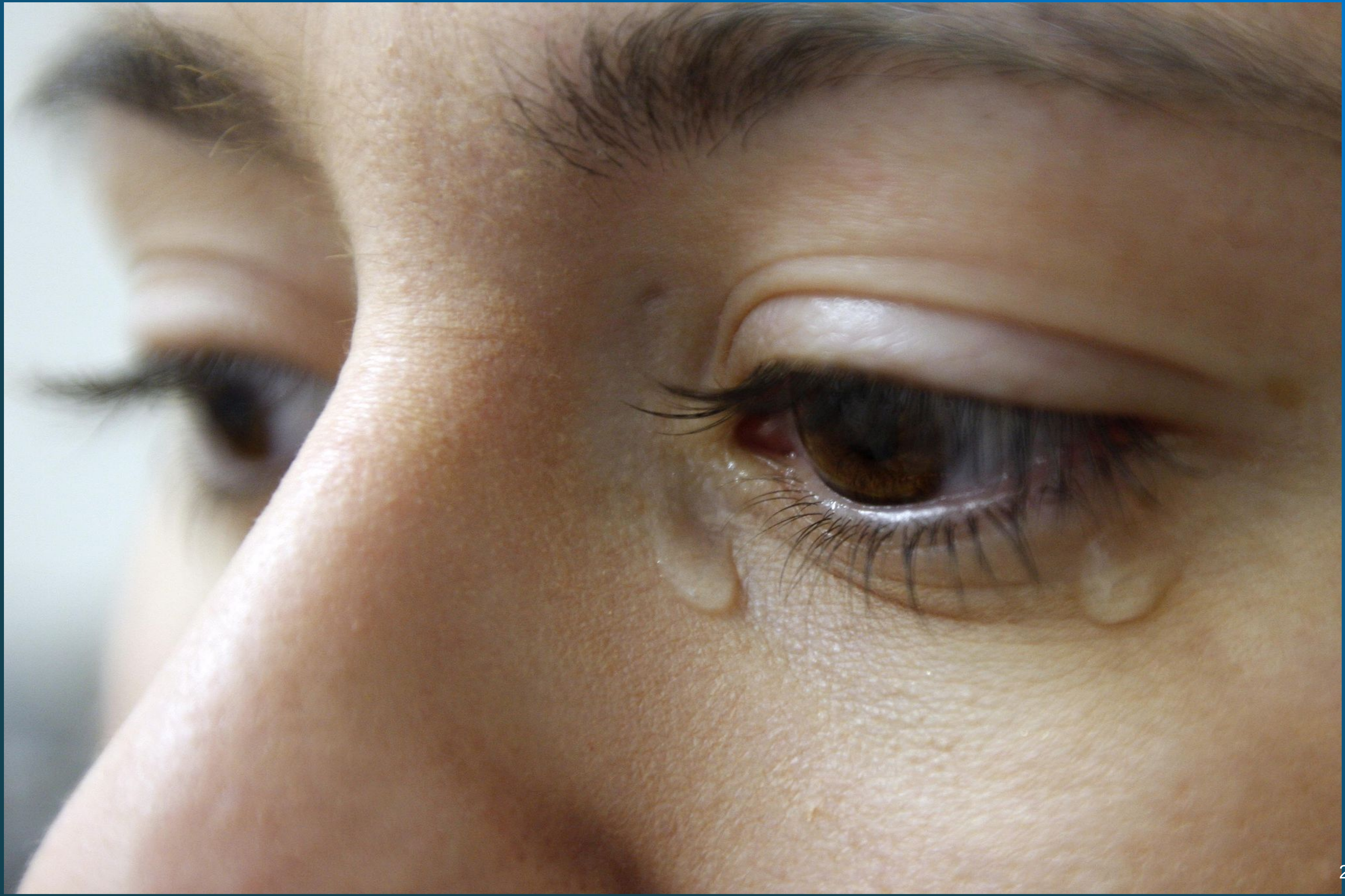

CCARE

ABOUT RESEARCH EDUCATION EVENTS VIDEOS

THE CENTER FOR COMPASSION AND ALTRUISM RESEARCH AND EDUCATION

project compassion

STANFORD

STANFORD SCHOOL OF MEDICINE

THE CENTER FOR COMPASSION AND ALTRUISM RESEARCH AND EDUCATION

HEARTS in Healthcare

the schwartz center

FOR COMPASSIONATE HEALTHCARE

All Healthcare Givers Invited

the schwartz center
Rounds®

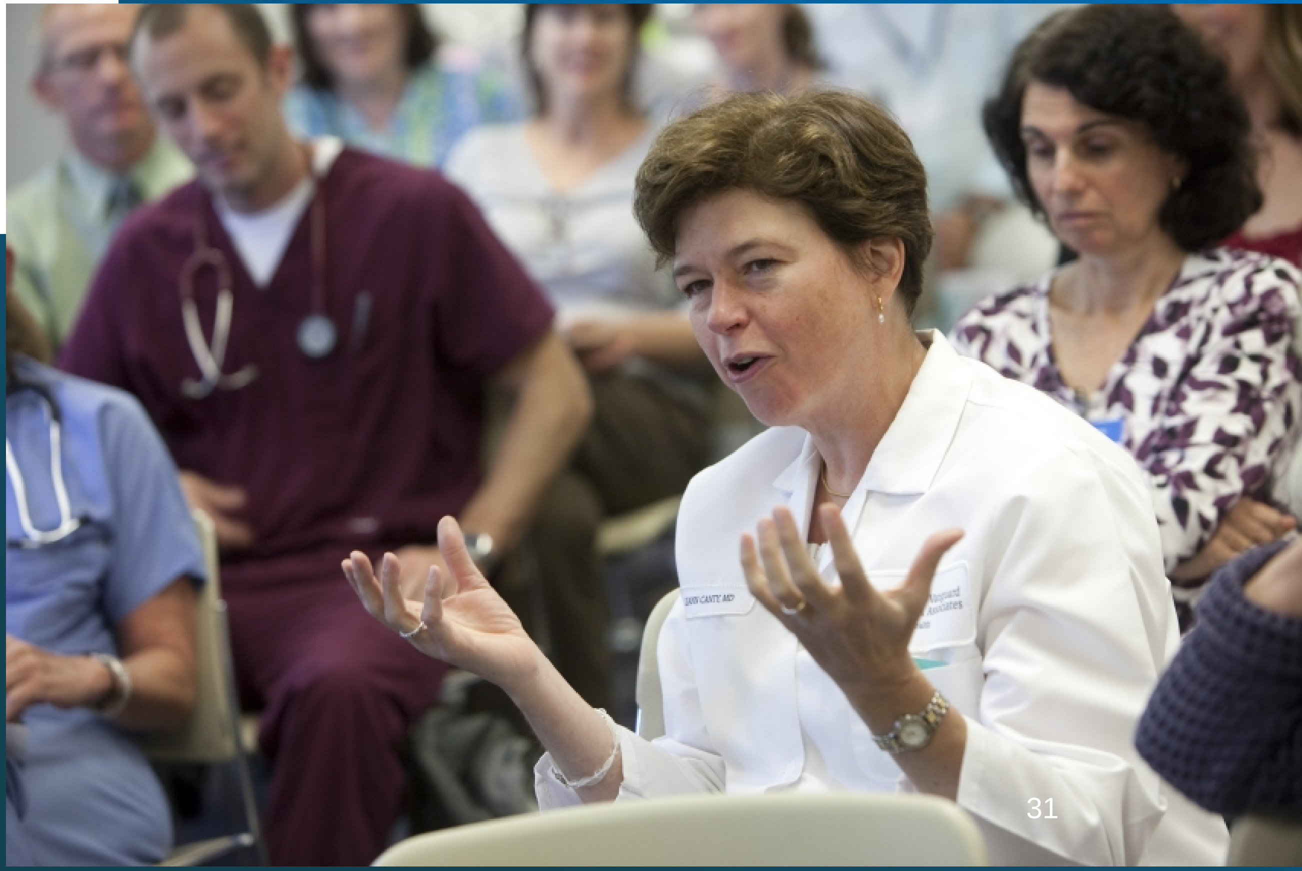

marc.ucla
mindful awareness research center

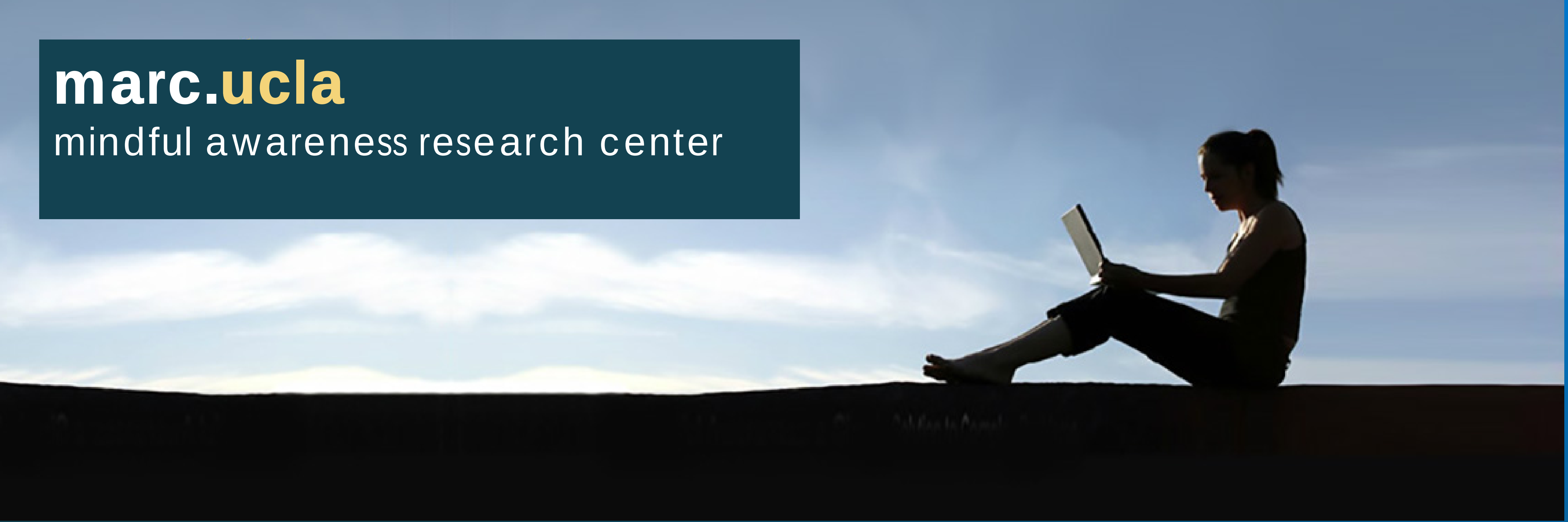

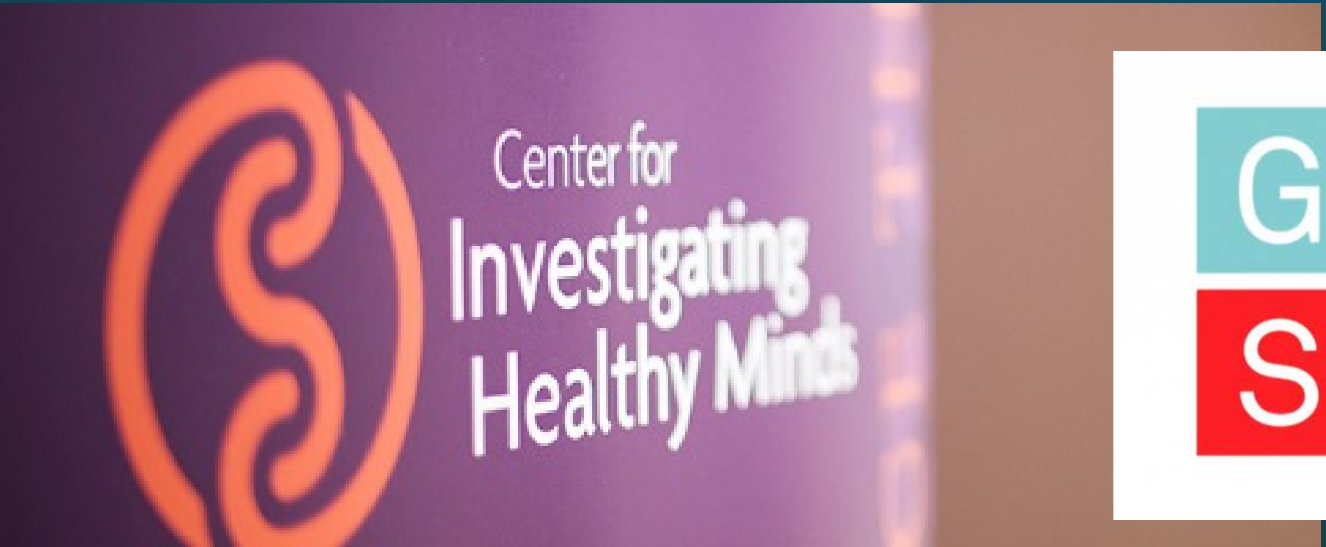

Greater Good Science Center

Berkeley
UNIVERSITY OF CALIFORNIA

Magazine covers with headline articles about mindfulness

Neuroscience of the Mindful Brain

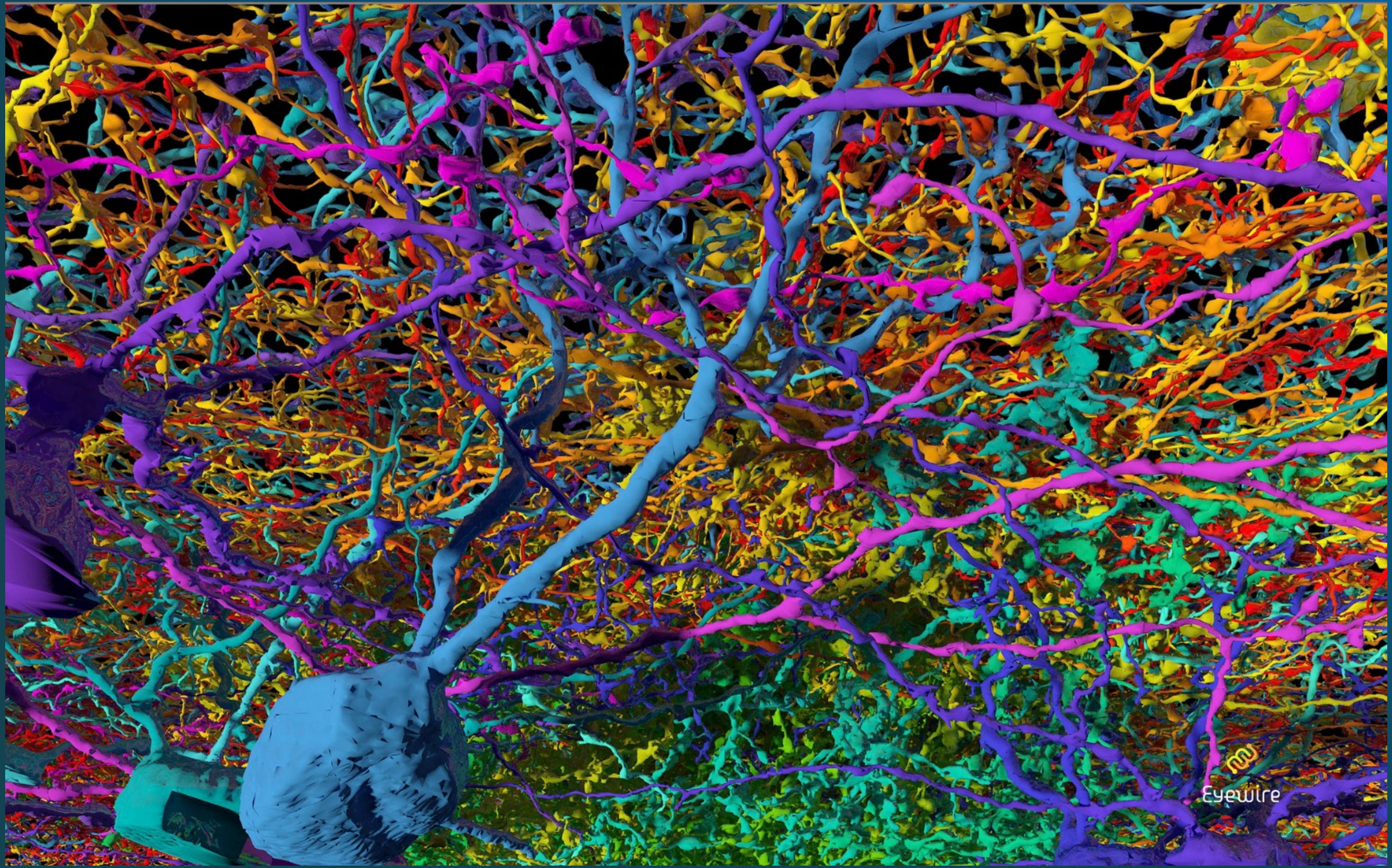

- Contemplative Neuroscience
- Affective Neuroscience
- Interpersonal Neurobiology

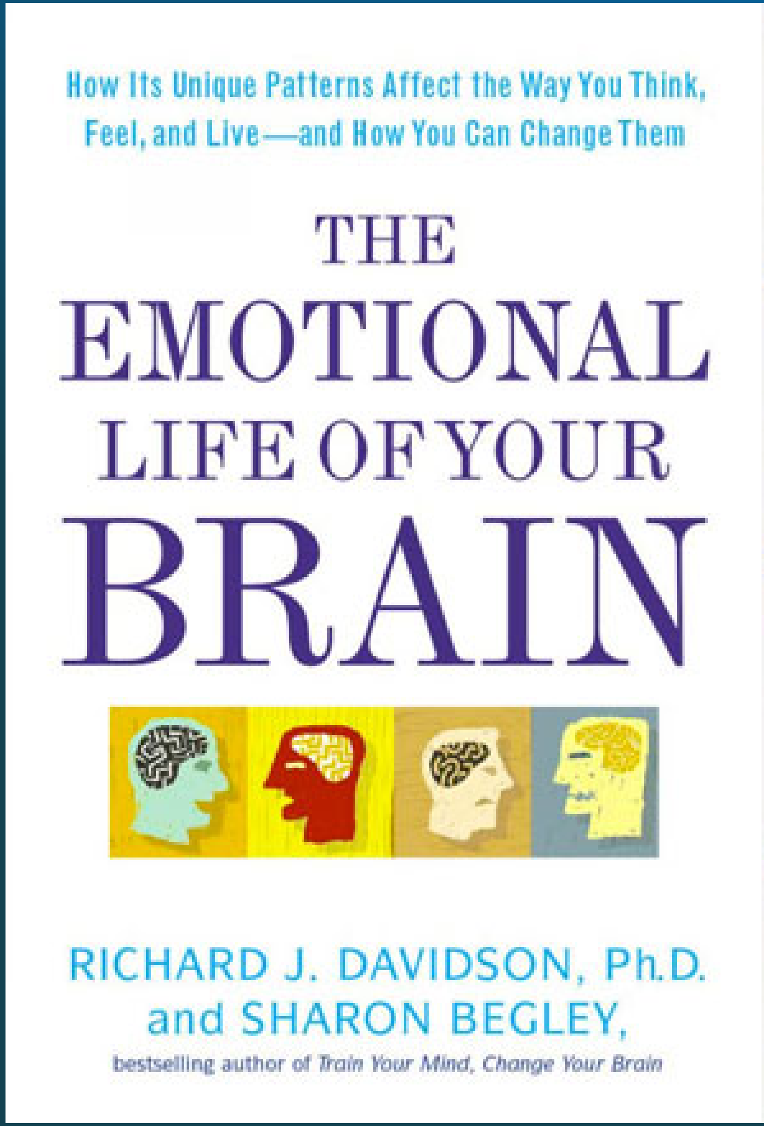
How Its Unique Patterns Affect the Way You Think, Feel, and Live—and How You Can Change Them
THE EMOTIONAL LIFE OF YOUR BRAIN
RICHARD J. DAVIDSON, Ph.D.
and SHARON BEGLEY,
bestselling author of Train Your Mind, Change Your Brain

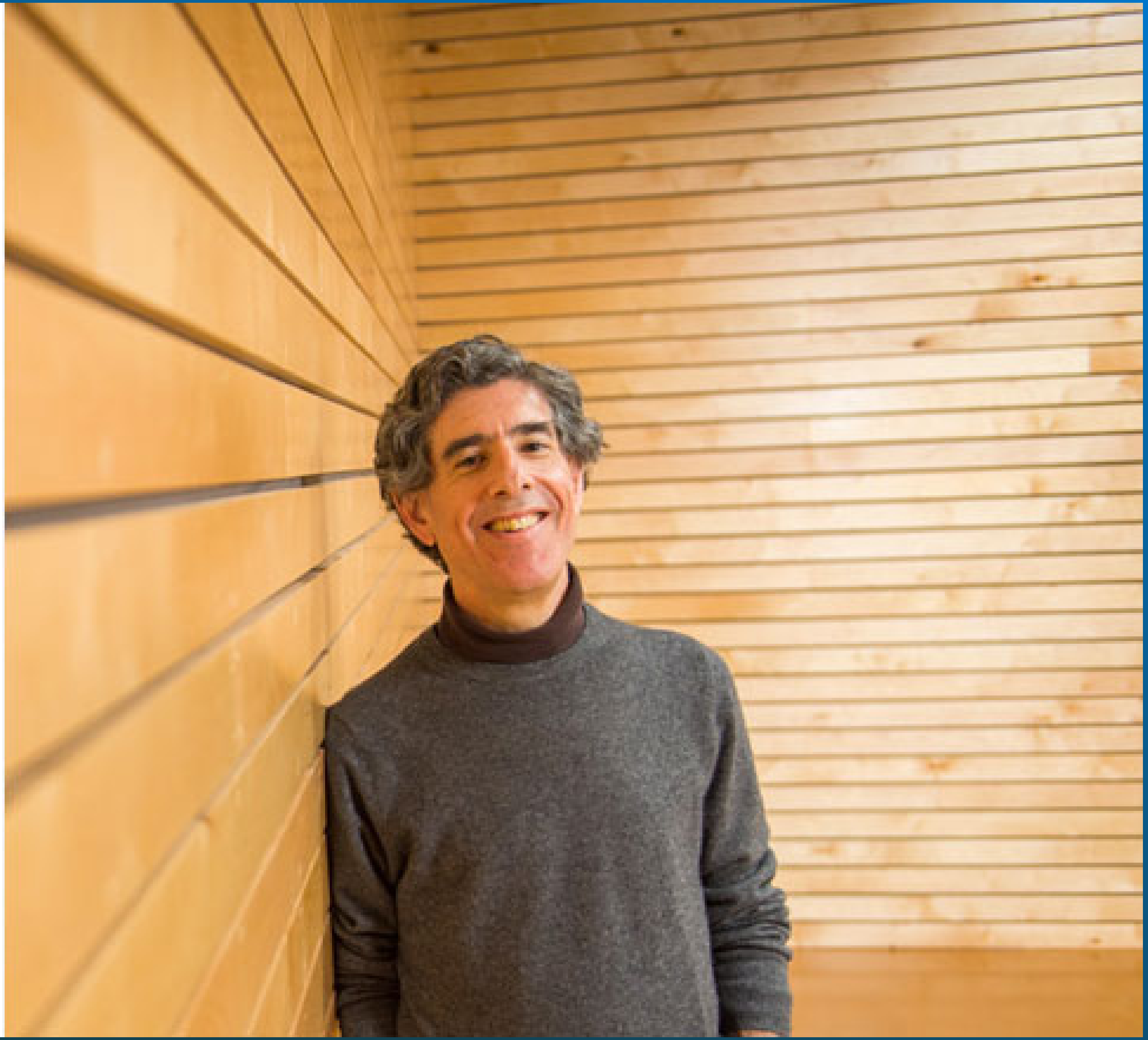

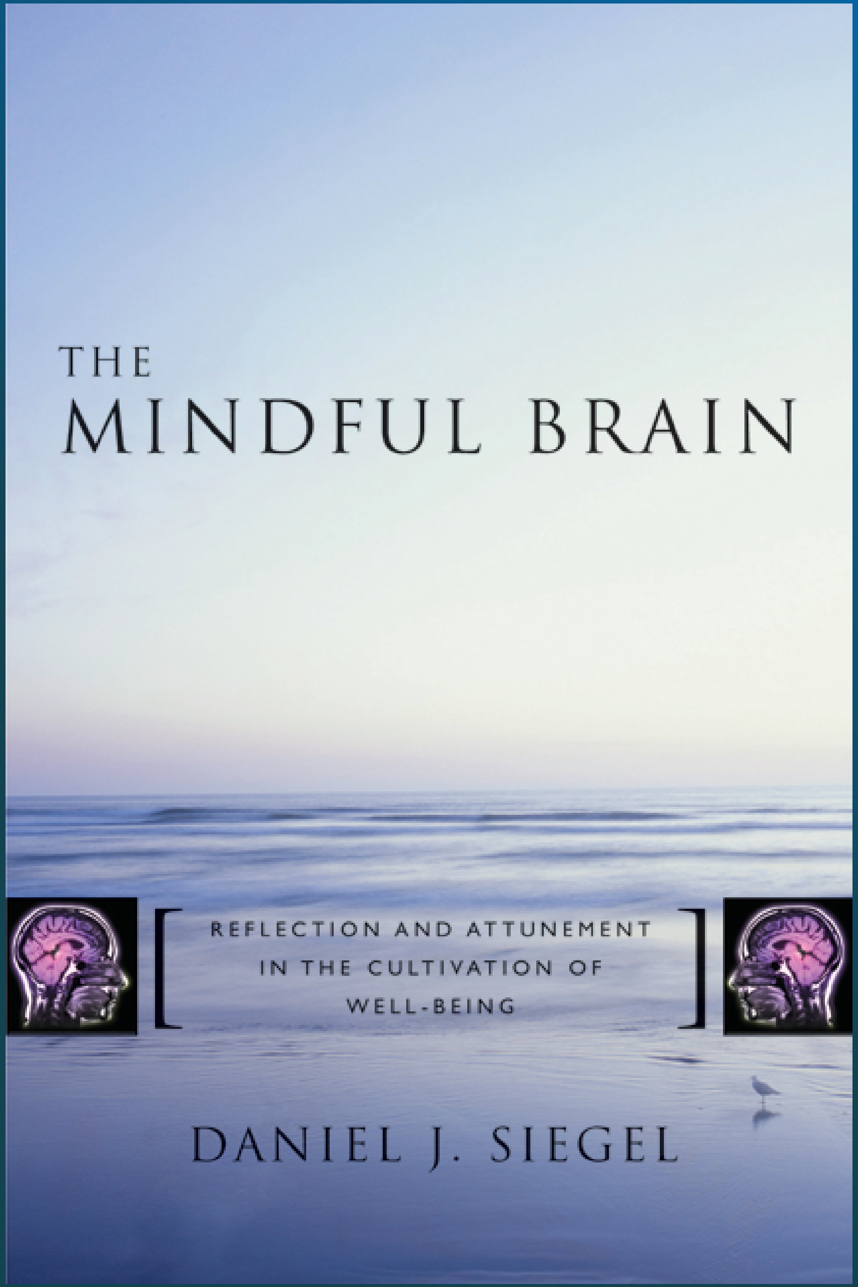
THE
MINDFUL BRAIN
REFLECTION AND ATTUNEMENT
IN THE CULTIVATION OF
WELL-BEING
DANIEL J. SIEGEL

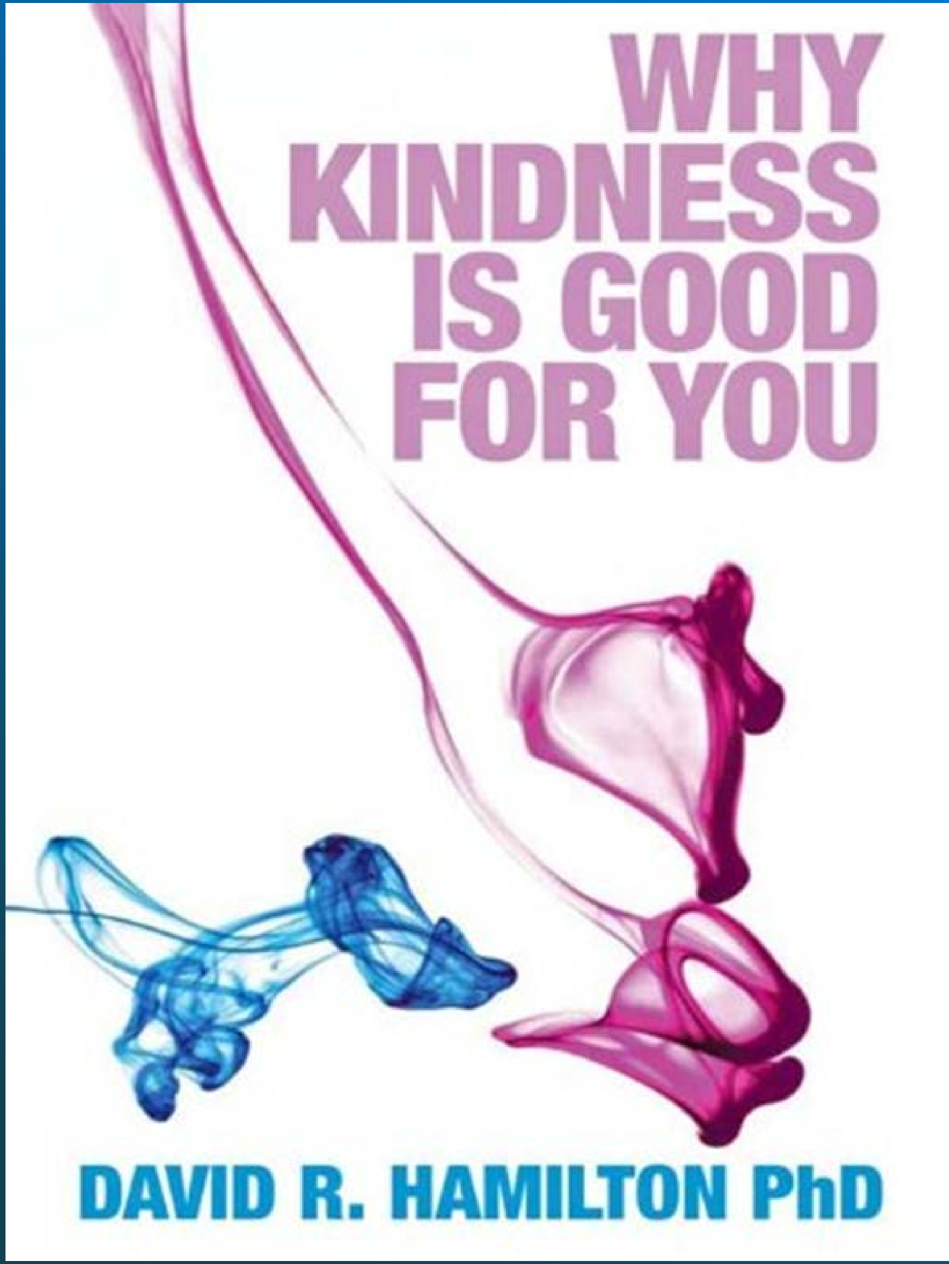
WHY
KINDNESS
IS GOOD
FOR YOU
DAVID R. HAMILTON PhD

Dopamine

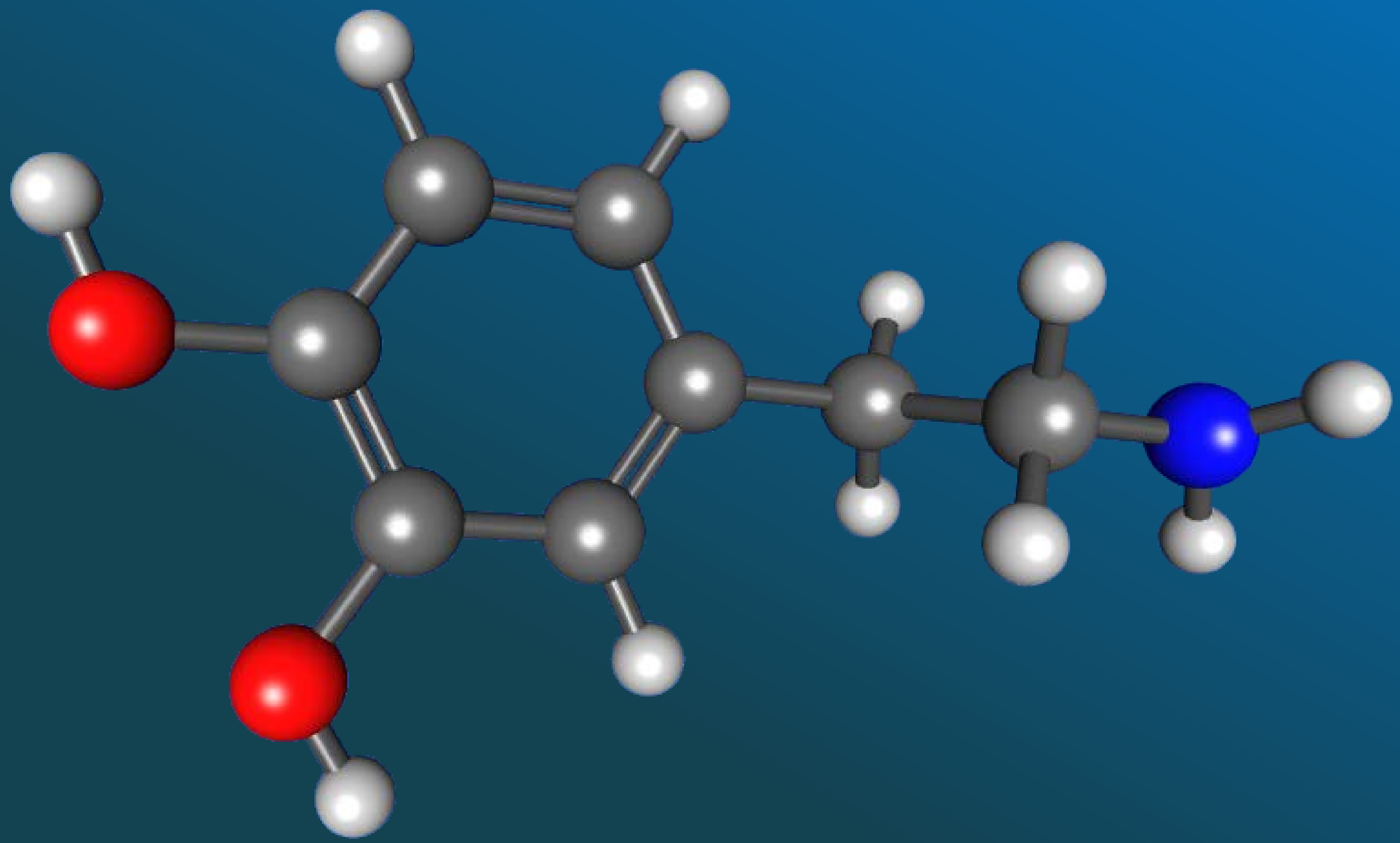

Cortisol ↓ Blood Pressure

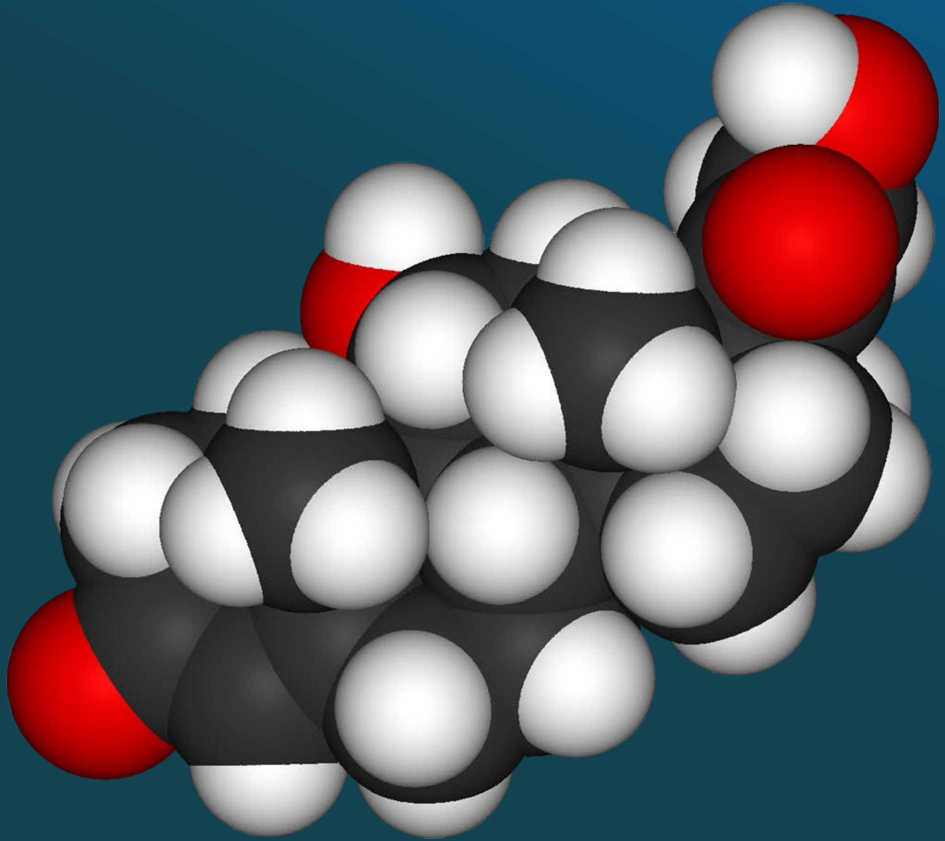

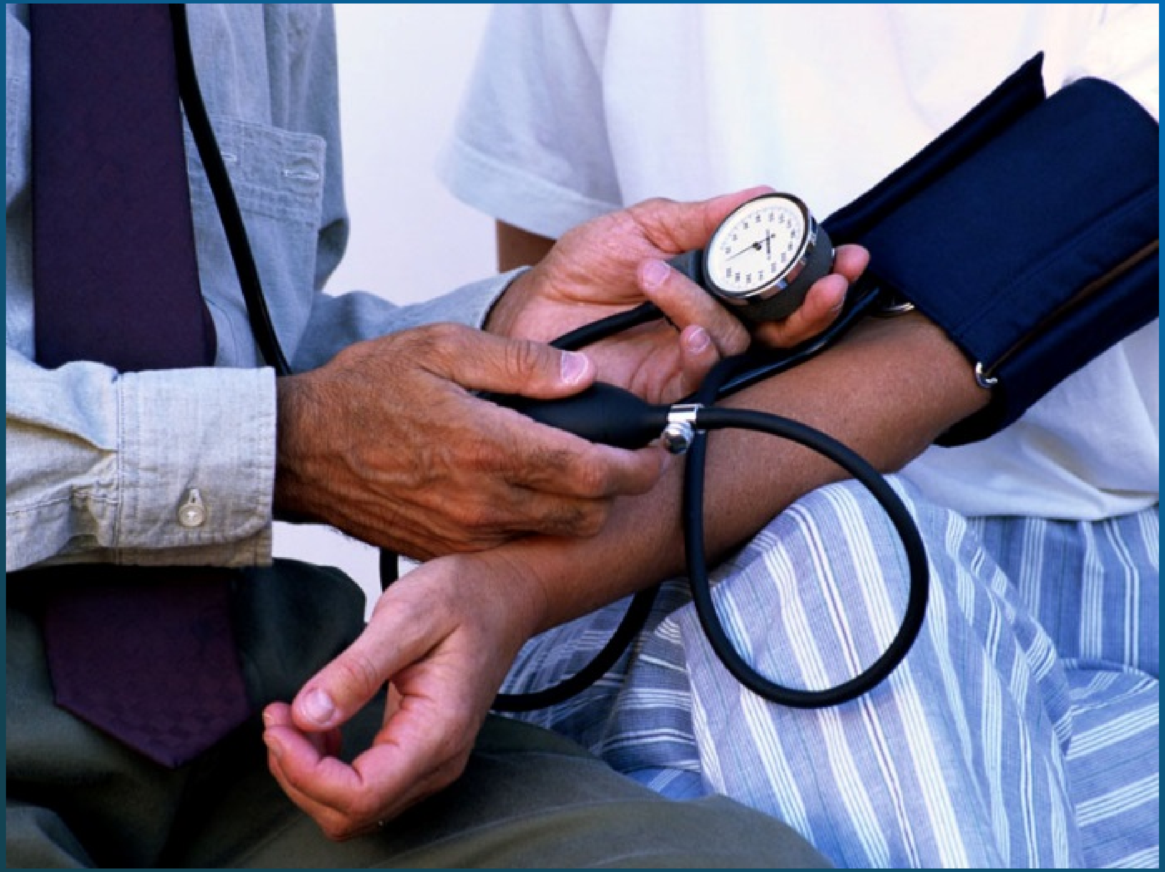

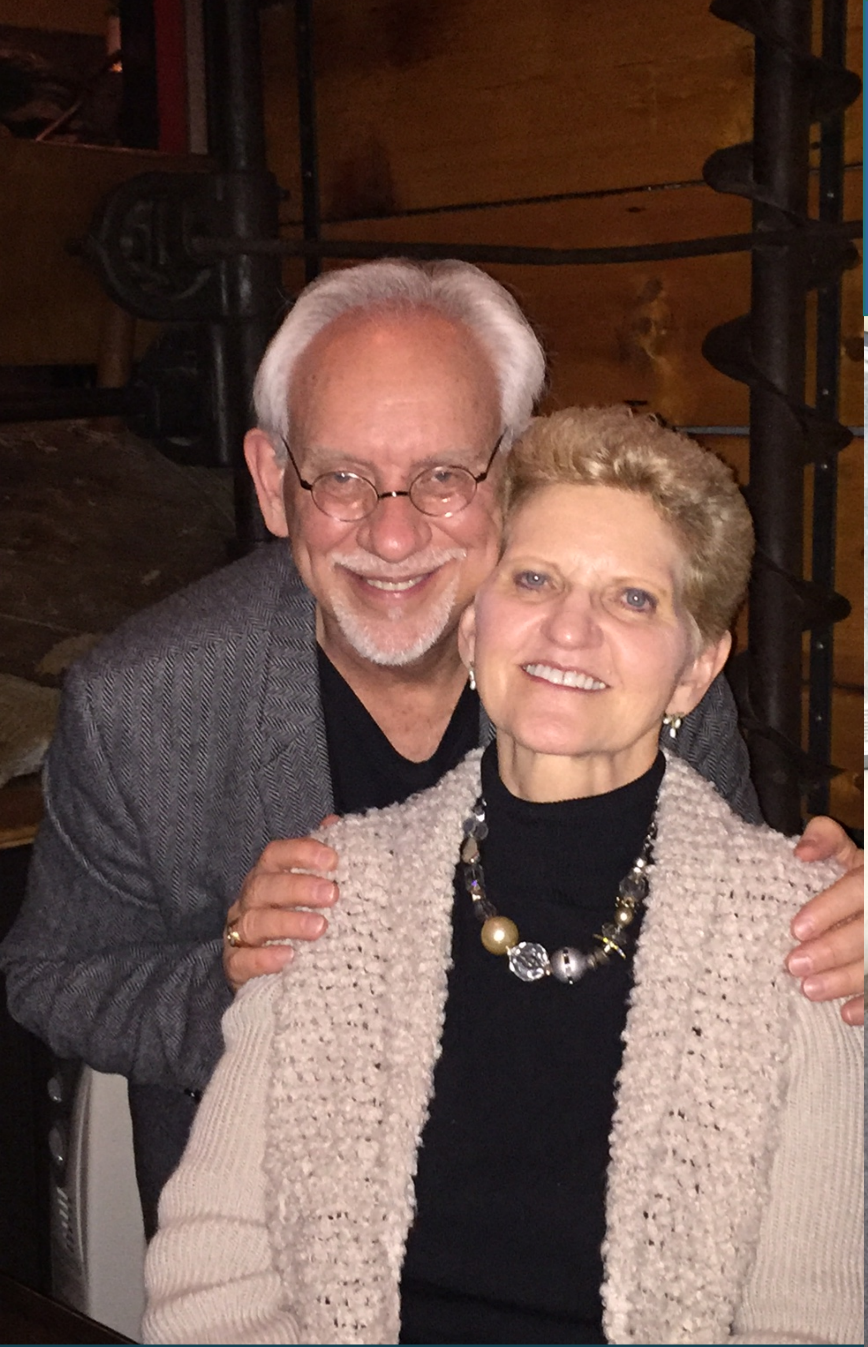

↑ Oxytocin

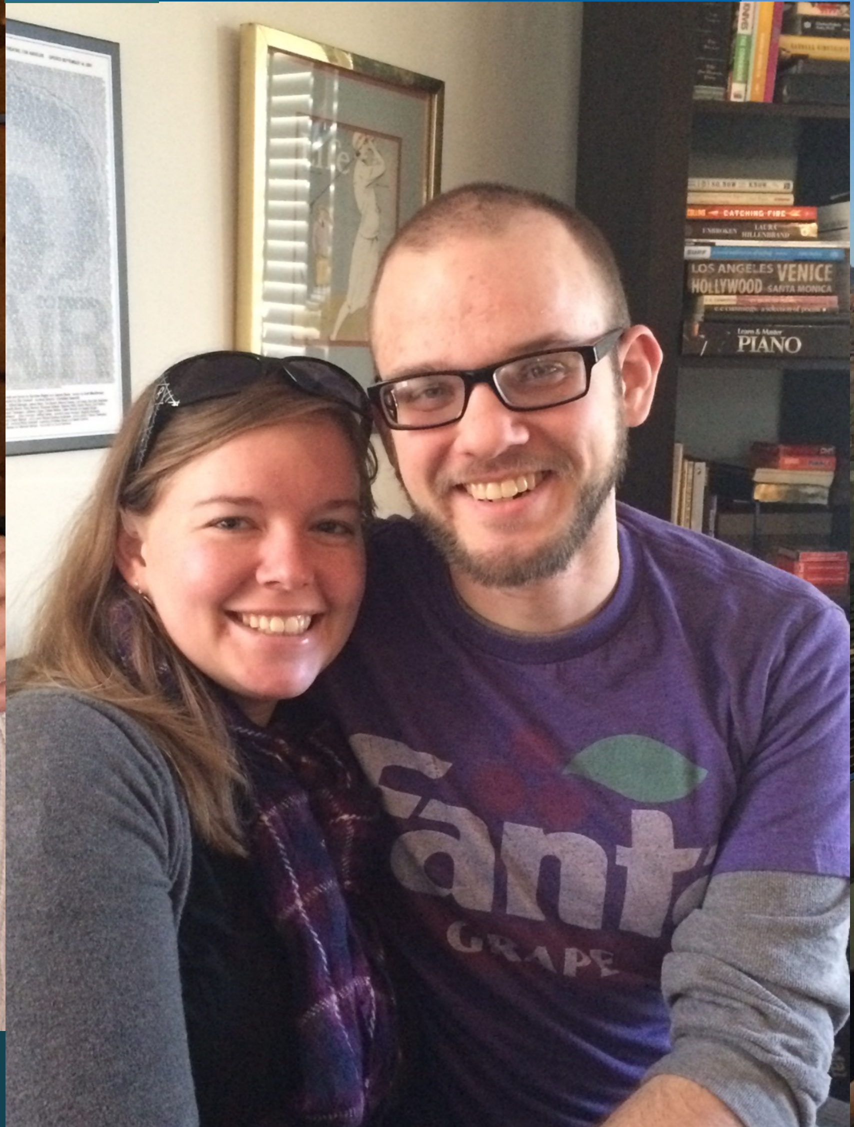

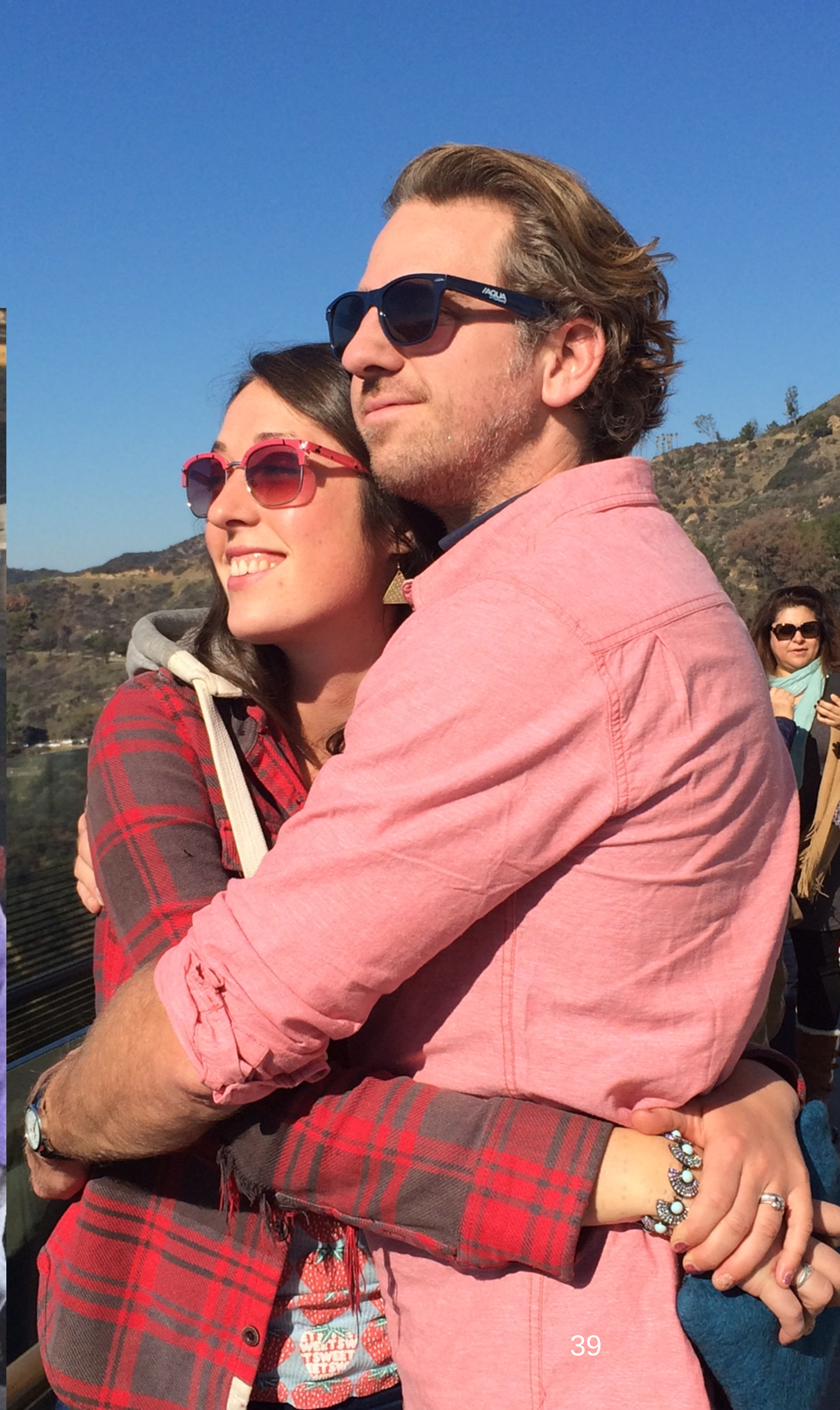

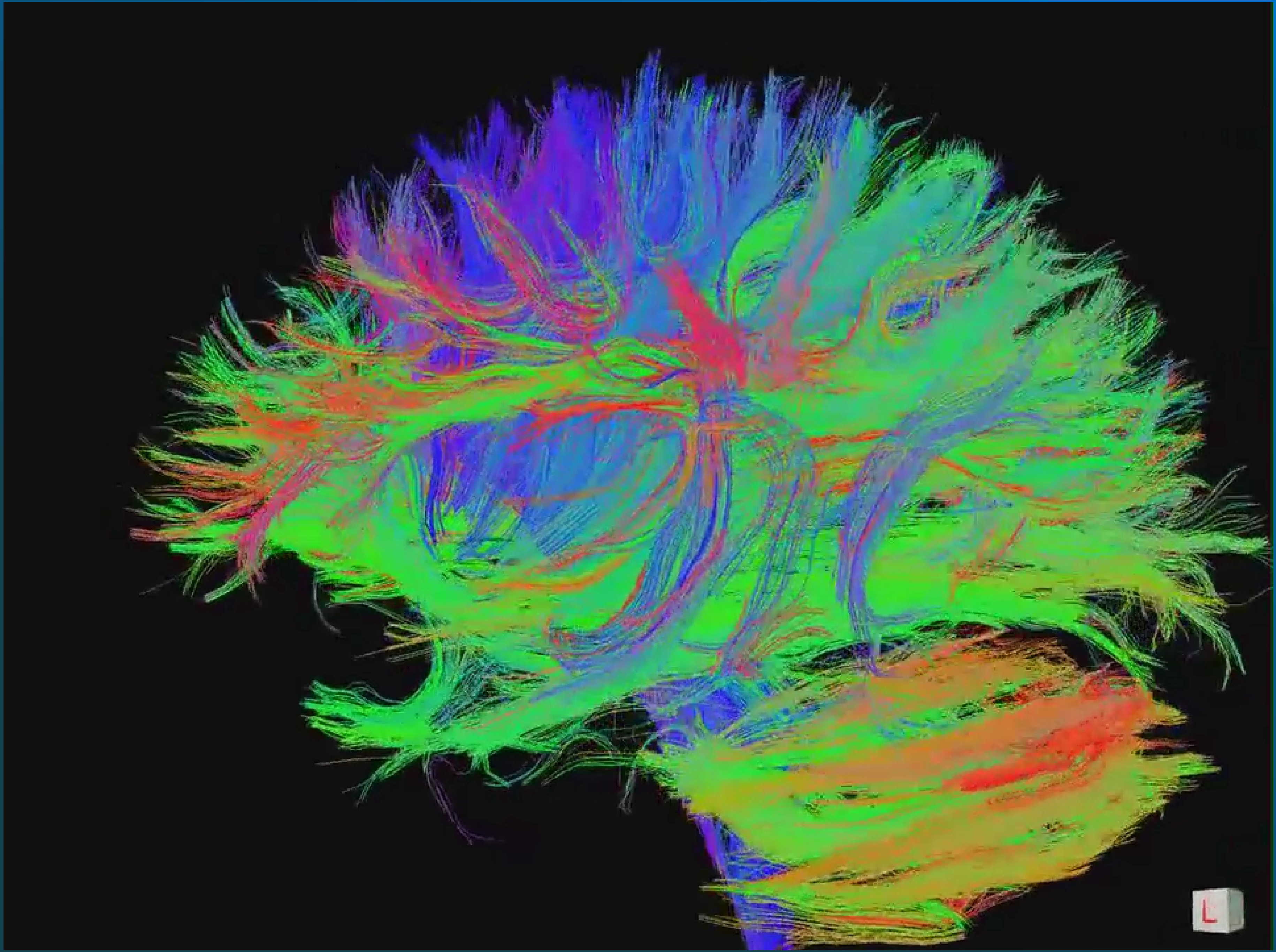

By jgmarcelino from Newcastle upon Tyne, UK (Webs'r'us Uploaded by CFCF) [CC BY 2.0 (http://creativecommons.org/licenses/by/2.0)], via Wikimedia Commons

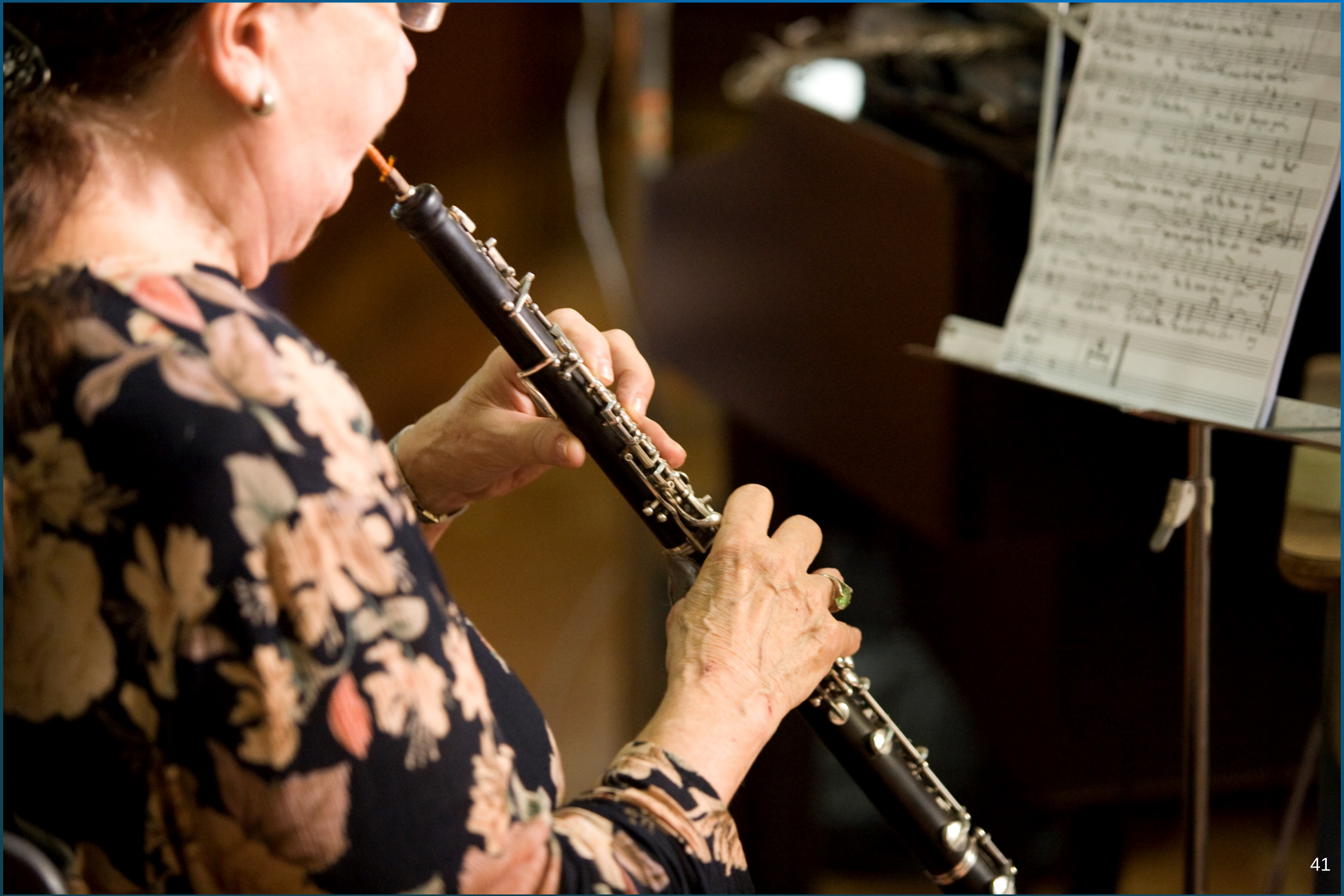

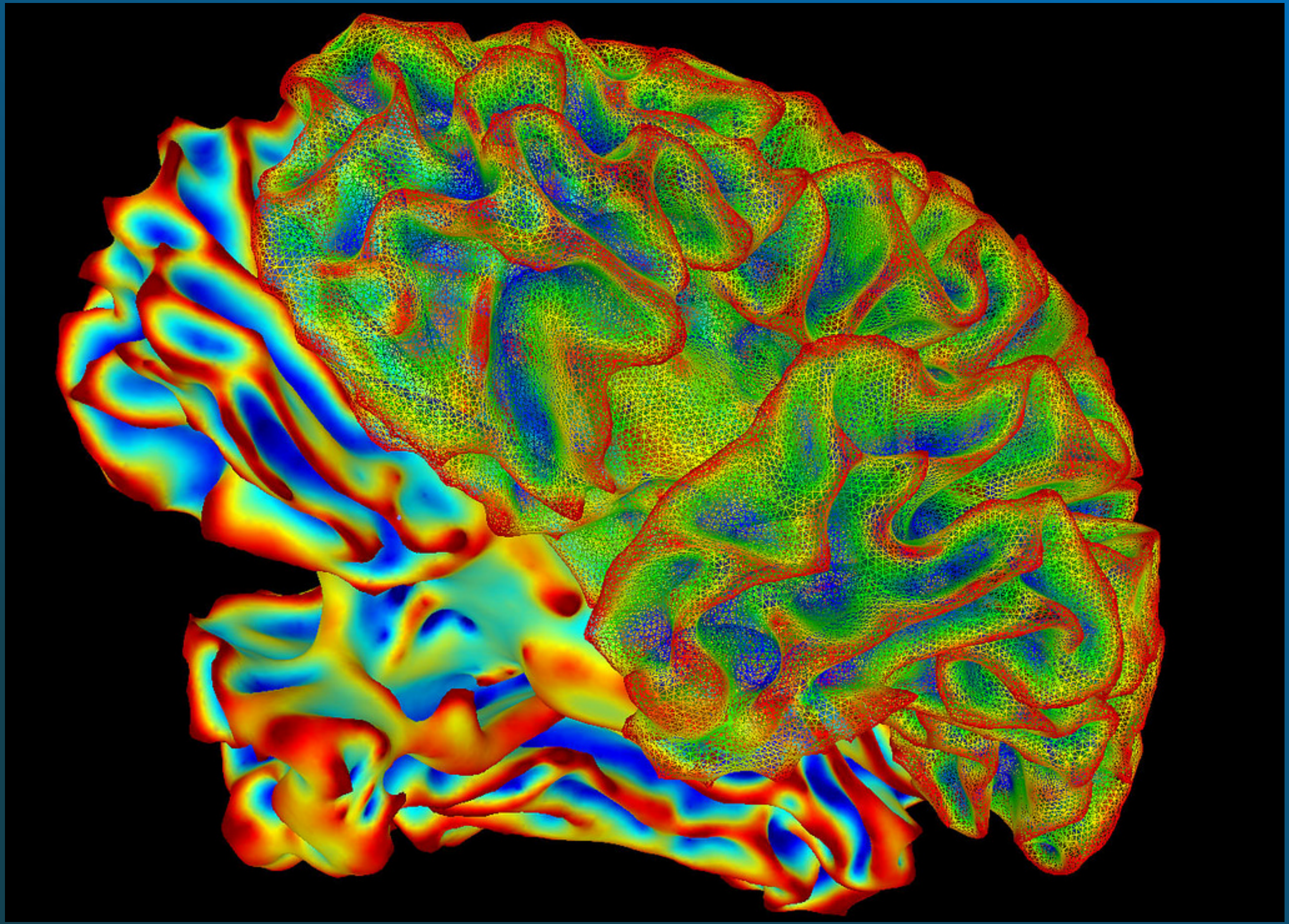

Credit: National Institute of Mental Health, National Institutes of Health

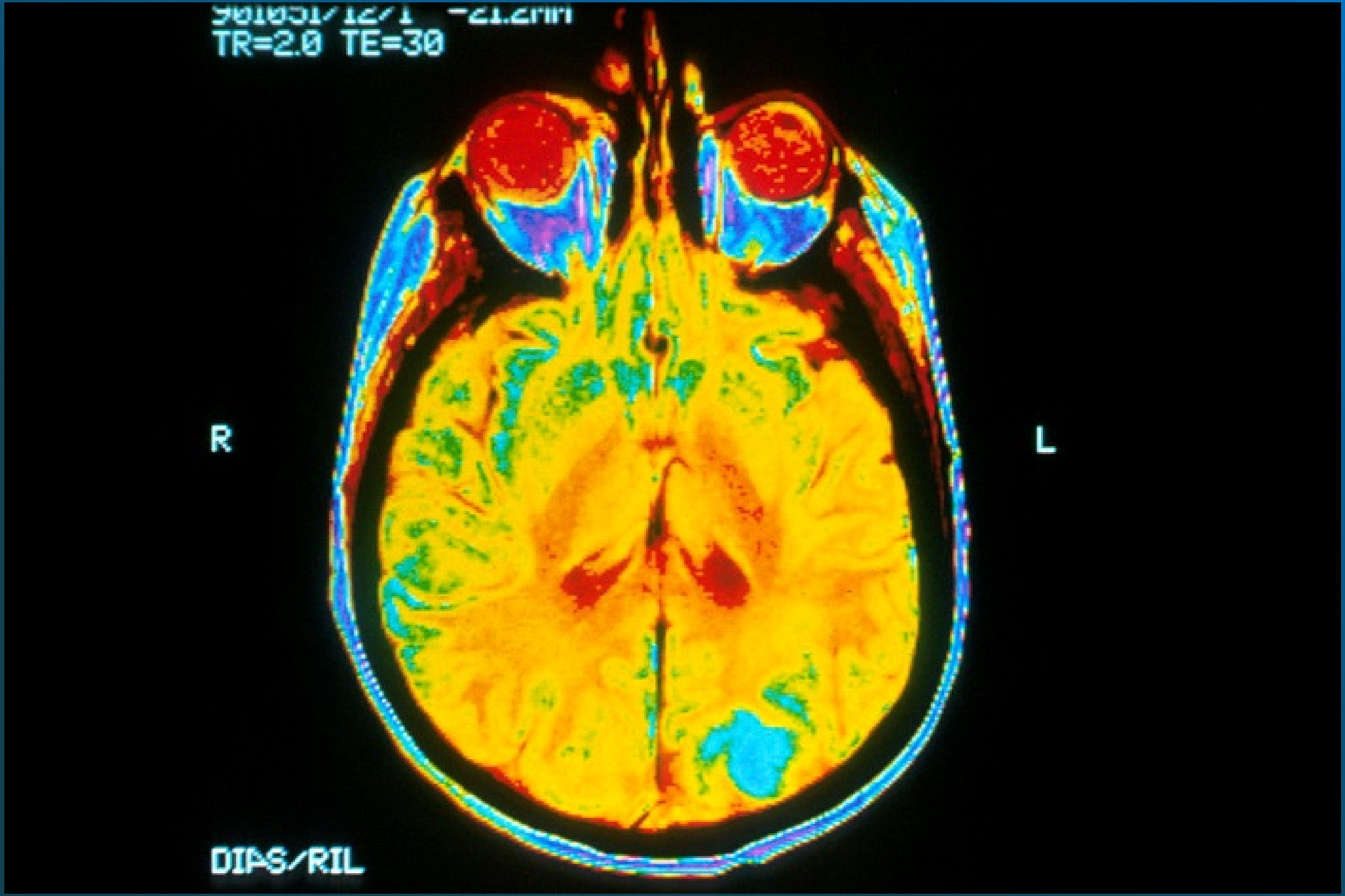
TR=2.0 TE=30
R
L
DIPS/RIL

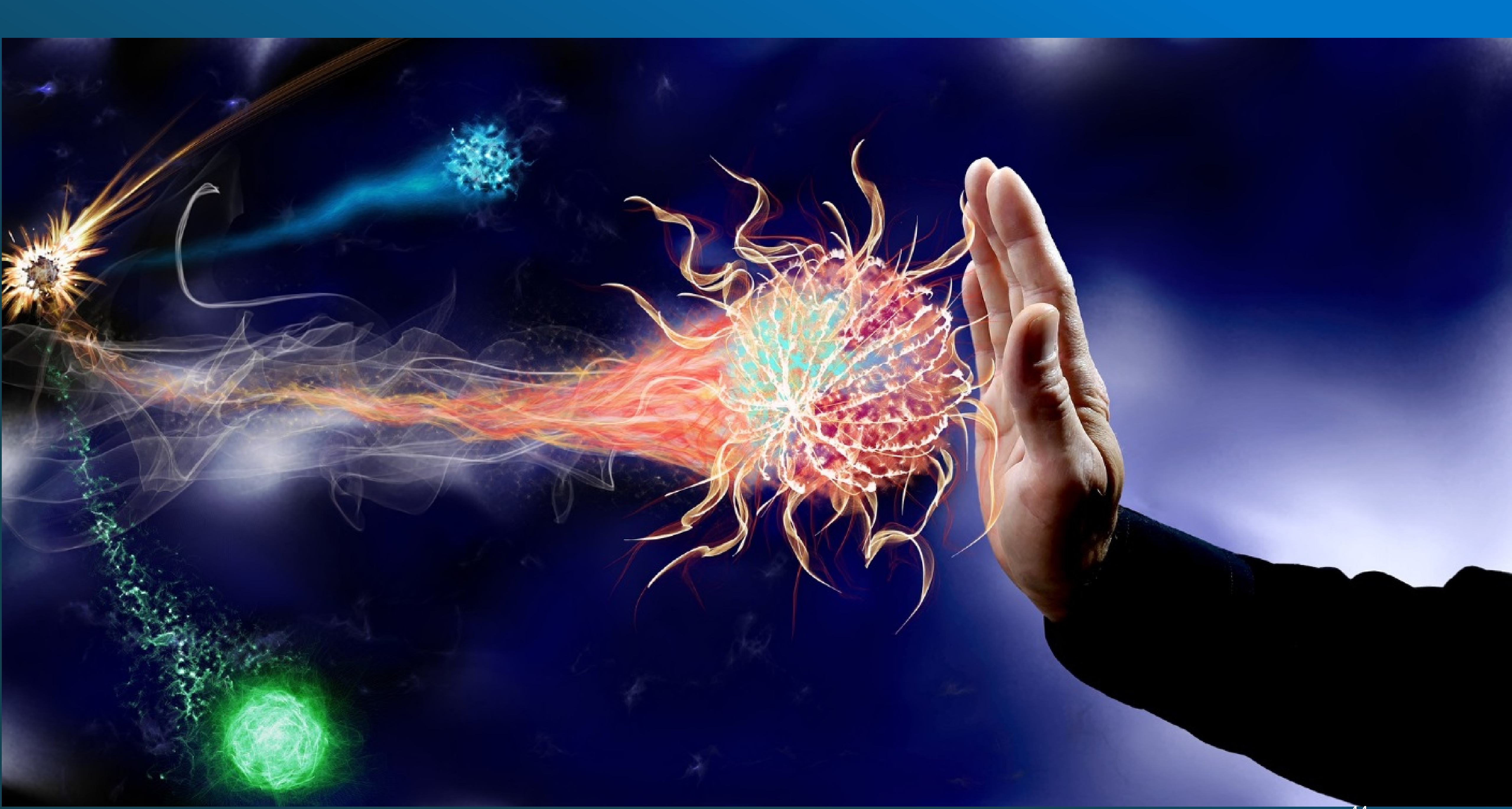

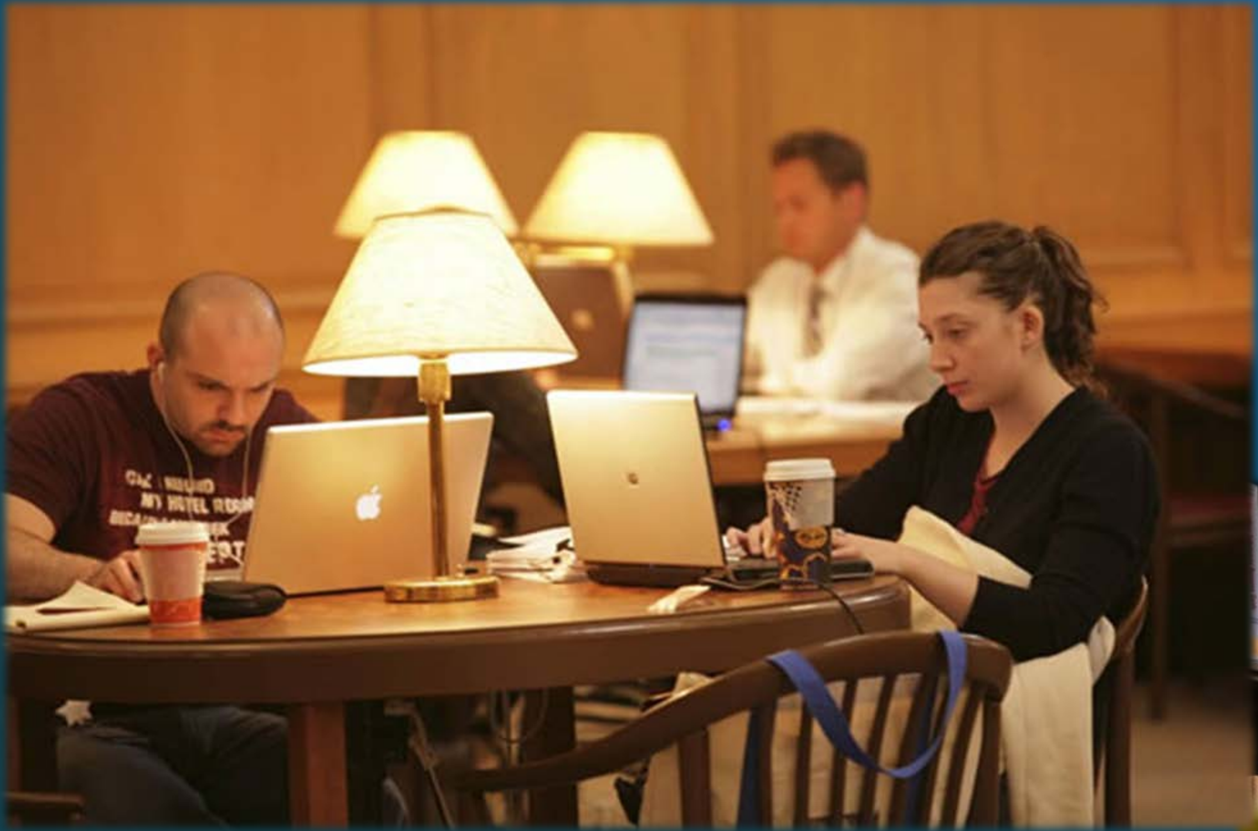

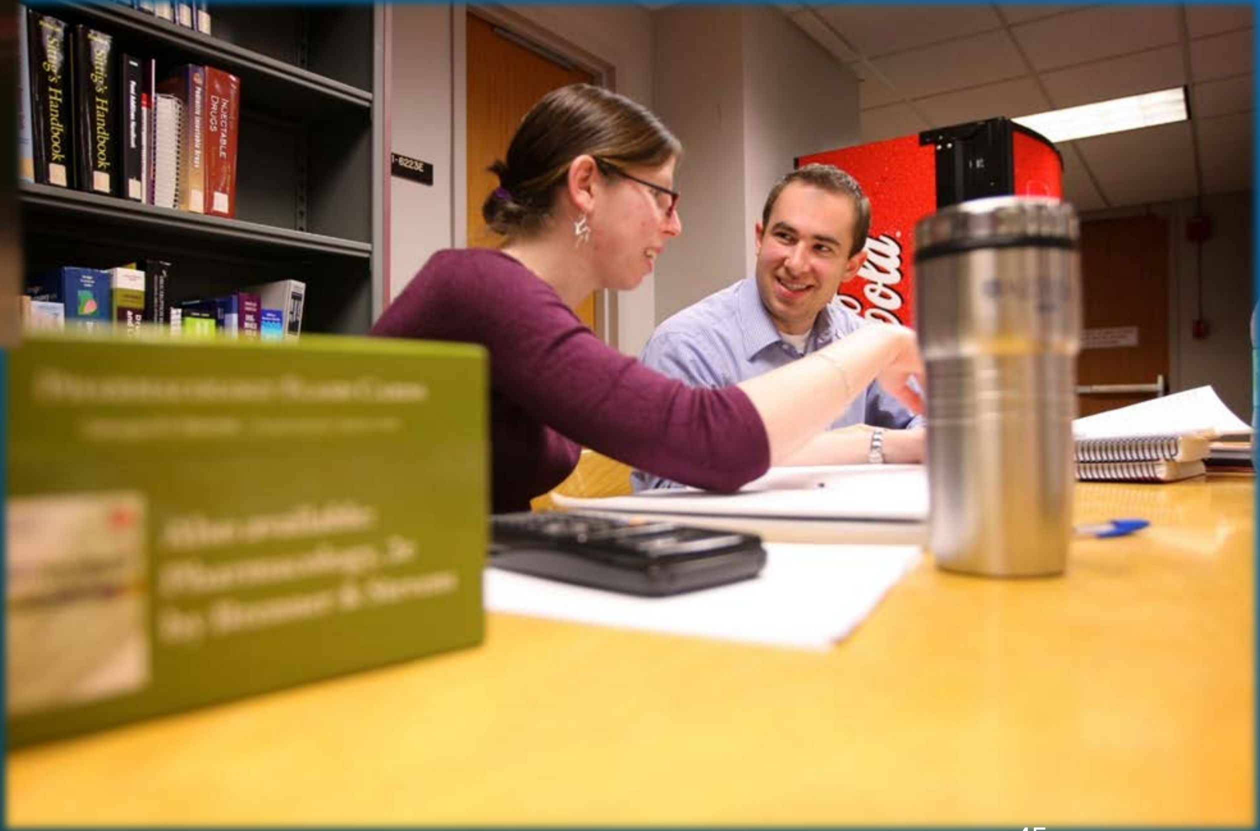
Sittig's Handbook
Sittig's Handbook
INJECTABLE DRUGS

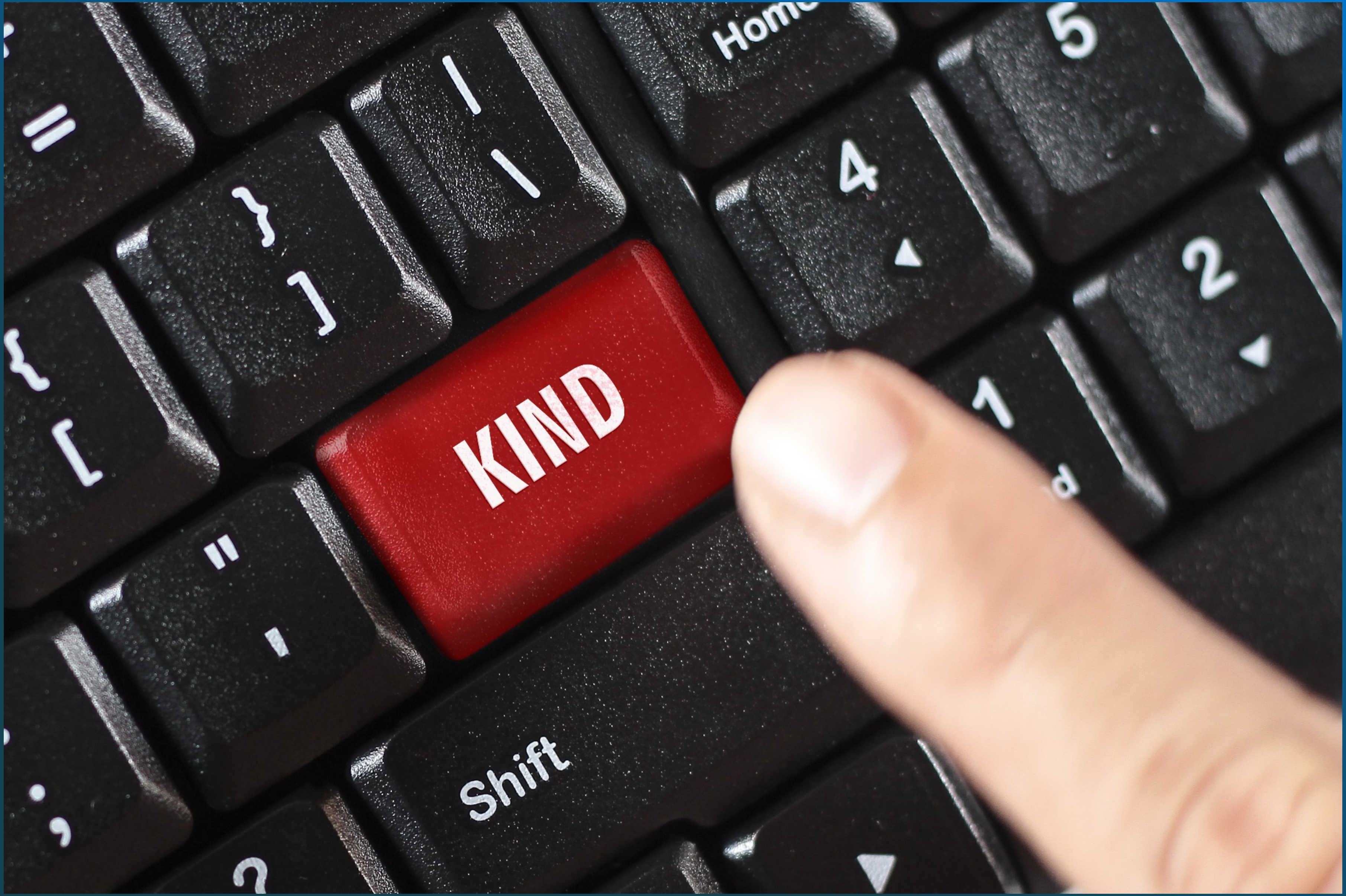
KIND
Shift

Center for Creative Leadership®

Institute *for* Mindful Leadership

MIND & LIFE INSTITUTE

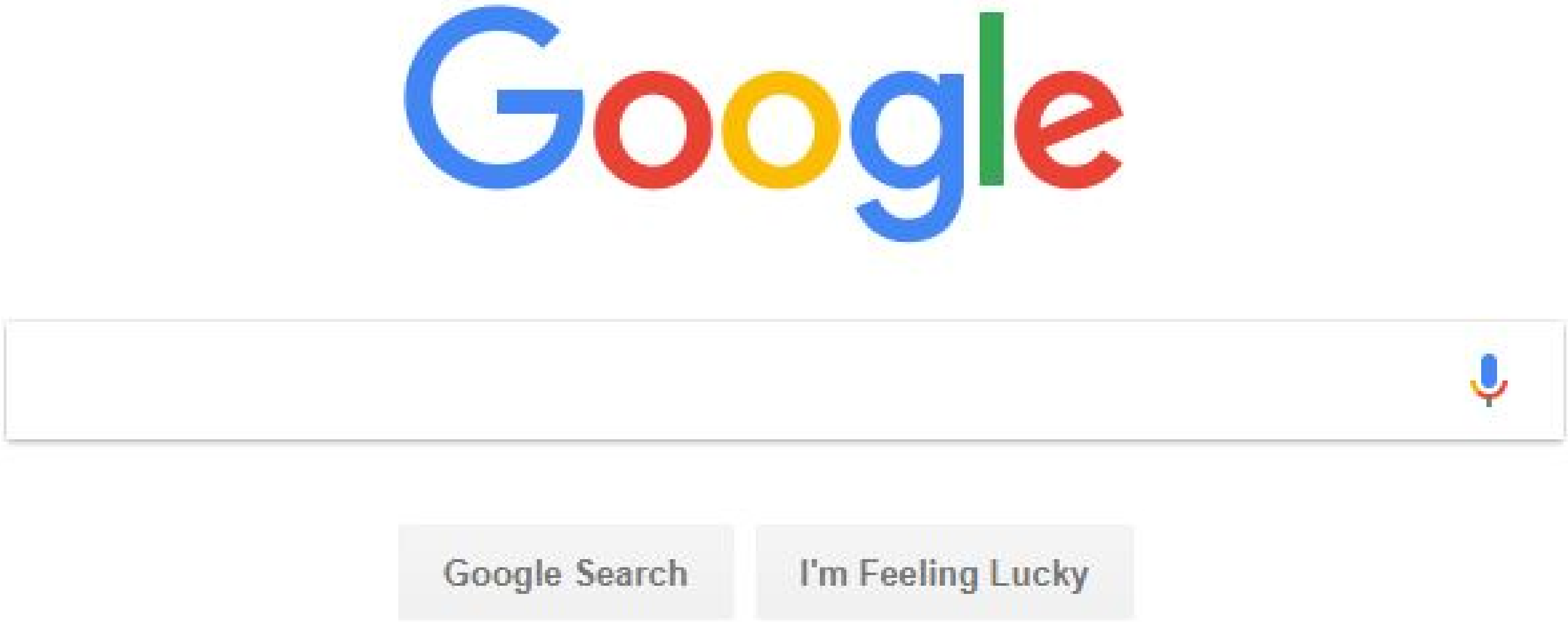
Google
Google Search
I'm Feeling Lucky

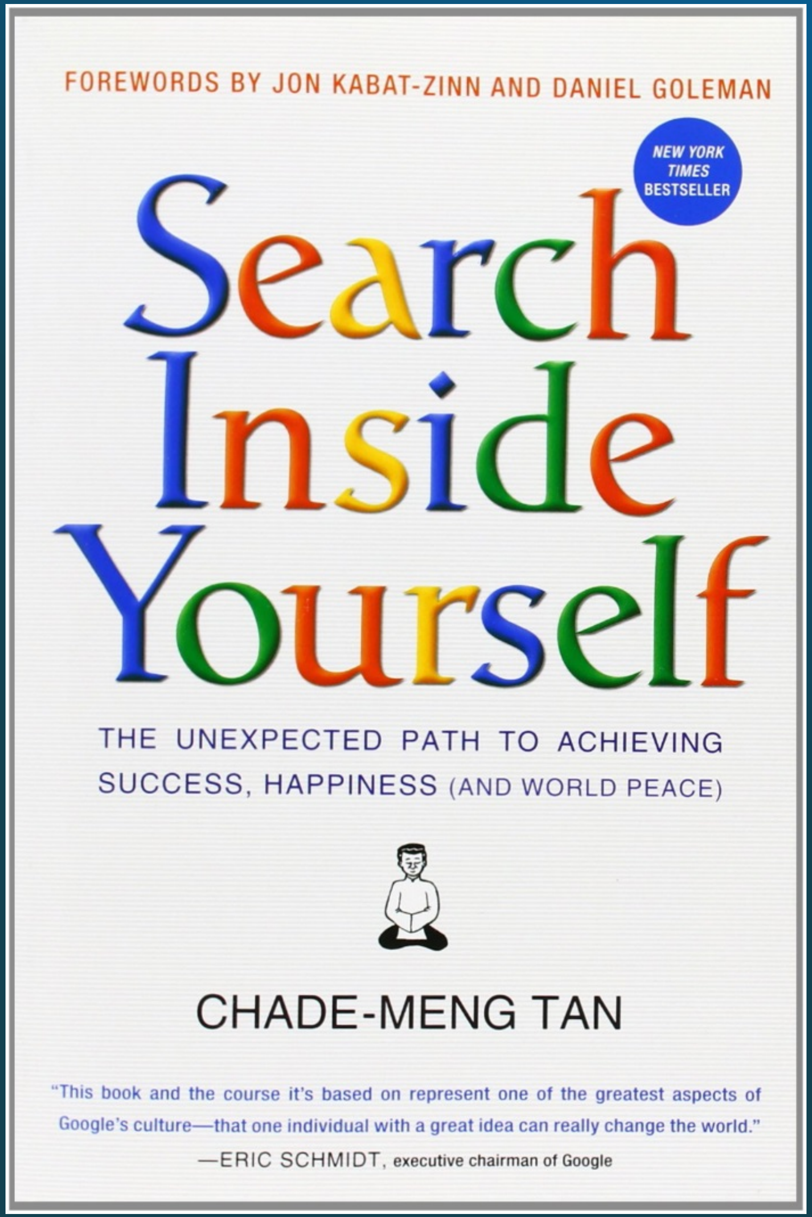
FOREWORDS BY JON KABAT-ZINN AND DANIEL GOLEMAN
NEW YORK TIMES BESTSELLER
Search Inside Yourself
THE UNEXPECTED PATH TO ACHIEVING SUCCESS, HAPPINESS (AND WORLD PEACE)
CHADE-MENG TAN
"This book and the course it's based on represent one of the greatest aspects of Google's culture—that one individual with a great idea can really change the world."
—ERIC SCHMIDT, executive chairman of Google

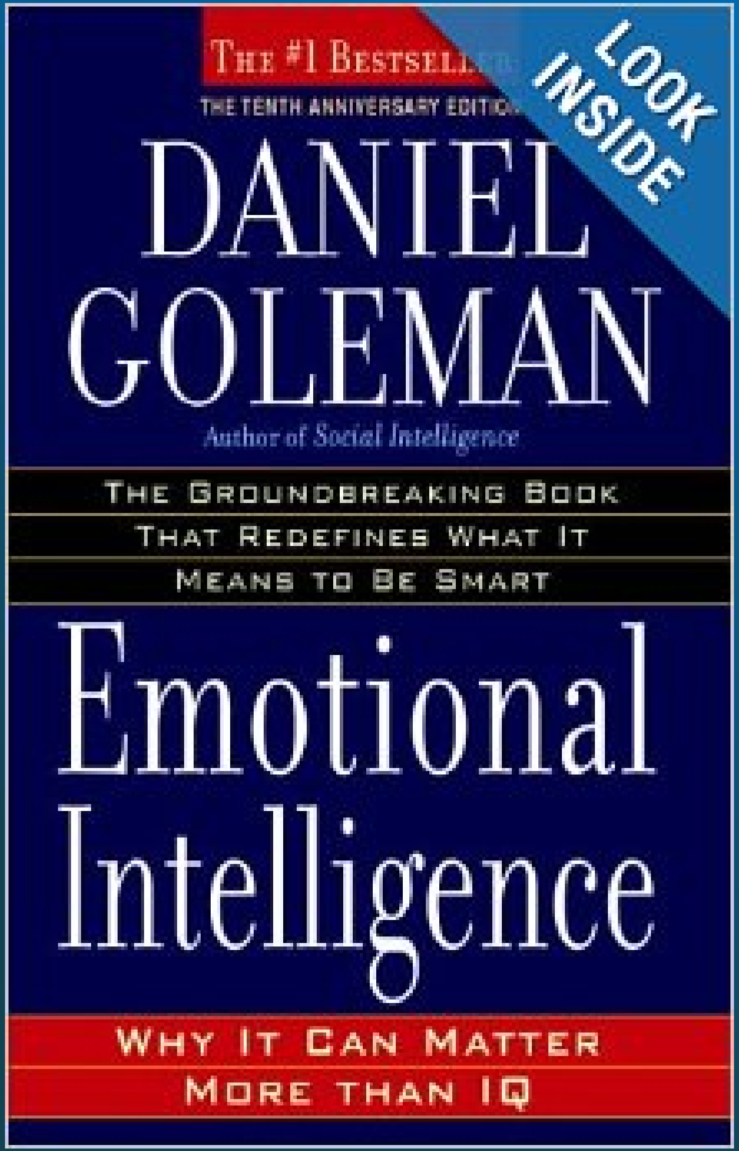
The #1 Bestsell
THE TENTH ANNIVERSARY EDITIO
LOOK INSIDE
DANIEL GOLEMAN
Author of Social Intelligence
The Groundbreaking Book That Redefines What It Means to Be Smart
Emotional Intelligence
Why It Can Matter More than IQ

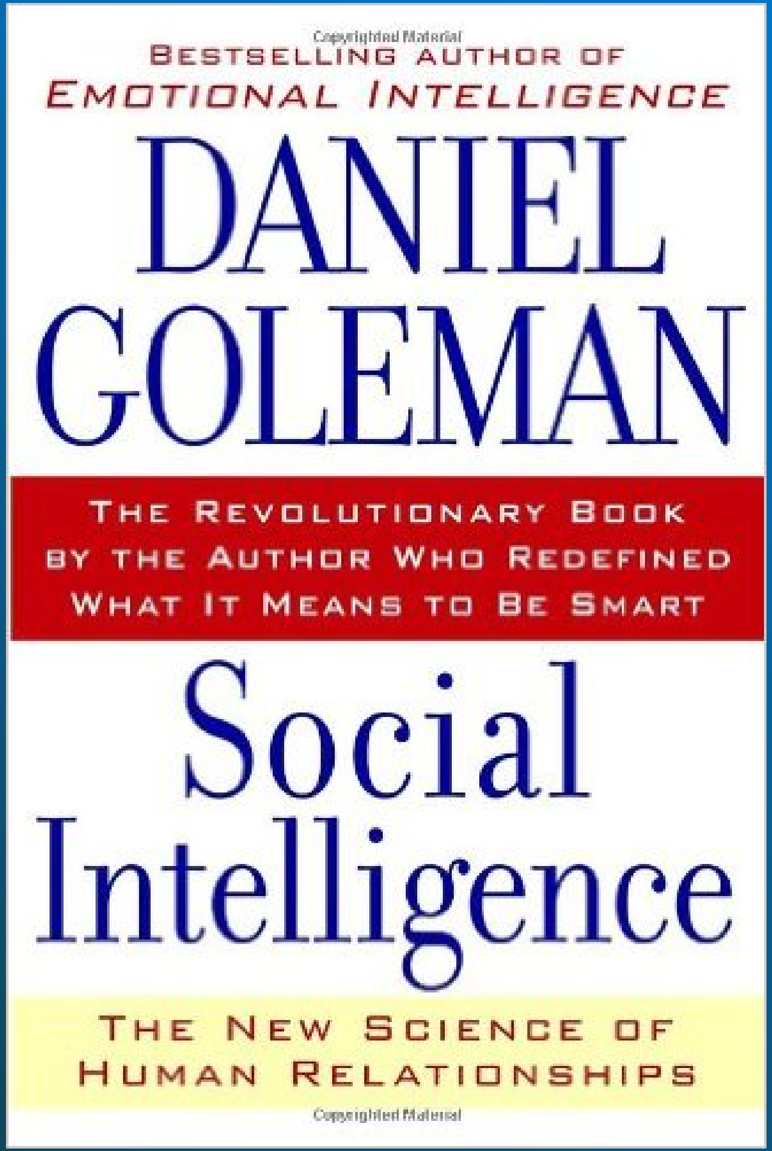
Copyrighted Material
Bestselling Author of
Emotional Intelligence
DANIEL GOLEMAN
The Revolutionary Book by the Author Who Redefined What It Means to Be Smart
Social Intelligence
The New Science of Human Relationships
Copyrighted Material

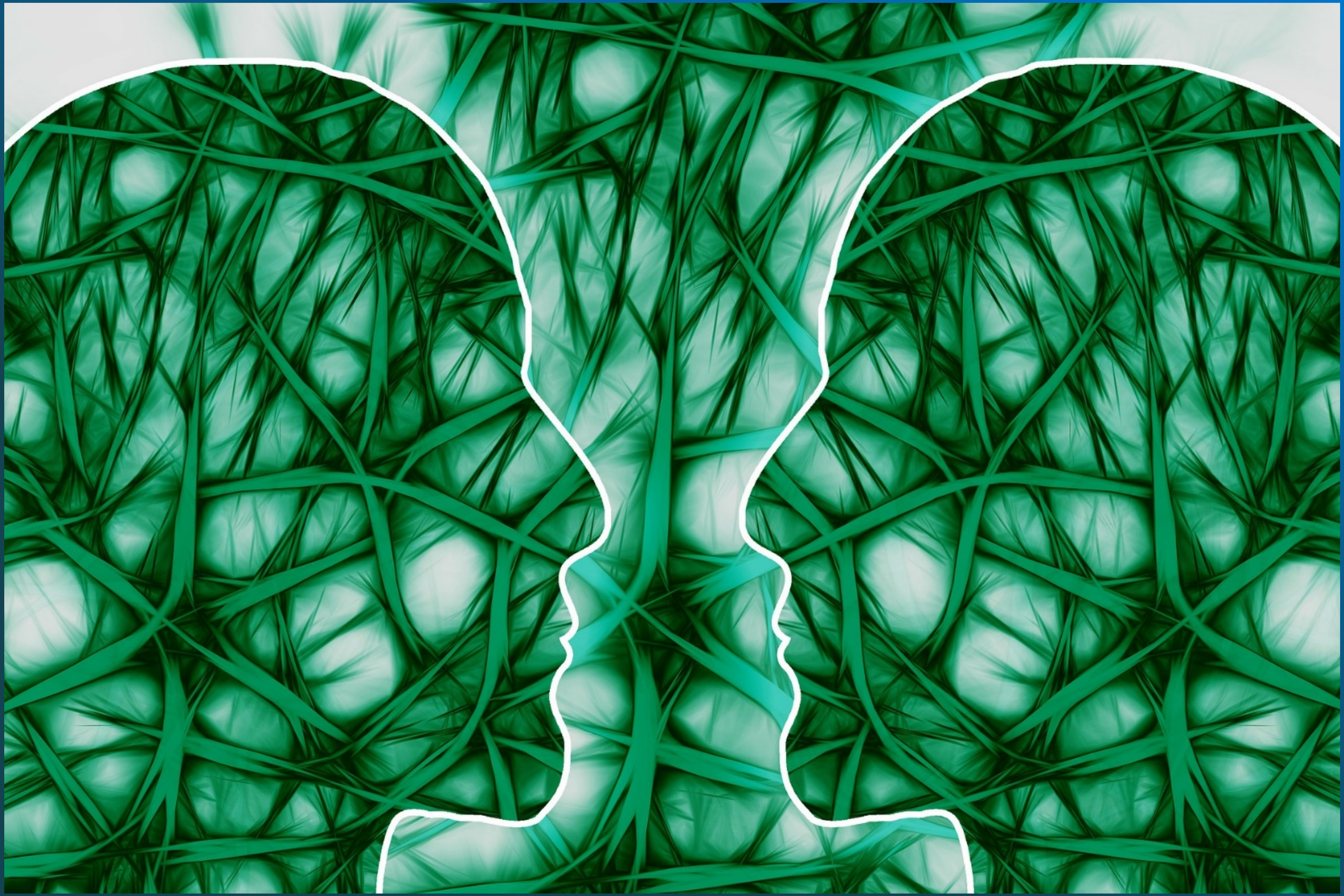

Cognitive culture

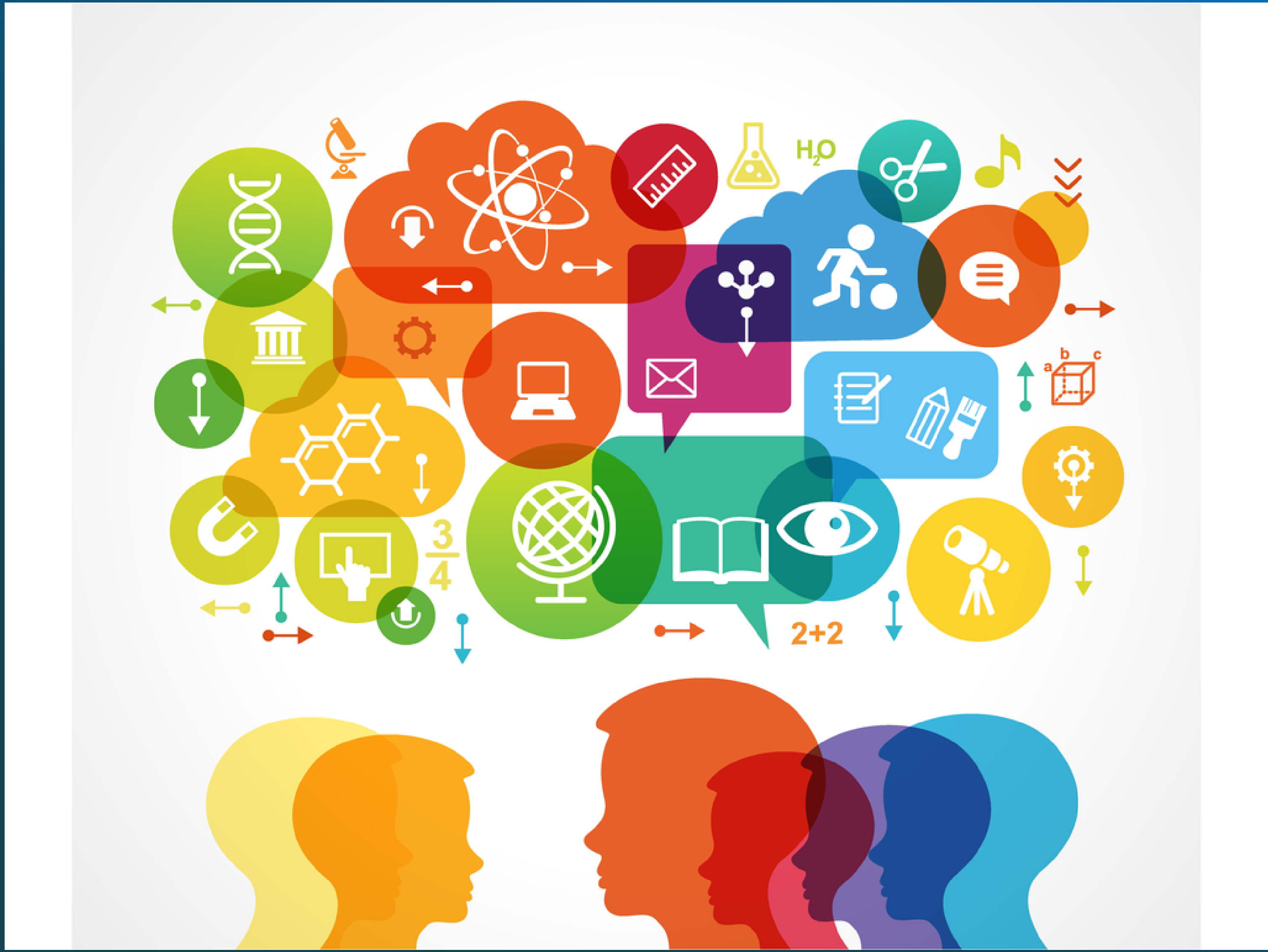

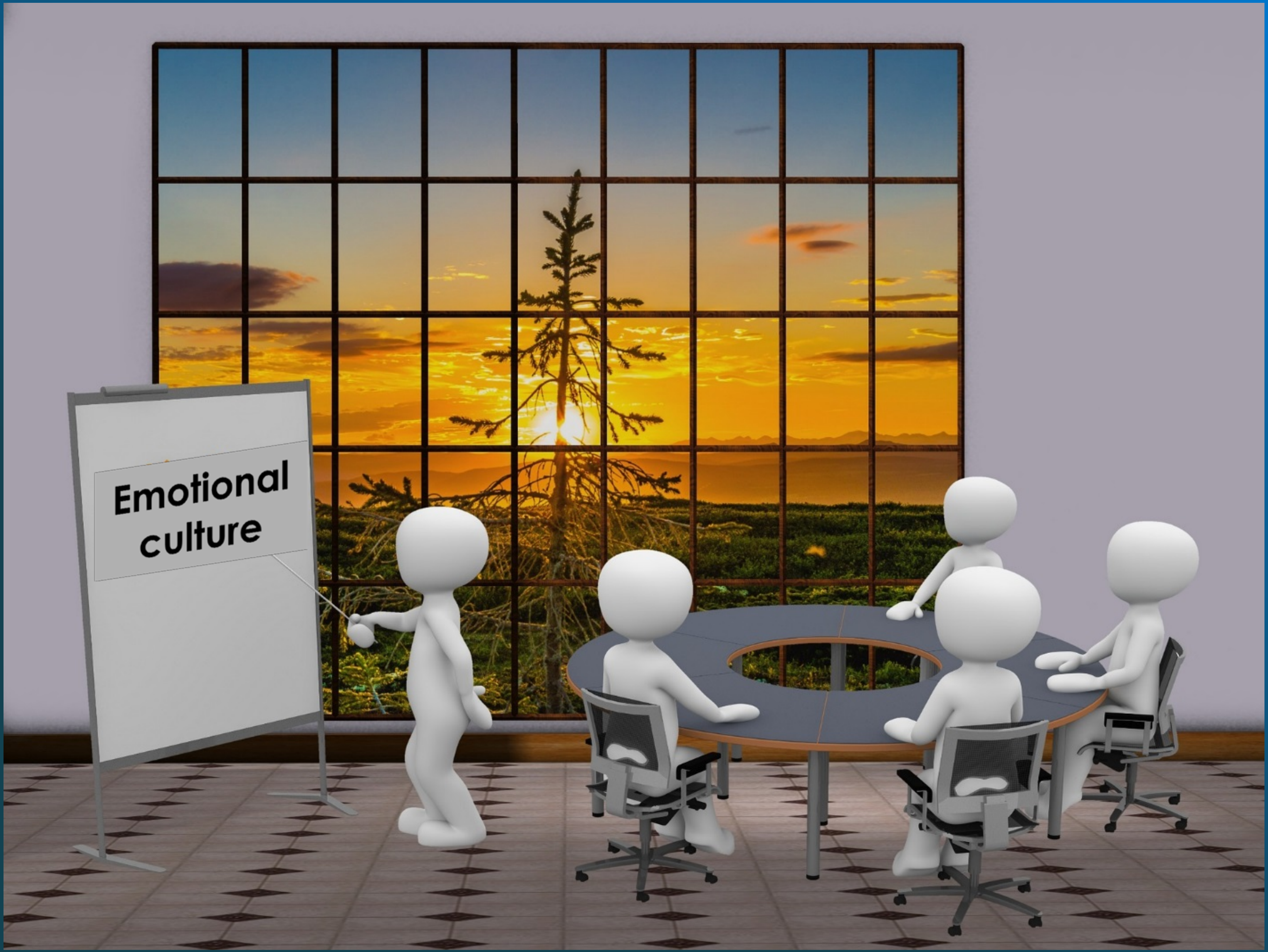
Emotional
culture

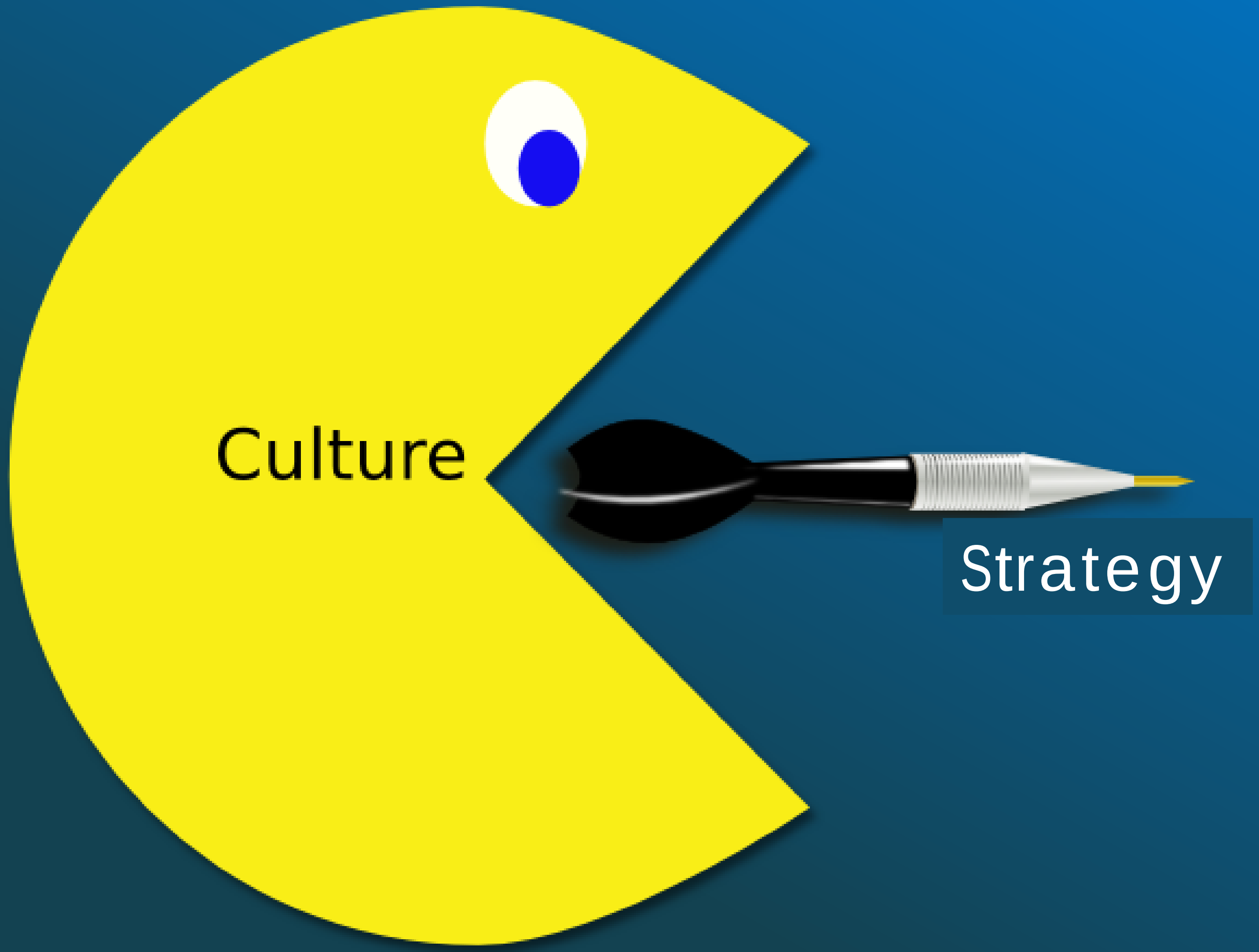
Culture
Strategy

Nobody cares how much you know until they know how much you care.

-- Theodore Roosevelt

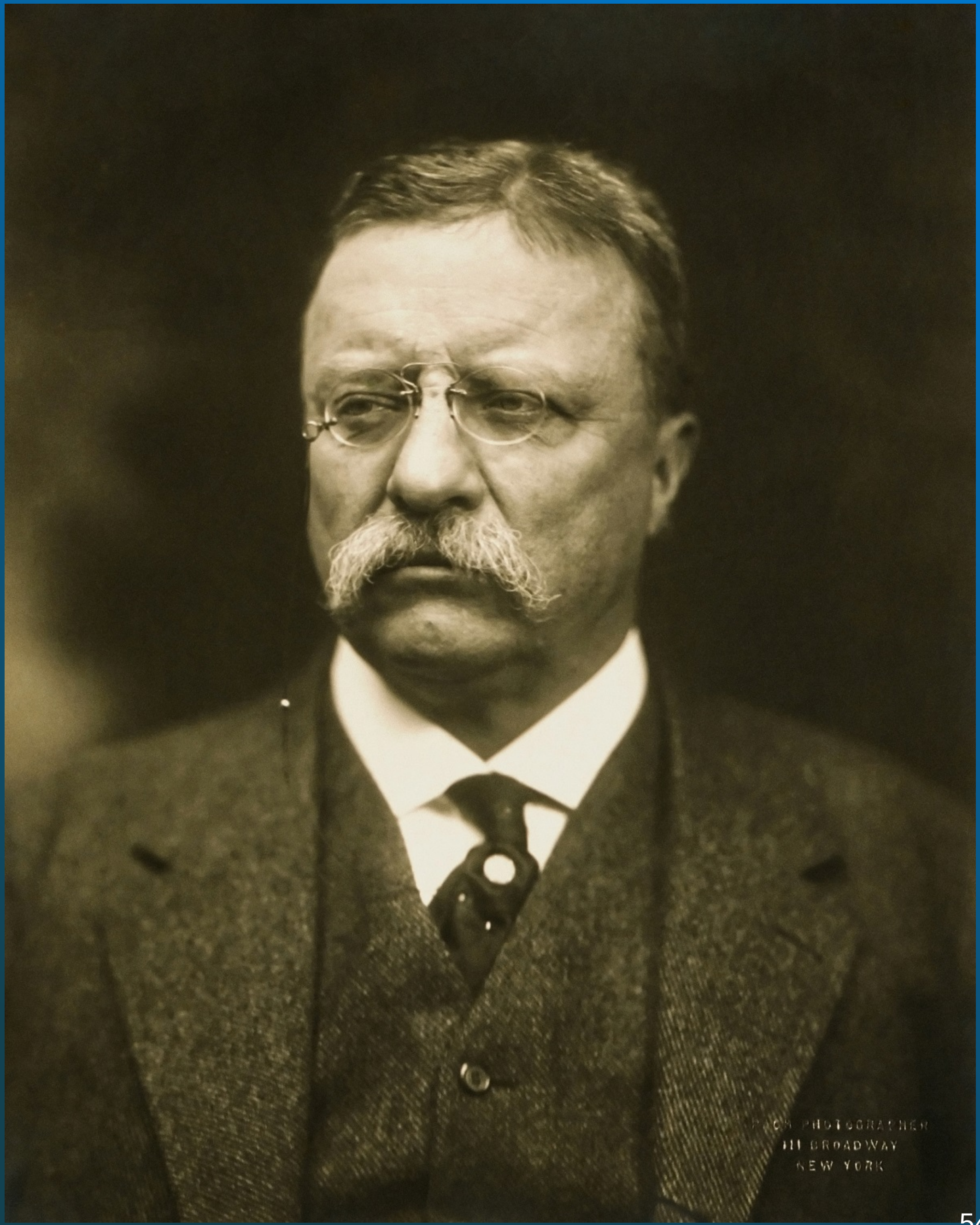

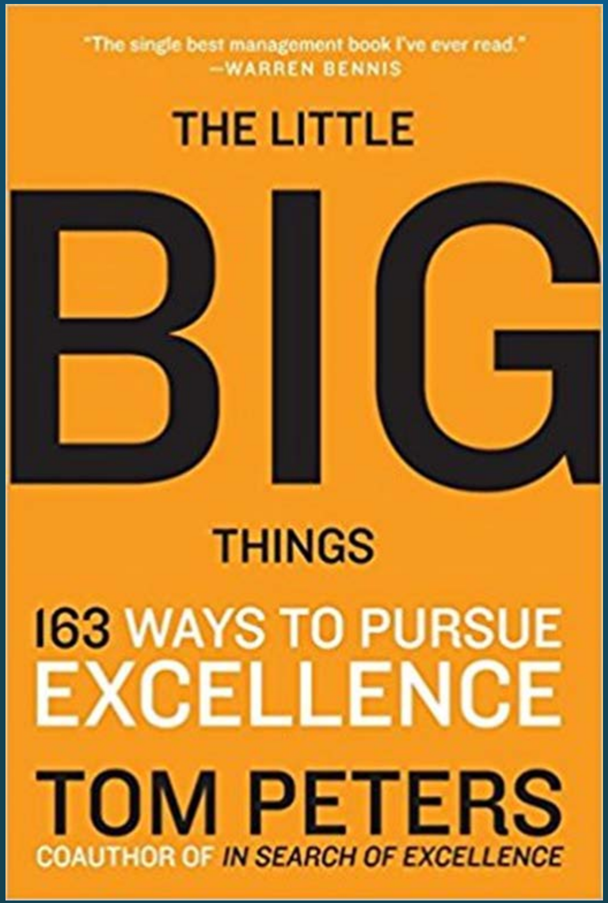

Kindness Is Free.

--Tom Peters

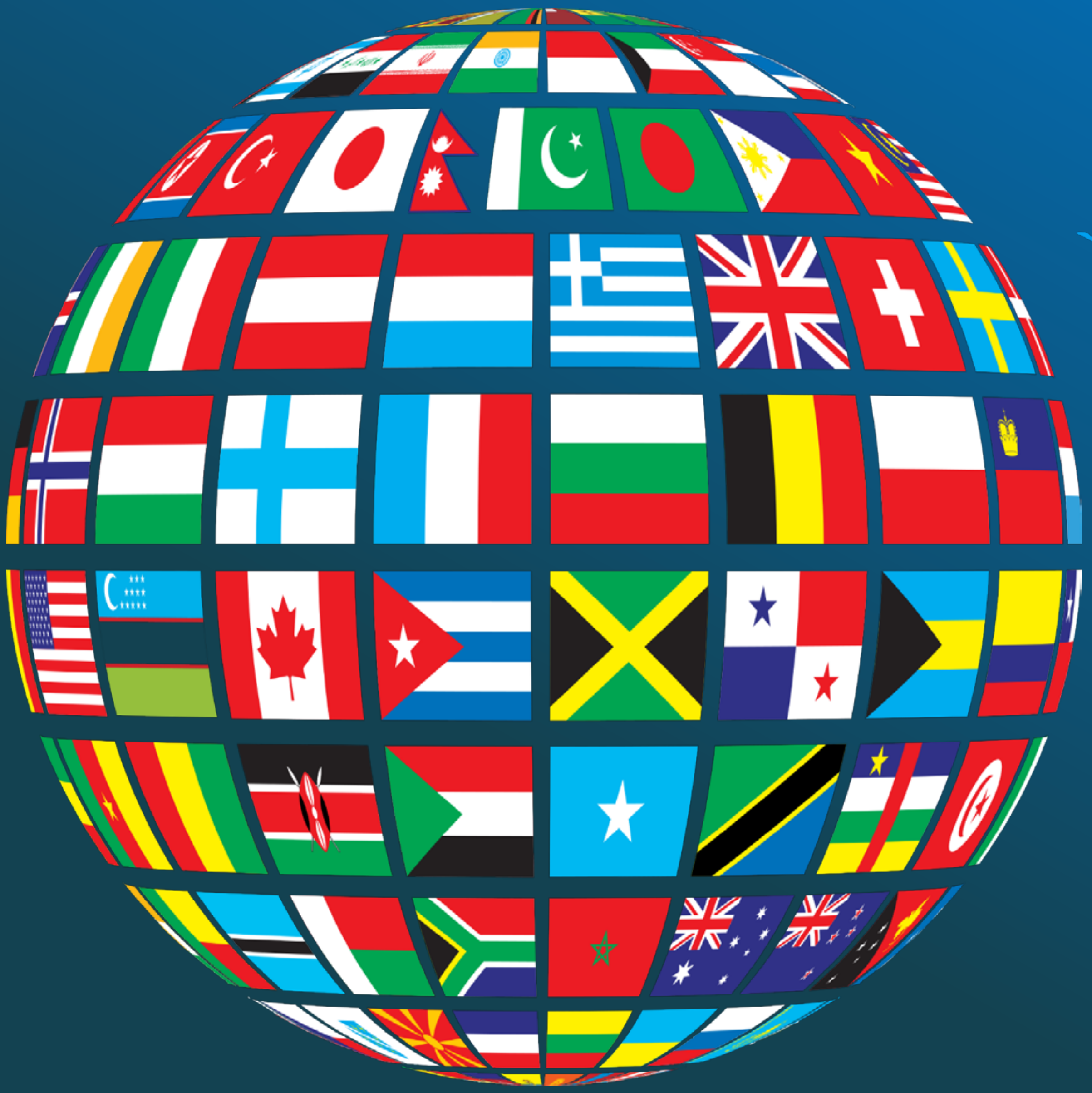

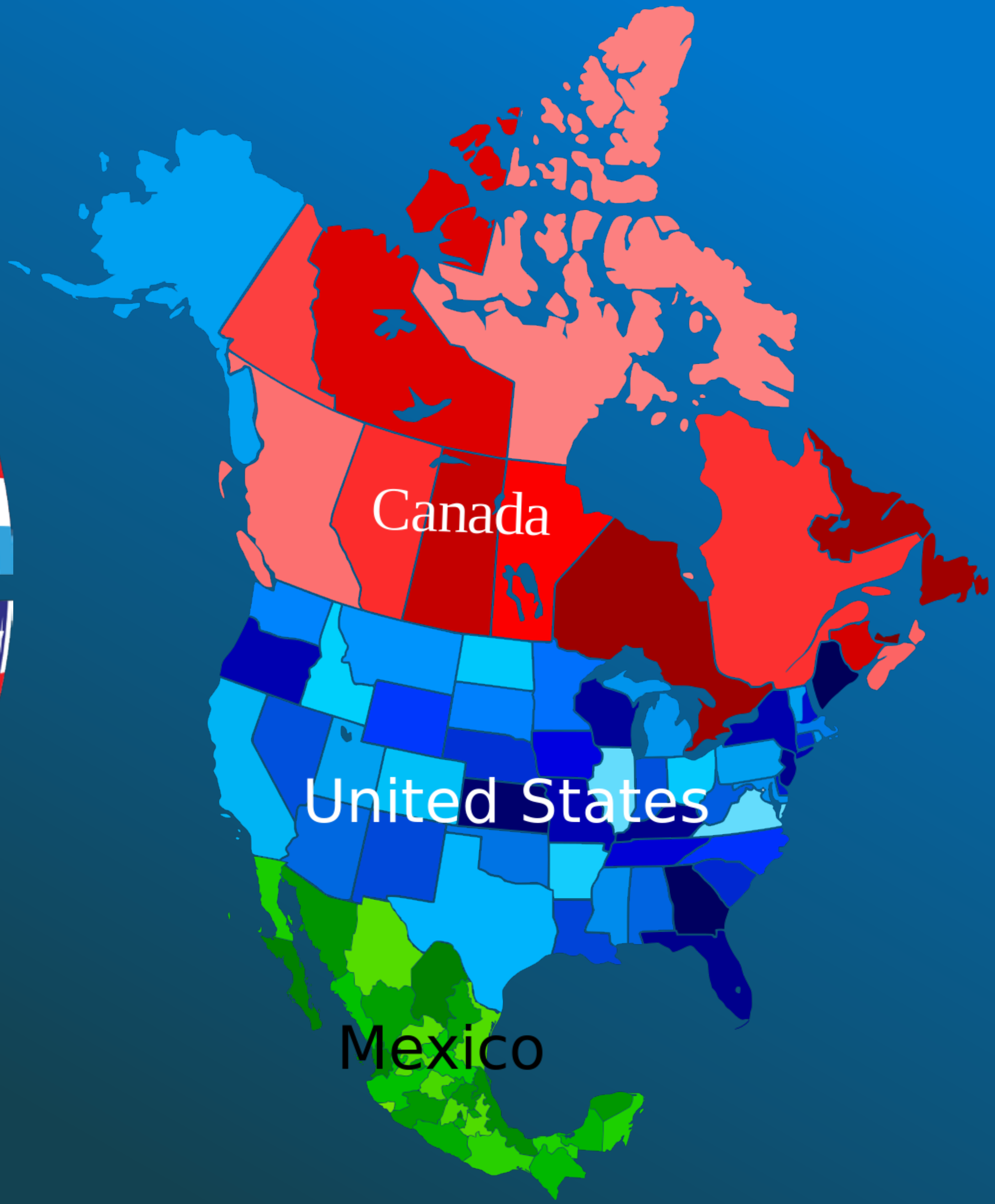
Canada
United States
Mexico

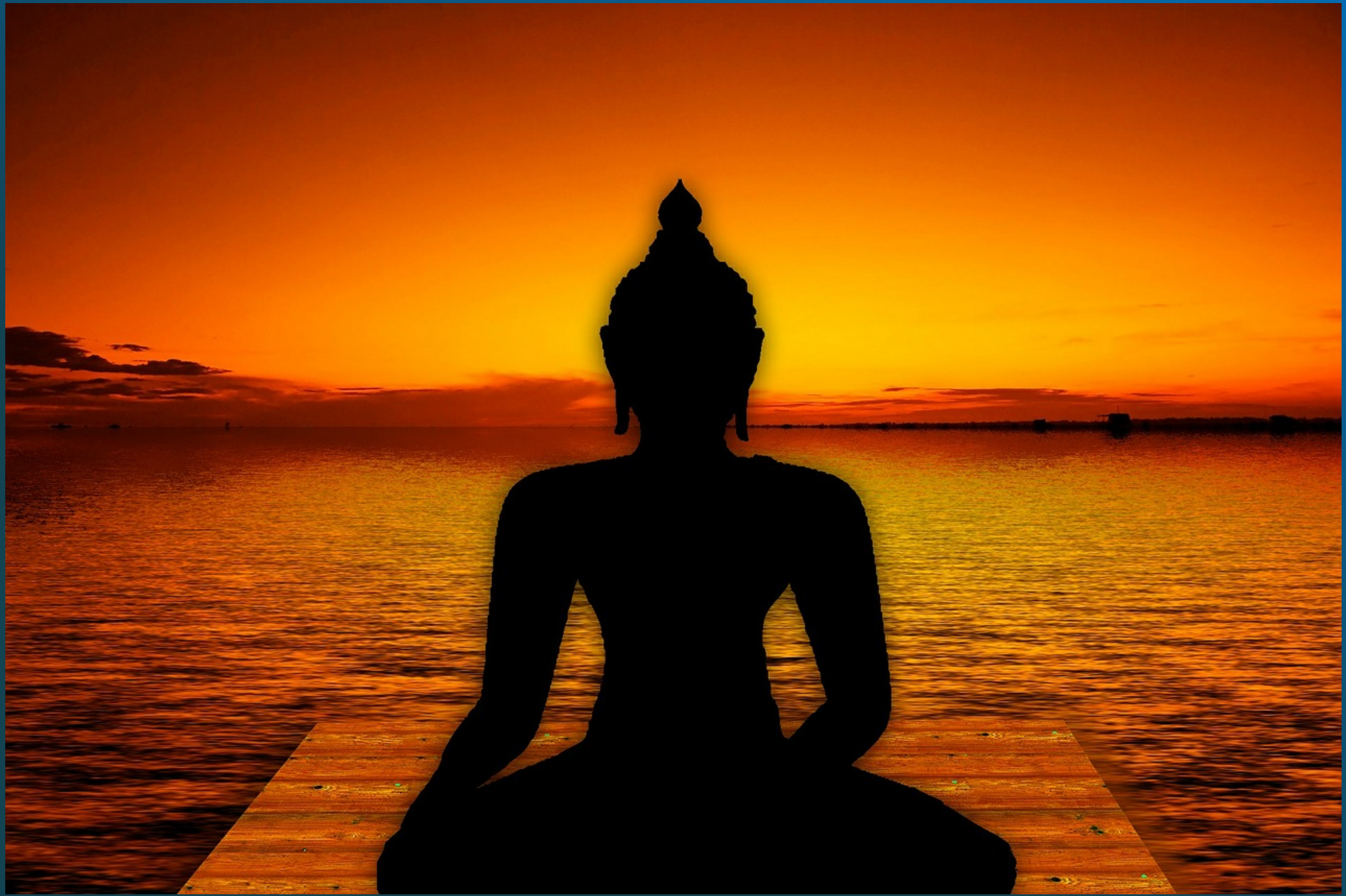

What we think, we become.

-- Buddha

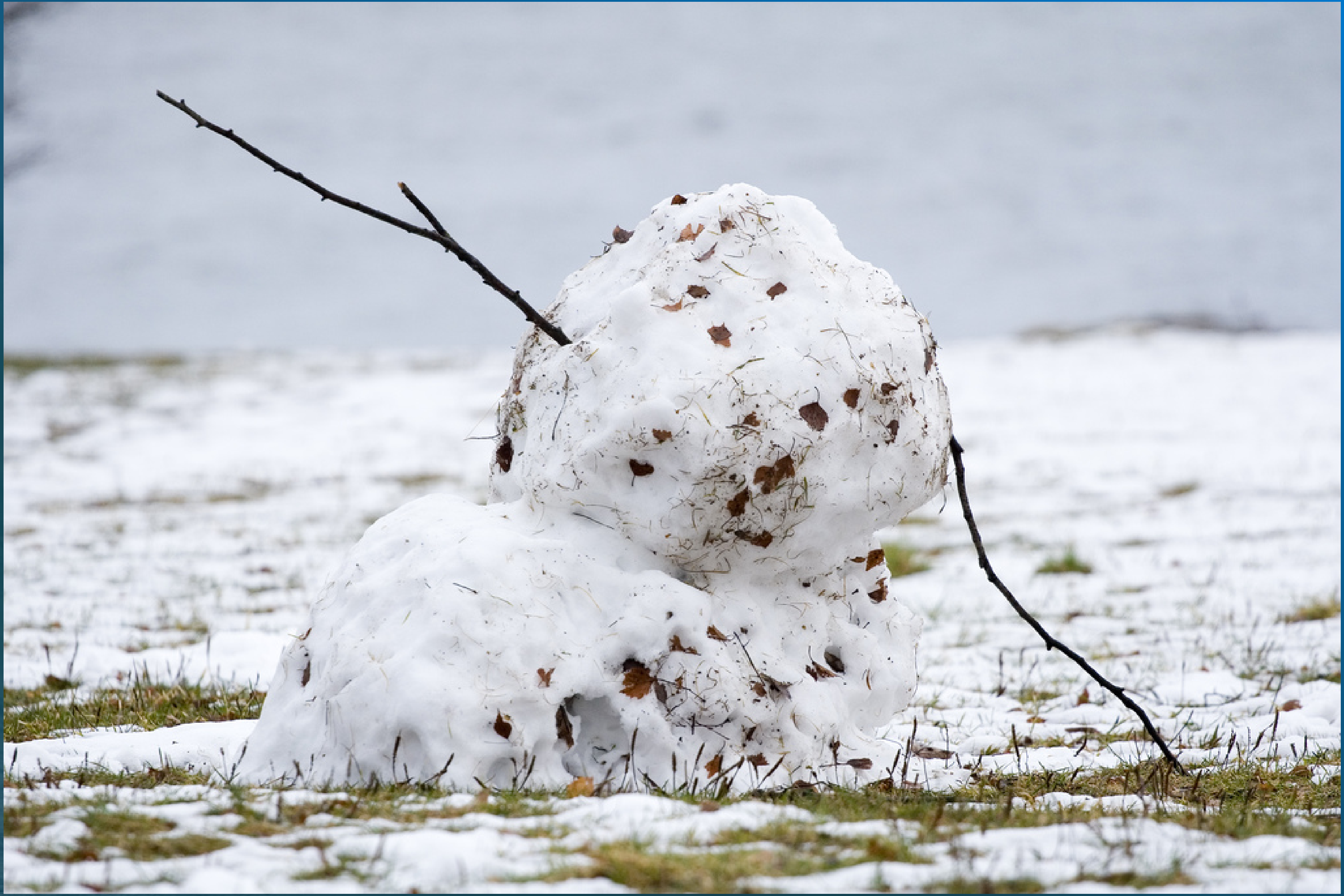

Photo by Andreas Åkre Solberg, 3/20/2007

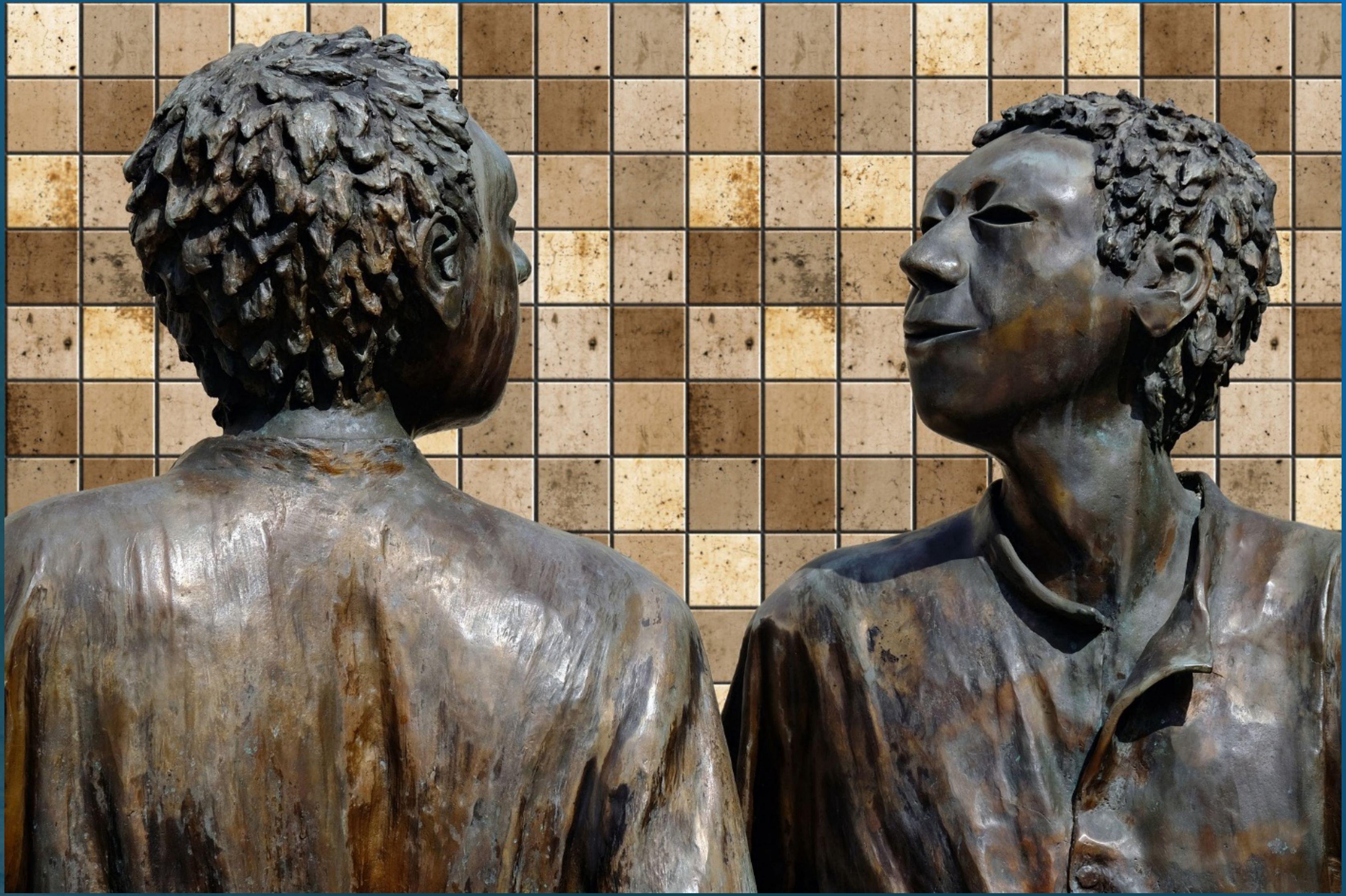

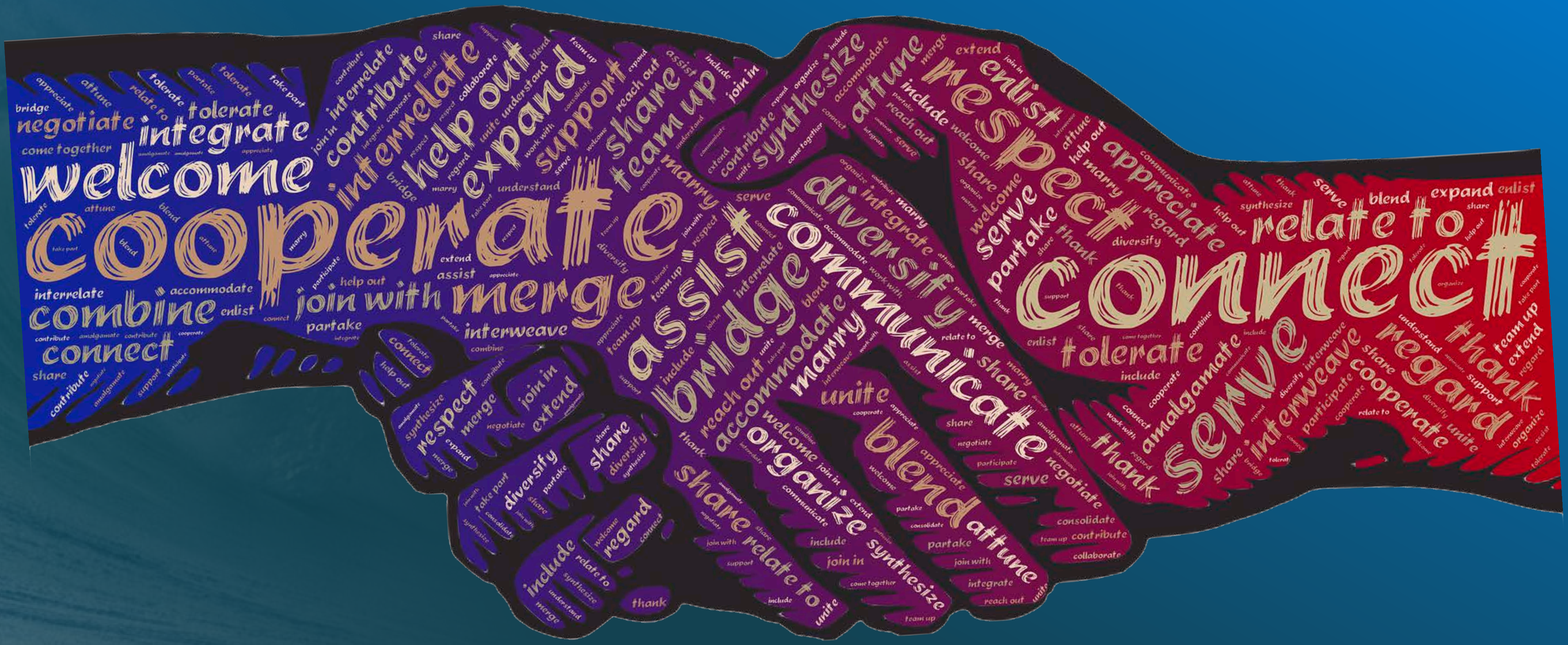
cooperate
welcome
negotiate
integrate
tolerate
combine
connect
merge
join with
interweave
assist
bridge
communicate
diversify
respect
connect
relate to
serve
tolerate
regard
thank
blend
organize
accommodate
expand
help out
support
share
team up
synthesize
attune
appreciate
unite
amalgamate

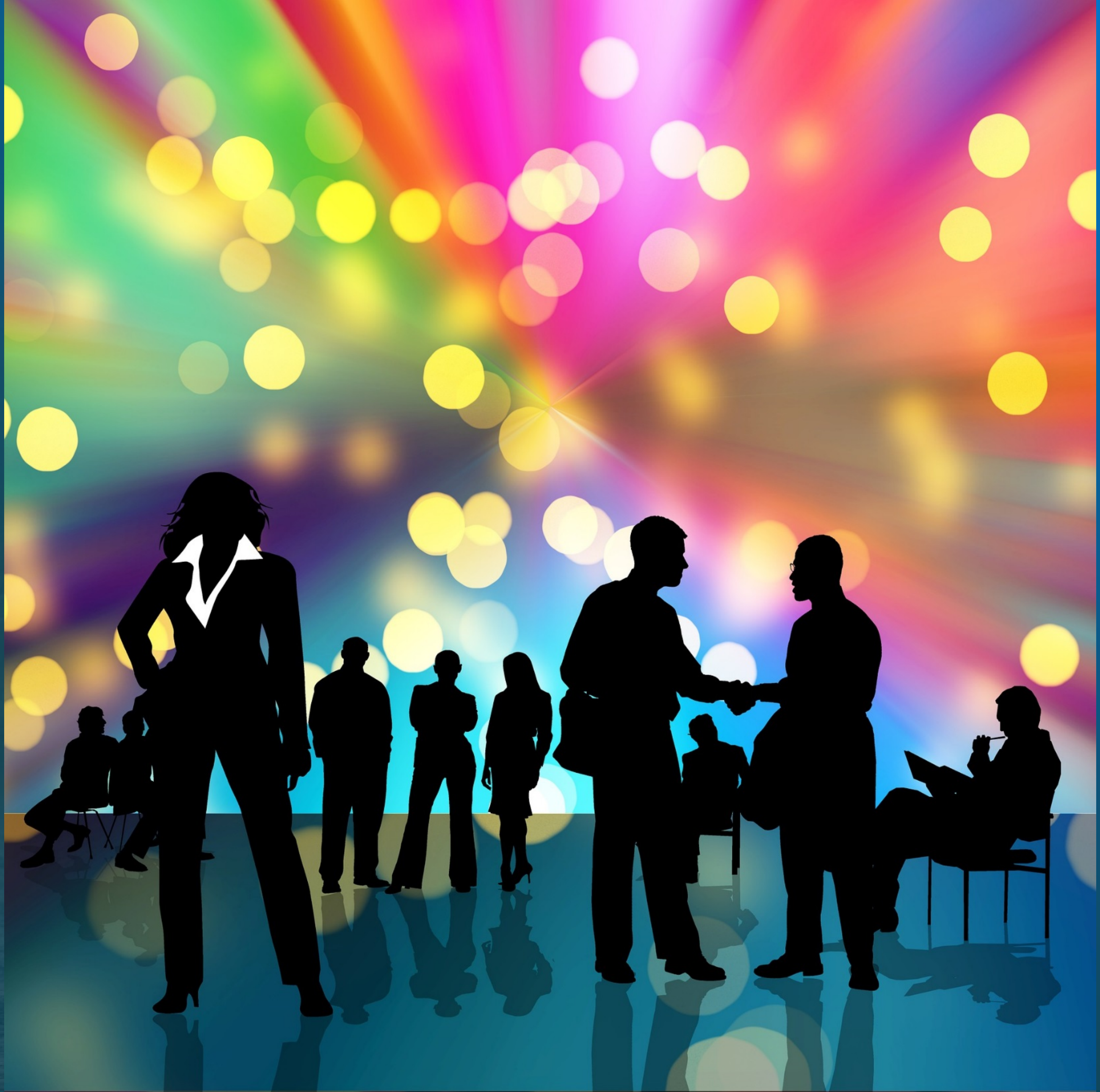

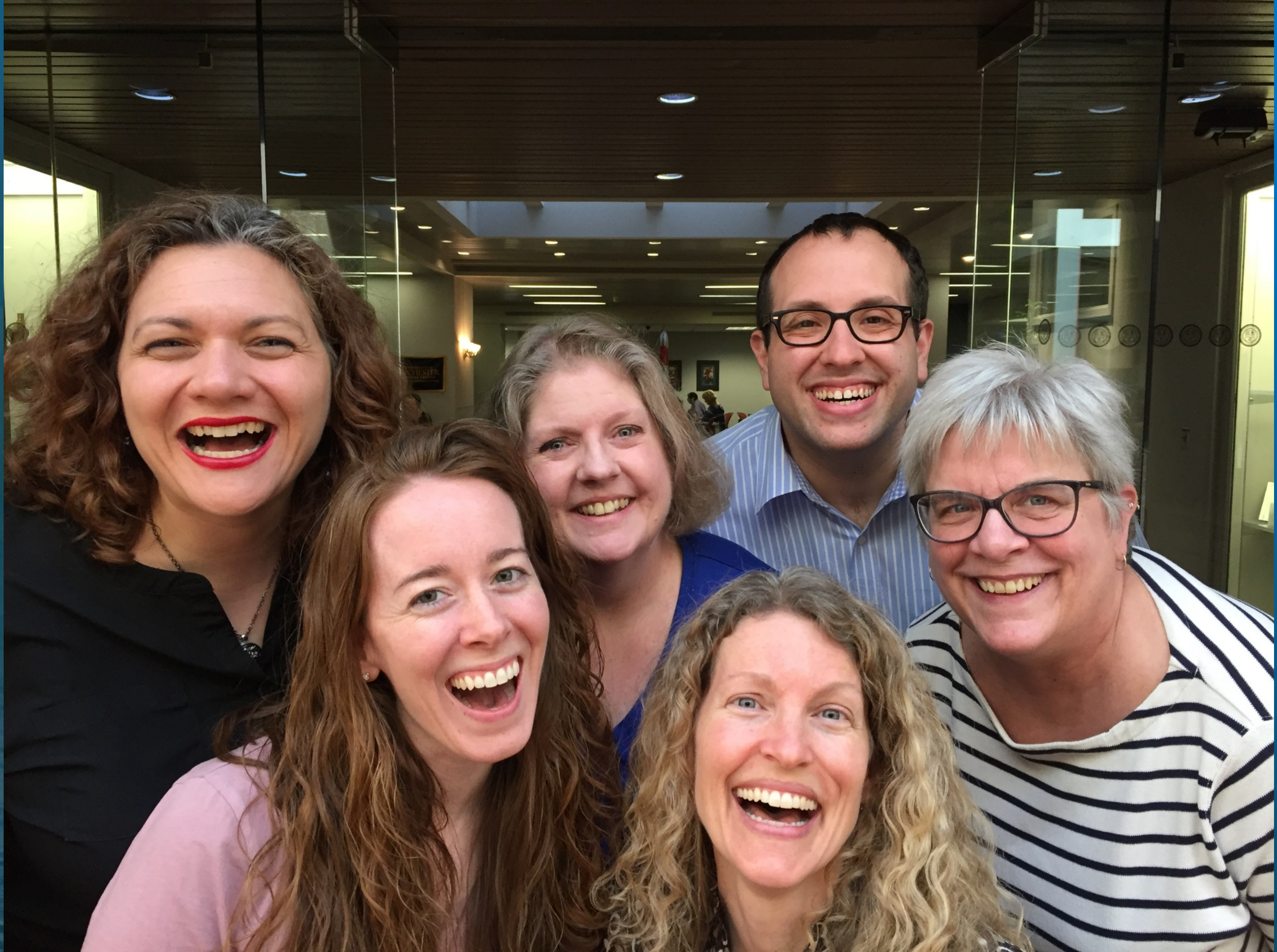

You can't always be right, but you can always be kind.

-- Robert J. Joynt, MD, PhD

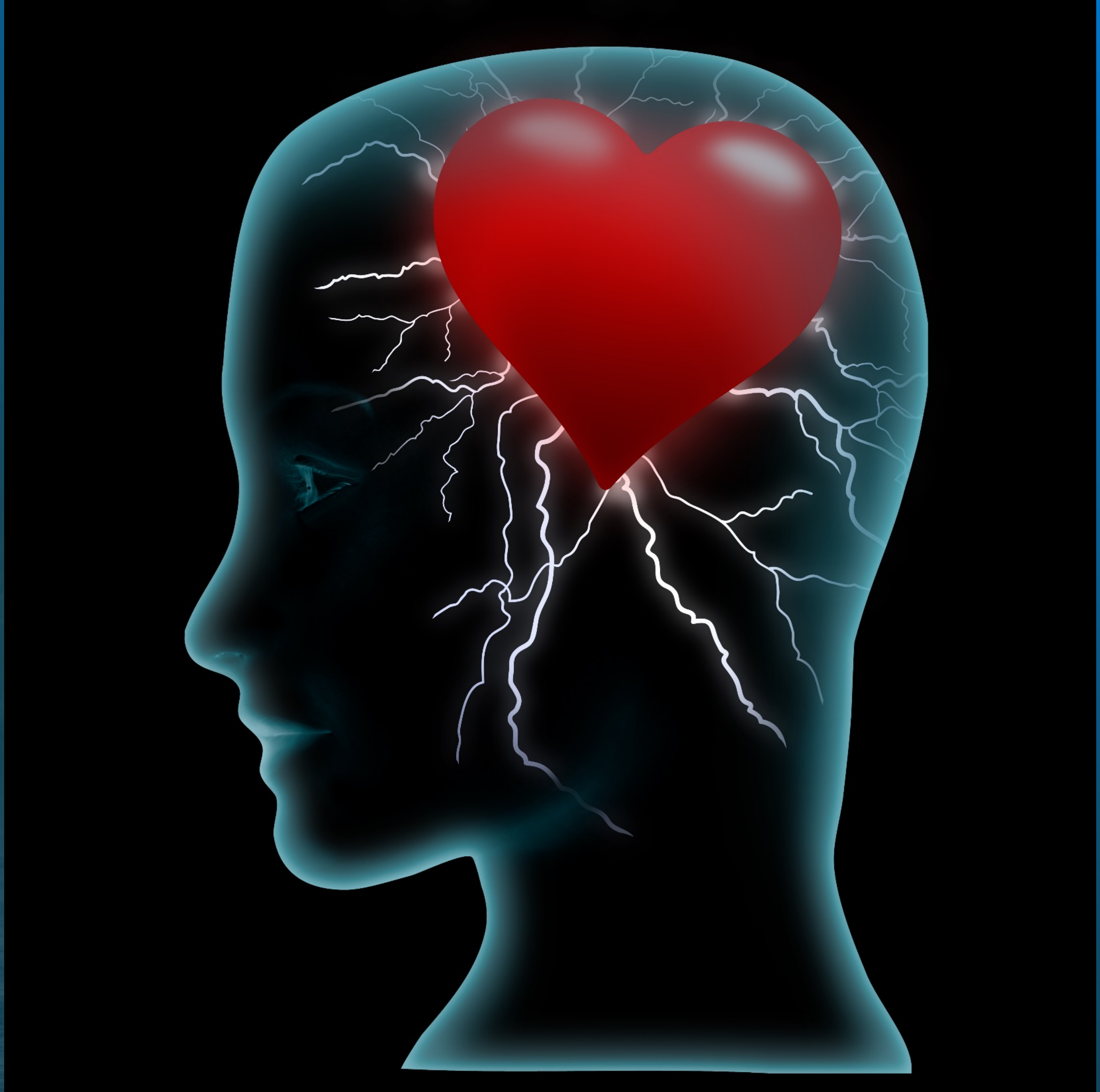

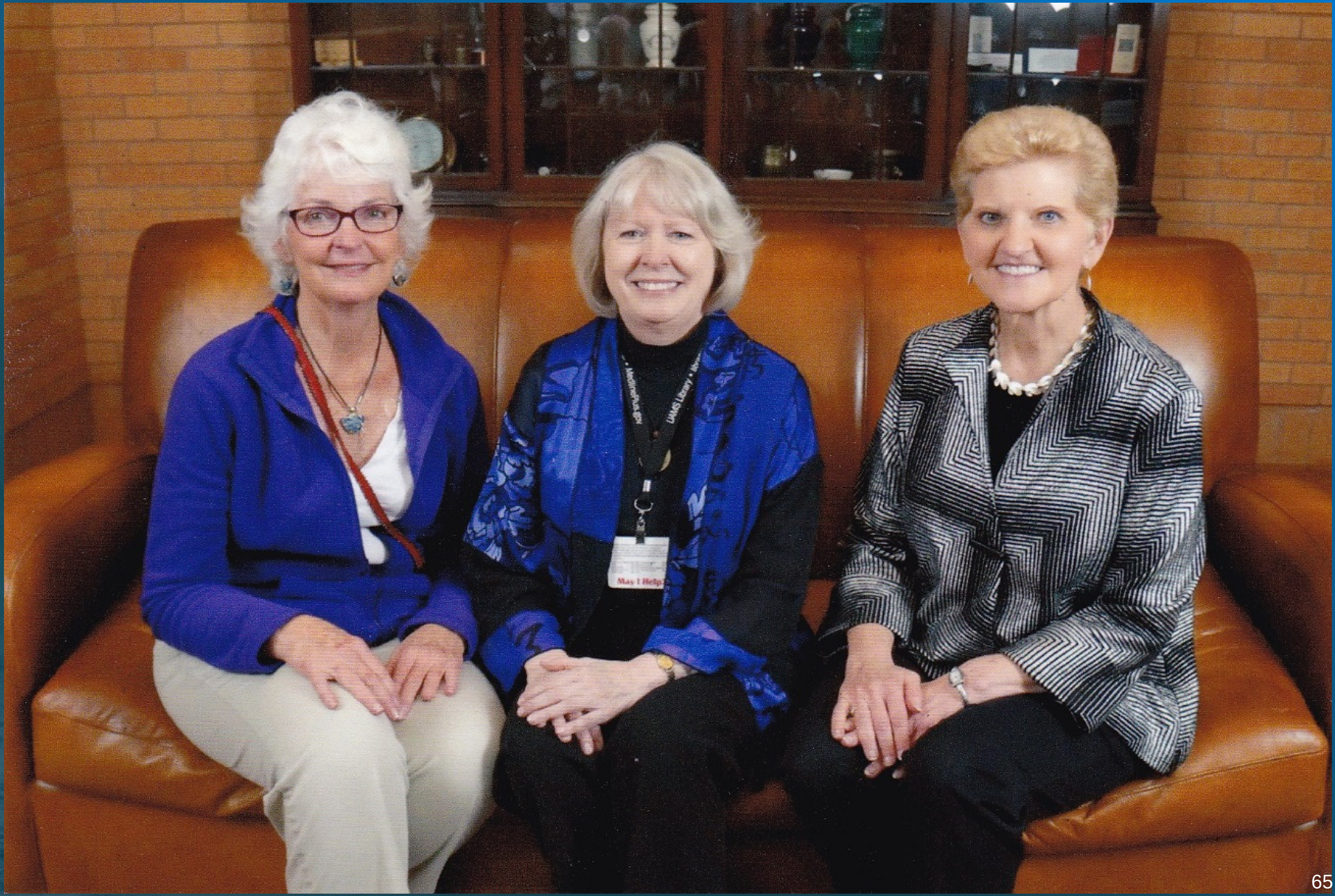
May I Help

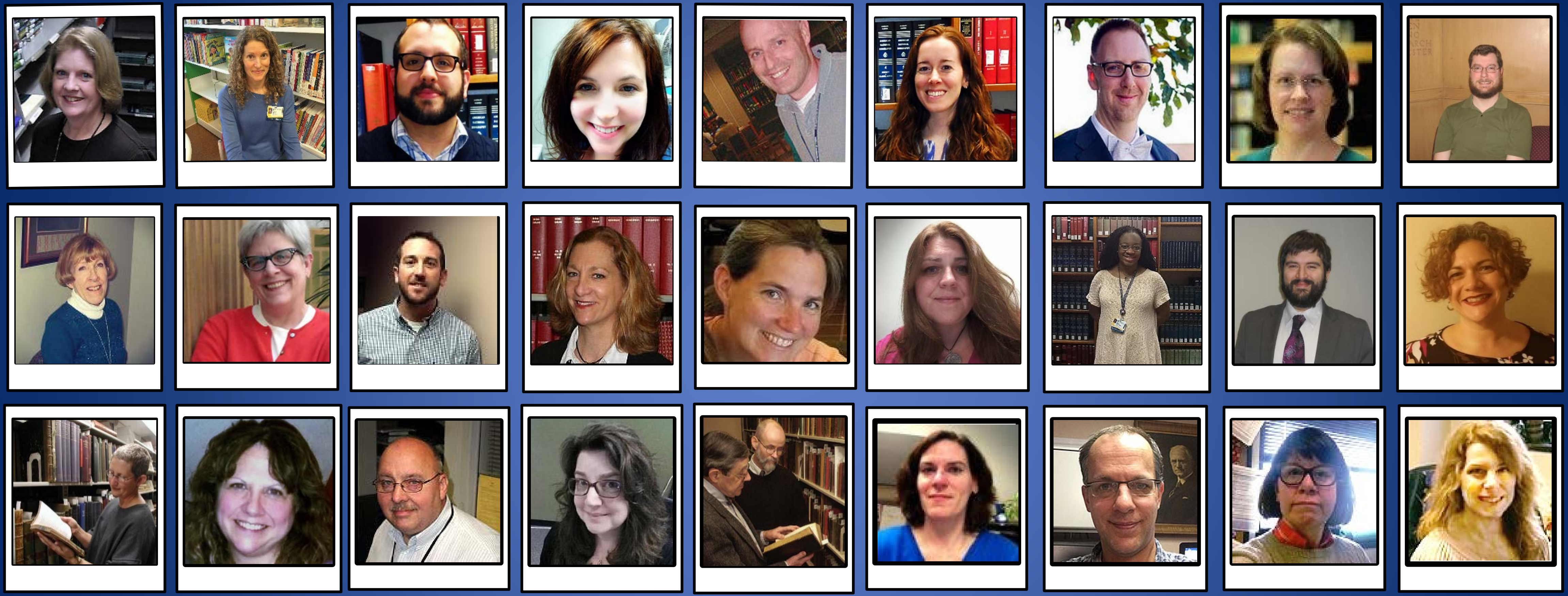

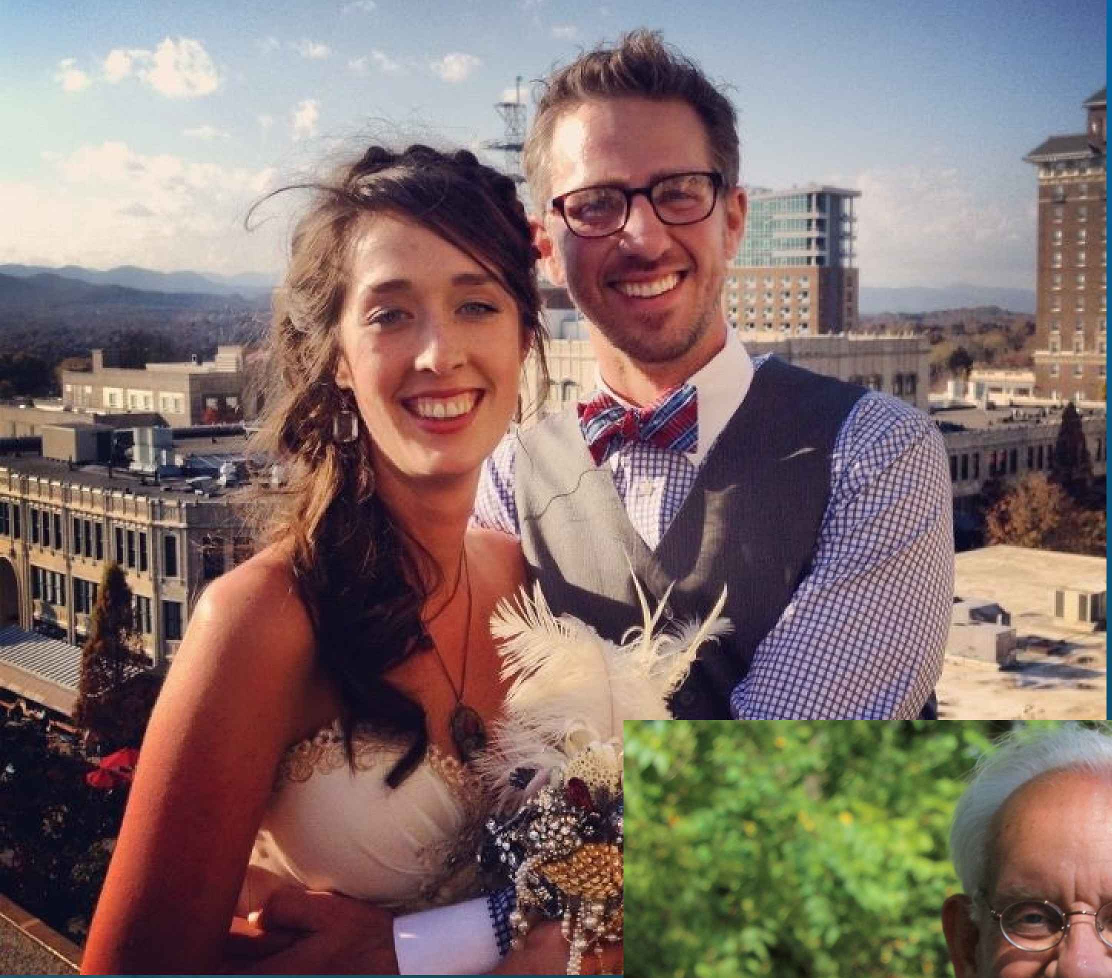

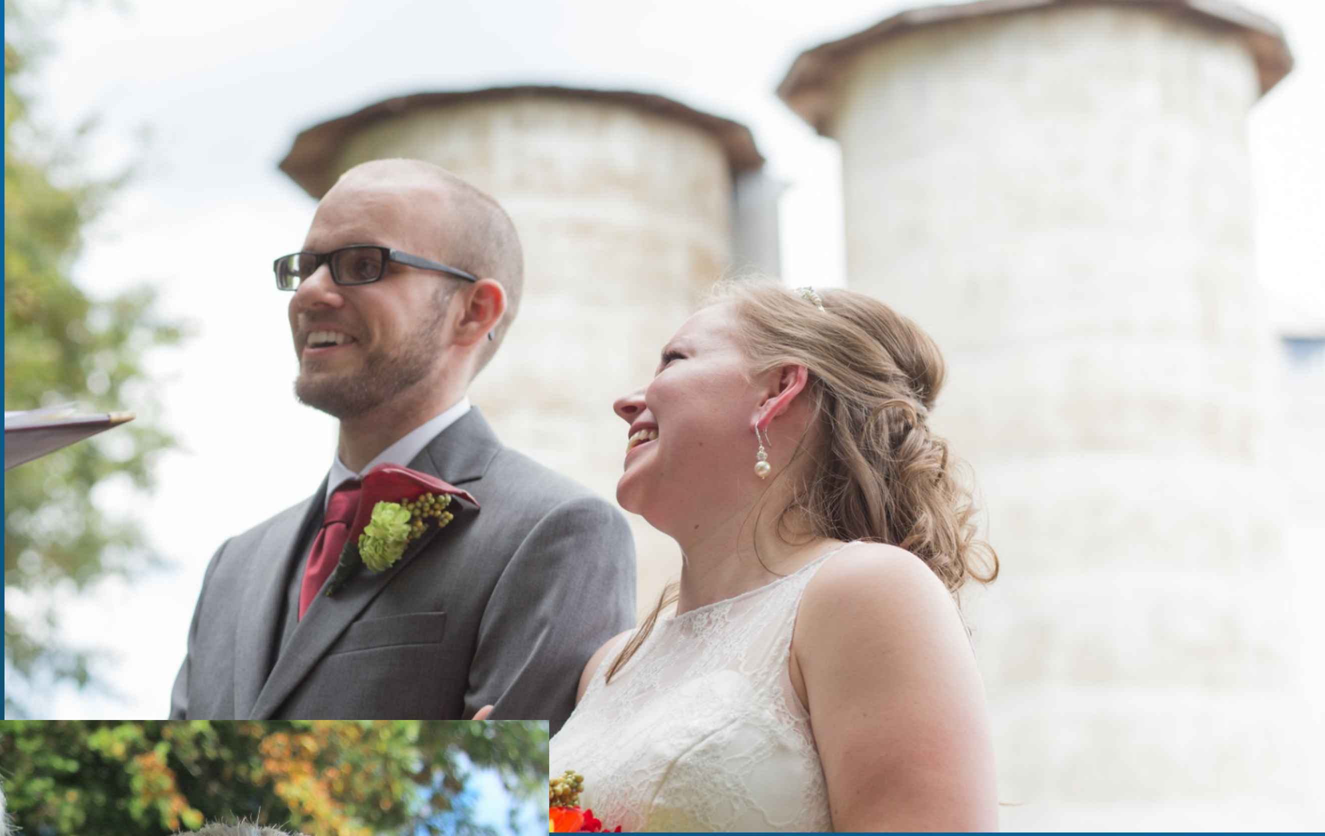

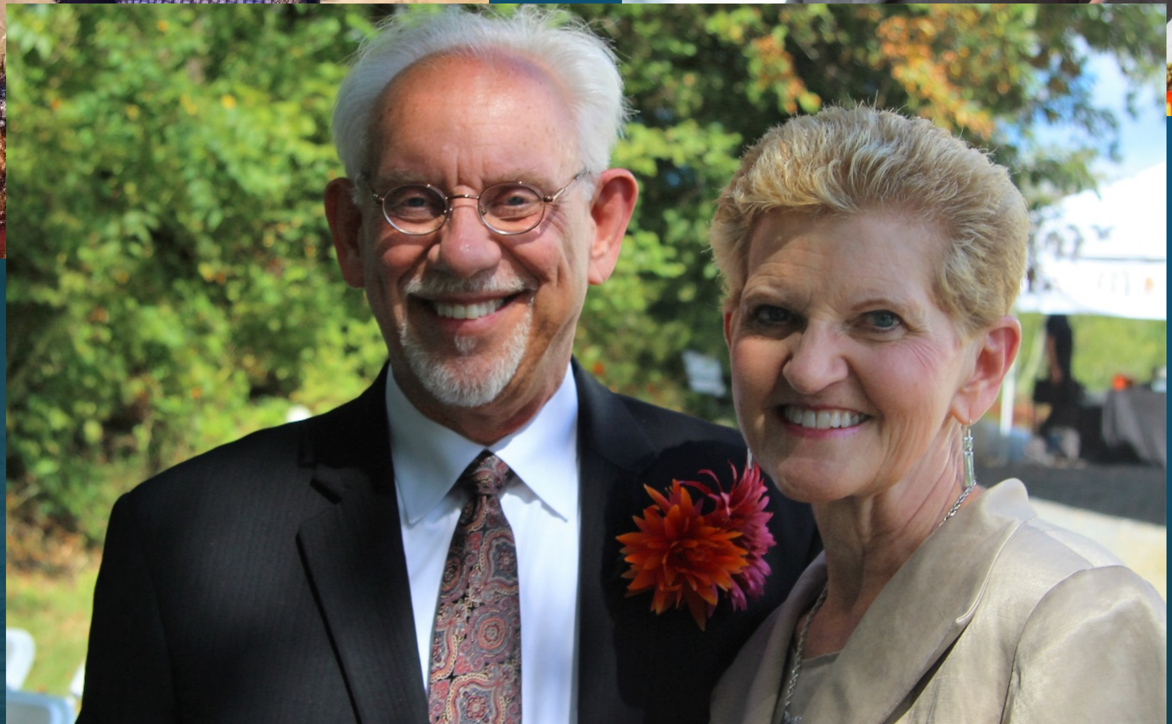

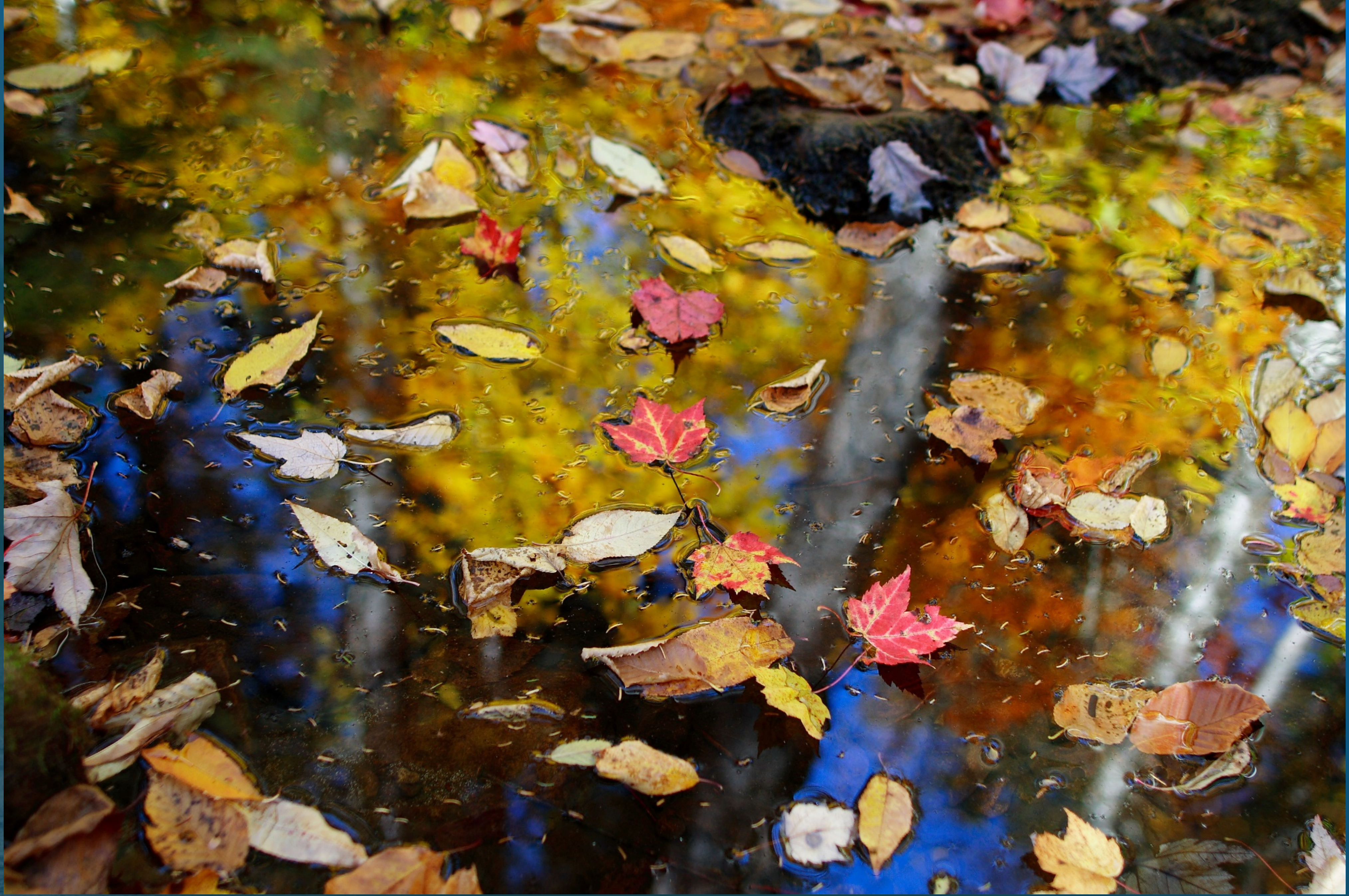

http://www.ForestWander.com
