# Supplementary material for: Looking inside ourselves: a culture of kindness
Source: J Med Libr Assoc. 2018 Jul 1;106(3):283. doi: 10.5195/jmla.2018.478 (PMC6013138; doi:10.5195/jmla.2018.478)
Supplement: Appendix B [file jmla-106-283-s002.pdf]

JMLA Journal of the Medical Library Association

SC

Supplemental content to
J Med Libr Assoc. Jul;106(3):dx.doi.org/10.5195/jmla.2018.478
www.jmla.mlanet.org
© Sollenberger 2018

Looking inside ourselves: a culture of kindness

Julia F. Sollenberger, AHIP, FMLA

APPENDIX B

Transcript of the 2017 Janet Doe Lecture

SLIDE 1: TITLE

Good morning, everyone! And thank you, M.J., for your very kind words. M.J. has been so supportive of me during this past year. She knows what an honor and what a challenge it is to be chosen to present this Janet Doe Lecture. In typical M.J. style, she helped me find the humor (she's the one with the humor) in the process by presenting me last year with this sweet little "Monkey on my back."

SLIDE 2: MONKEY

Now, this little monkey has been peering over my monitor all year long. Oh, I called her Janet, by the way. Instead of having Janet be there and thinking of her as a worry (although it was), I tried to turn her into a positive. So, as I said, she's been with me on that monitor all year. It helps to have a constant companion when you're working on a really tough task! Thank you, M.J., for Janet and for your humor, your kindness, and support.

Practically every Janet Doe lecturer has shared his or her thoughts at having been bestowed this great honor. I was indeed honored; I was also terrified. This is what everybody tells you, and it's true.

SLIDE 3: LUCRETIA McCLURE, AHIP, FMLA

So what did I do to get started? I went to my greatest champion, my friend and colleague, the phenomenal lady so many of us know and love and respect: Lucretia McClure, AHIP, FMLA (who could not be here today, unfortunately, but she told me she'd be thinking of me). I asked her if she had any advice for me as I began to choose my topic, to consider what I could possibly say that would be worth listening to. She said, "Honey, you have to talk about a topic you're passionate about. If you do that, the rest will take care of itself." Well, it did not necessarily take care of itself, (no one handed me that finished script), but it did make the research and the writing more exciting and rewarding.

SLIDE 4: LOOK BACK

So the Janet Doe Lecture is supposed to explore the history or philosophy of our profession. Many people look back, at the history of MLA or of librarianship. How did we get where we are? What are the lessons learned, and how do we apply them to our current situation?

SLIDE 5: LOOK AHEAD

Some people look ahead. They make predictions about the future, they paint a picture of where the profession is headed. Neither one of those is a strength of mine; I am, I'm sorry to say, not a history buff, and I sometimes struggle when I'm asked to be visionary.

SLIDE 6: RALPH WALDO EMERSON QUOTE

So I decided not to look back or to look ahead, but rather to look INSIDE. That's easier for me.

Ralph Waldo Emerson is often credited as saying, "What lies behind us and what lies ahead of us are tiny matters compared to what lies within us" [1].

SLIDE 7: HEART BRAIN CONNECTION

So we're going to take a little journey, looking inside ourselves and considering how the heart and the mind and the brain are connected.

Supplemental content to
J Med Libr Assoc. Jul;106(3):dx.doi.org/10.5195/jmla.2018.478
www.jmla.mlanet.org
© Sollenberger 2018

SLIDE 8: PENNSYLVANIA DUTCH FOOD

Well, and maybe the stomach and taste buds are connected, too! I grew up in New Holland, Lancaster County, Pennsylvania—the heart of Pennsylvania Dutch country—and there were two things that stood out about my childhood. Guess what the first one was? Food—gooey shoo fly pies; sticky sticky buns; buttery apple raisin donuts; crispy crullers covered with King Syrup; chocolaty whoopee pies; soft and creamy vanilla long johns; sweet and tart chow-chow; my Aunt Martha's double-coated fried chicken; and, best of all (and my kids will agree with this), Aunt Stella's melt-in-your-mouth buttermilk-iced cookies. I could go on listing more mouth-watering delicacies from my childhood for the entire hour! But I think you'd only want me to do that if I also handed out a few of them. Sorry. But, as I think back, truly, some of the most vivid memories have to do with food.

SLIDE 9: PENNSYLVANIA DUTCH PEOPLE

The other thing that stood out was the kindness of those around me. The Pennsylvania Germans have inhabited Lancaster County for a couple hundred years and still do today: the Amish, Mennonites, the Church of the Brethren (that is the denomination I grew up in, which is related to Mennonites and Quakers in its basic beliefs about peace and a commitment to service). As I grew up in this fertile farming country, I was surrounded by kind and loving people, people who demonstrated honesty and integrity, kindness and compassion, humility and authenticity. My parents, my siblings, my extended family, my neighbors, and my community made it clear that a life worth living was a life that cared about and served others. I'm sure that contributed to my chosen professions, first in education as a high school math teacher and then as a medical librarian.

SLIDE 10: WHY?

I'd like to take just one minute, and I want to ask you to turn to the person next to you and talk about why *you* are a medical librarian. What is it about being in the health care environment that might make you to go to work every day? For those of you who are NOT medical librarians (like my husband and sons and their wives), just talk about why you love your job (if you do). I'll bring you back in one minute.

[CHIME SOUND]

I would guess that many of you said that you are a medical librarian because you like being part of a system that ultimately helps people, helps patients, improve their health. You like making a difference in the lives of those patients, either directly or indirectly. How many said something along those lines? There a lot of hands up there. Oh, good. I agree. Even in research, it's really the patient-centeredness that is the ultimate goal.

Now, all right, although I said I wasn't going to look back, no Janet Doe lecturer can say she has done her job if there isn't just a bit of history intertwined. So, we will look back for just a bit.

SLIDE 11: ALAN REES

The Janet Doe Lecture that Alan Rees presented twenty-five years ago explored "Communication in the Physician-Patient Relationship" [2]. Rees, the author of seven editions of the *Consumer Health Information Source Book,* reviewed the changing nature of the interactions between physicians and patients. He suggested that health care in 1992, with all its tests and technology (technology at that point), was far removed from the care that patients experienced at the turn of the twentieth century when the physician's role consisted mostly of comfort and healing. At about that time, Oliver Wendell Holmes, in fact, advised people to choose a physician as they might choose a friend [3].

SLIDE 12: HAROLD ANDERSON ILLUSTRATION

Because there was so little known about how to actually cure an illness, family physicians were instead caring for the patient, the person. A distinguished professor at Harvard Medical School, F.W. Peabody, said in 1930 in his book *Doctor and Patient*, "What is spoken of as a clinical picture is not just the

Supplemental content to
J Med Libr Assoc. Jul;106(3):dx.doi.org/10.5195/jmla.2018.478
www.jmla.mlanet.org
© Sollenberger 2018

photograph of a man sick in bed; it is an impressionistic painting of the patient surrounded by his home, his work, his relatives, his friends, his joys, sorrows, hopes and fears" [4]. Every visit was "an occasion for warm conversation in addition to medical treatments." Now, in my opinion, of course, there were the great negatives of paternalism and dependency, but the physician did generally act as friend and showed compassion and empathy.

SLIDE 13: PATIENT IN BED SURROUNDED BY DOCTORS

By the mid-twentieth century, that relationship was practically severed, in the gradual transition from home visits by family doctors to care by specialists in the hospital. Medical institutions were preoccupied with tests and technology (again, not very advanced at that point, but still technology), and they pushed to the edges the human dimensions of medicine. It was an overly complex system; patients began to feel isolated and considered "just a number."

SLIDE 14: BIOPSYCHOSOCIAL MODEL OF HEALTH AND DISEASE

In the late 1970s, a prominent internist and psychiatrist who practiced and taught at the University of Rochester wrote a groundbreaking article in *Science* called "The Need for a New Medical Model: A Challenge for Biomedicine" [5]. George Engel wanted to help the profession see the inadequacy of providing care based solely on the biology and chemistry of disease. He introduced the "Biopsychosocial Model," and he suggested that patients' psychological makeup and social relationships were as important to illness and health as the biological, genetic, and molecular aspects of disease.

SLIDE 15: BIOPSYCHOSOCIAL APPROACH

The patient was at the center of a continuum, with the molecular, cellular, and organ systems on one end (there at the bottom) and the family, community, and society on the other [6]. Engel believed in a humanistic approach, suggesting that what patients reported about their illnesses, in their own words, was as important as any lab test or X ray. "Engel liked to say that what patients want most is to know and understand what is happening to them, but also to be known and understood" [7].

SLIDE 16: GEORGE ENGEL, MD, AND JOHN ROMANO, MD

This biopsychosocial model of clinical practice was translated into the education of medical students and residents. Well before his 1977 paper, Engel, along with John Romano, a well-known psychiatrist and chair of the Department of Psychiatry at Rochester, were at work in Rochester demonstrating that a well-conducted medical interview was the single most important thing that a physician could learn [8].

SLIDE 17: GEORGE ENGEL, MD

I will tell you what one student and observer of George Engel said about his bedside manner. (That's George right there.) Ron Epstein is a professor at the University of Rochester and a practicing family physician. He was a resident and a fellow under Engel, and he observed:

> Engel's extraordinary capacity to connect with and know other human beings. Patients would meet him for the first time and reveal themselves in ways that provided clues to their medical diagnoses while also creating a human bond. Engel would *look* curious, with a quizzical gaze. He'd ask, "What happened next?" and "What were you thinking when you did that?" He'd keep asking until he got a full visual picture of the patient's home, and family, and habits, and ideas about what was going on. He'd say, "I wonder…" and you'd *feel* a sense of wonder. Patients felt understood. [7]

SLIDE 18: PATIENT AND PHYSICIAN

Remember that visual picture that the family practitioner was able to get by visiting the patient's home? Well, Engel did a similar thing, using words to visit the patient's thoughts and feelings. As a result, Rochester continues to be known for its unusually patient-focused and family-oriented care; the medical school teaches and honors the tradition of caring for the whole patient.

Supplemental content to
J Med Libr Assoc. Jul;106(3):dx.doi.org/10.5195/jmla.2018.478
www.jmla.mlanet.org
© Sollenberger 2018

SLIDE 19: DOUBLE-HELIX CURRICULUM

In fact, when the medical school curriculum was revamped in 1999, when something called the "Double Helix Curriculum" was created, the goal was to integrate the biological, psychological, and social foundations of medical practice across the four years of medical education, at the same time building on evidence-based and scientific aspects of medicine. There are two strands of the Double Helix Curriculum: clinical (patient-centered) and biomedical (science-based); and those are interwoven across the entire medical school experience [9].

So in Rochester, and really in all of our health care organizations these days, the caregiver-patient-family relationships are really considered paramount in providing exceptional care. Patient- and family-centered-care is the catchphrase, and it provides direction for many initiatives in our health care environments.

SLIDE 20: RONALD EPSTEIN, MD

Let's go back to that student of George Engel, Dr. Ron Epstein. He is the author of a landmark article, published in *JAMA* in 1999, an article that helped the medical profession see a clear link between evidence-based medicine and relationship-centered care [10]. That link was "Mindful Practice," the title of the article. Epstein introduced a revolutionary concept: that by looking inward, health care practitioners could grow their capacity to provide high-quality care.

SLIDE 21: *ATTENDING*, BY RONALD EPSTEIN, MD

In his book *Attending*, just published in January of this year, Epstein says that by learning to be mindful—attentive, present, listening, focused—health practitioners can better show the compassion, kindness, and humanity that patients need and deserve. They can have more meaningful relationships with their patients and simply be better clinicians! Epstein and many other mindful practitioners at Rochester and across the globe have been successful at training practitioners to become more mindful. They have measured the difference it can make in being more attentive, more empathetic, more emotionally stable, and they have found that it really works—in helping caregivers provide a higher quality of care [7].

So I'd like to return to a *personal* story. This time it's not about food, but about my experience with mindfulness.

SLIDE 22: MINDFUL PRACTICE WORKSHOP

I was still in the early stages of thinking about my Janet Doe lecture topic when I saw this email invitation in my inbox: "Mindful Practice Workshop" [11]. There were words and phrases like "quality of caring," "compassion," and "self-care." I knew Ron Epstein, one of the codirectors. I was somewhat familiar with his work on mindful practice and doctor-patient communication, and thought this might be really fascinating, and related to my potential topic. My boss agreed to pay for it (we didn't have enough money in our library budget), and I registered. I had taken my first step.

I was really pretty apprehensive. Would I be able to be a contributing member of this group if I wasn't a clinician? The flyer did say, "designed for medical practitioners and others involved in medical practice *and education*. No prior experience is required." OK, I was involved in medical education; I had had no prior experience. It would be OK.

I can be pretty quiet and reserved when I'm around people I don't know. I was in some ways really dreading the retreat, but obviously also intrigued and looking forward to it.

SLIDE 23: ZEN CENTER

The three-day retreat was held in a Zen Center in a remote location, in the woods, about forty miles west of Rochester. I was fortunate to have a single room. It was pretty sparse: a bed with a bedside stand, a chair, and a lamp, and bathrooms that were down the hall. I was thankful that I was in a single room and I wouldn't be forced to engage in mindless (or was that mindful?) chit-chat with a roommate.

Supplemental content to
J Med Libr Assoc. Jul;106(3):dx.doi.org/10.5195/jmla.2018.478
www.jmla.mlanet.org
© Sollenberger 2018

SLIDE 24: DINNER AT MINDFUL PRACTICE WORKSHOP

We started with dinner that first night. It went OK. I sat with a couple of nurses from Detroit who worked in a transplant unit. They were nice enough, and we were able to keep the conversation going through the meal. At the first session, I found out that there were maybe forty-five people there from all across the United States and the world—the Netherlands, Chile, Australia, Germany! Who knew this was going to be such a big deal? I really didn't know that these workshops and their instructors were so well known.

SLIDE 25: MEDITATION ROOM

We were told to show up the next morning at 6:30 a.m. for *an hour* of meditation before breakfast. Oh my god! I had never done this before, and I was going to meditate for an hour, in the Zen meditation room?! The next morning, quaking in my slippers (we wore socks or slippers and not street shoes during the entire retreat), I joined the others in the stillness and low light of dawn, sitting cross-legged on a yoga cushion. Everything was quiet, save the chime that got us started.

[CHIME SOUND]

There was some guided meditation, helping us to focus on our breath, and I felt like a fish out of water. After about twenty minutes of silence, we did a walking meditation, following each other quietly around the perimeter of the room, which was walled off the main part of the room by partitions. We settled back on our cushions and were led in more guided meditation, with a poem that our meditation leader had just written for that morning meditation (as it referred to some things that had been said the night before by some of the participants). Wow! By this time I was squirming; my back was hurting; and I was trying to figure out how I'd survive the final twenty minutes. But unexpectedly at that point, I suddenly felt like I was becoming acclimated. I felt focused and calm and thought that maybe I could indeed survive the next three days! And I did!

I learned a lot during that retreat. I learned that these physicians, nurses, and other caregivers felt deeply about their patients. I learned that they worry that they'll make mistakes. I learned that there are guided meditation apps that might help me keep doing this meditation thing, if I wanted to. I learned that everyone who is a beginner in meditation feels like they don't know what they're doing; it takes time and practice. I learned during a dinner conversation that some of the physicians from Rochester didn't know that the library was available to help patients find information; now a few more did. I learned that I welcomed the quiet that the one longer silent meditation period afforded (at one point, we were silent from the beginning of an evening session until the following morning after breakfast).

SLIDE 26: AUTUMN DAY

I learned that a beautiful, blue-skied autumn day in the woods provided just the right setting for experiencing *outside* the calm and quiet that we had experienced *inside*.

I *thought* I learned that a guy with the last name of Zen had started all this mindfulness meditation stuff in the health care environment. How could that be?!

SLIDE 27: JON KABAT-ZINN

Turns out his name is Jon Kabat-Zinn. I had only heard the retreat instructors talk about him, but I had never seen it in print. I felt a little unaware. I'm sure many of you know about Jon Kabat-Zinn. He started the Mindfulness Based Stress Reduction (MBSR) program at the University of Massachusetts, but I hadn't heard of him prior to this experience.

Jon Kabat-Zinn is the "father" of the use of mindfulness in Western medicine who first developed the MBSR program, used today in medical centers, hospitals, and clinics around the world. He is the founder of the Stress Reduction Clinic at the University of Massachusetts and its Center for Mindfulness in Medicine, Health Care and Society. Kabat-Zinn's definition of mindfulness is the one used by many

Supplemental content to
J Med Libr Assoc. Jul;106(3):dx.doi.org/10.5195/jmla.2018.478
www.jmla.mlanet.org
© Sollenberger 2018

others in this field: "Mindfulness is awareness, cultivated by paying attention in a sustained and particular way: on purpose, in the present moment, and non-judgmentally" [12].

SLIDE 28: RON EPSTEIN, MD, AT MINDFULNESS RETREAT

Ron Epstein's definition of *mindful practice,* applying mindfulness to the practice of medicine, is "moment-to-moment purposeful attentiveness to one's own mental processes during every day work with the goal of practicing with clarity and compassion" [10].

Here's another story about my mindfulness retreat. Our first real "exercise" was one called "Meaningful Experiences." Here are the instructions that we received: "Focus on a time during your work as a clinician or educator that was particularly meaningful to you. Perhaps it was a time when you were moved in some way, or may have been a time associated with great joy or great sorrow. Take a few minutes to write a brief narrative about the experience, and then you're going to share it in pairs." The listener was to spend most of the time listening and asking questions aimed only to deepen understanding. We were not to interrupt, make interpretations, give advice, or talk about ourselves.

It was just my luck to be paired up with another of the codirectors of the retreat. I was nervous about this because I, of course, didn't have a patient experience to relate (although no one had said it had to be a patient), but I felt like my story would somehow be out of place. I forged on, of course, and put together a story about my experience laying off an employee of more than thirty-five years because of budget reductions. She was a person I had known for all that time and who had had a pretty hard life. But she was doing a job we didn't need to have done anymore, and she wasn't successful at learning the new technology we needed her to learn.

SLIDE 29: TEARS

So when I told my story, I had tears come to my eyes. My children will tell you—I'm a crier. (Apparently, this is a trait I share with our MLA President Teresa L. Knott, AHIP!) When this happened I thought, "Oh no, Julie. You are such an emotional wreck; you are not at all being professional right now." My partner, I'll call him Peter, was very understanding; he asked some good questions, but I still felt like I had botched it. Then it was his turn. I cannot tell you his story. Peter is a neurologist and geriatrician who cares for Alzheimer's patients and their families. But when he told his story, he had tears come to his eyes, too! I was moved, as I am now. He clearly felt very deeply about his patients. I really didn't know, before that storytelling experience, the emotional intensity that caregivers (at least mindful caregivers) can experience, related to the patients they care for.

SLIDE 30: ORGANIZATIONS

In the health care world, there are many organizations that have been created over the past twenty to thirty years that focus on aspects of mindfulness. In addition to the Center at UMass created by Kabat-Zinn, there's the Center for Compassionate Care and Altruism Research and Education at Stanford. It was founded by a neurosurgeon and regular meditation practitioner, Dr. James Doty [13]. In New Zealand, there is an organization called "Hearts in Healthcare," founded by Dr. Robin Youngson, an engineer turned doctor who has been an advisor to the New Zealand government and to the World Health Organization. Youngson is author of the book *Time to Care: How to Love Your Patients and Your Job* [14]. And then there's the Schwartz Center for Compassionate Healthcare. This nonprofit was founded with funds provided by Kenneth Schwartz, a cancer patient who wanted to encourage the sorts of caregiver-patient relationships that had made all the difference to him during his illness in the months and days leading up to his death [15].

SLIDE 31: SCHWARTZ ROUNDS

The Schwartz Center is a particularly interesting, and effective, organization that is leading the movement to bring compassion to every interaction between patient and caregiver. The Schwartz Rounds program, occurring in more than 425 health care institutions throughout the United States and Canada

JMLA Journal of the Medical Library Association

SC

Supplemental content to
J Med Libr Assoc. Jul;106(3):dx.doi.org/10.5195/jmla.2018.478
www.jmla.mlanet.org
© Sollenberger 2018

and more than 120 throughout the United Kingdom, offers health care providers a regularly scheduled time to openly and honestly discuss the social and emotional issues that they face in caring for patients and families [16]. As opposed to traditional medical rounds, the focus is on the human dimension of medicine. Caregivers have an opportunity to share their experiences, thoughts, and feelings on topics that are derived from actual patient cases. The premise is that caregivers are better able to make personal connections with patients and colleagues when they have greater insight into their own feelings and emotional responses. Turns out we have Schwartz Rounds at Rochester that I was never even aware of; I found out about that by reading about them in the book by Ron Epstein that I mentioned earlier.

SLIDE 32: RESEARCH ORGANIZATIONS

There are some organizations that focus on research about mindfulness, either behavioral research, or research that studies the neuroscience of the brain as it relates to meditation, mindfulness, compassion, and kindness. The University of California–Los Angeles (UCLA) Mindful Awareness Research Center; the Center for Investigating Healthy Minds at the University of Wisconsin; and the Greater Good Science Center at Berkeley.

SLIDE 33: SLIDE WITH MAGAZINE COVERS

There are so many of these entities that have cropped up that it really does sometimes feel like a fad of the month, or the year, or the decade [17]. But I suggest that these groups are doing the good work of helping health care organizations realize that compassion and kindness are foundational characteristics of good medical care, that the patient's and the caregiver's innate need for human connections requires listening, respect, empathy, and strong communication and interpersonal skills.

SLIDE 34: NEUROSCIENCE OF THE MINDFUL BRAIN

Let's return to these research groups around the country and the world that are using sophisticated brain imaging techniques to study the neuroscience of the mindful brain. Some call this contemplative neuroscience; others affective neuroscience; still others, interpersonal neurobiology. The following three books helped me get a glimpse into why kindness and compassion are good for you, and also why they can be learned.

SLIDE 35: DAVIDSON BOOK

1. *The Emotional Life of Your Brain*, by Richard J. Davidson, PhD [18]. He is professor and director of the Waisman Laboratory for Brain Imaging and Behavior, and the Laboratory for Affective Neuroscience; he is also founder and chair of the Center for Investigating Healthy Minds, all at the University of Wisconsin–Madison.

SLIDE 36: SIEGEL BOOK AND HAMILTON BOOK

2. *The Mindful Brain*, by Daniel J. Siegel, MD [19]. Siegel is a graduate of the Harvard Medical School and codirector of the UCLA Mindful Awareness Research Center.
3. *Why Kindness Is Good for You,* by the Scottish scientist David R. Hamilton, PhD [20]

SLIDE 37: DOPAMINE

On a biochemical level, the good feeling that we get when we experience a kindness (either by being the receiver or the giver) is due to the elevated levels of the brain's endogenous opioids. They cause elevated levels of dopamine in the brain, helping us lift our moods and making us feel more positive.

SLIDE 38: CORTISOL

At the same time, the stress hormone cortisol is suppressed, and blood pressure is reduced.

SLIDE 39: OXYTOCIN

In addition, when kindness involves an interaction between two people, a hormone called oxytocin is produced, which helps to strengthen bonds with others by making us feel more connected.

SC
Supplemental content to
J Med Libr Assoc. Jul;106(3):dx.doi.org/10.5195/jmla.2018.478
www.jmla.mlanet.org
© Sollenberger 2018

SLIDE 40: NEURAL PATHWAYS

In the longer term, practicing kindness retrains neural pathways in the brain. Study after study has shown, through brain imaging, that the adult brain can be transformed through experience [21].

SLIDE 41: OBOE

Let's take learning to play the oboe, for instance. And by the way, one of the first things I'm going to do after this Janet Doe Lecture, after I get home, is to learn to play the oboe. I start my lessons in June. Listen for the sound of an oboe a little later in the presentation; I will not be playing. The oboist's brain undergoes changes through a process called neuroplasticity. The brain regions that control the movement of the fingers and the position of the mouth and lips, as well as the control of the breath, become progressively larger with mastery of that instrument, or any instrument [18].

SLIDE 42: BRAIN IMAGING

A similar process appears to happen when we practice mindfulness. Neuroimaging has enabled scientists to observe activation of specific brain areas during meditation, mostly in the prefrontal cortex. This is the region of the brain that is important for many of the things that make us human, like judgment and decision-making, but also compassion, empathy, and kindness.

SLIDE 43: BRAIN CHANGES WITH MEDITATION

Brain imaging has helped scientists identify the neural networks that correspond to particular kinds of meditation. Meditation brings about changes in brain processes and in the volume of certain brain areas, possibly reflecting an increase in the number of connections among brain cells [18]. Other neuroscientists have worked to understand how emotions can affect the other body systems.

SLIDE 44: MINDFULNESS BRINGS IMMUNE SYSTEM CHANGES

Some have demonstrated that mindfulness training, while activating the prefrontal cortex, also boosts the participants' immune systems [22]. Other studies are examining whether or not mindfulness could help treat asthma. If patients could ward off stress reactions that can often bring on asthma attacks, perhaps they could have more control over their disease [18].

[CHIME SOUND]

SLIDE 45: LIBRARY WORKPLACES

I think it's time to pivot away from the world of neuroscience and health care and toward the world of our workplaces. For the most part, those of us here today work in medical libraries. We exist in the health care environment, but we have our own library organizations as well. As in all organizations, we try to get along with our colleagues, we work to bring together highly functioning teams, we strive to be leaders who deserve respect and loyalty. All of us move through our days, trying to demonstrate integrity, honesty, and kindness. There is that word, kindness.

SLIDE 46: "KIND" ON KEYBOARD

In the past decade, a great deal has been written about kindness and mindfulness in the workplace. Just since the year 2000, there have been almost 250 articles in the *Harvard Business Review (HBR)* and the *HBR Online* on mindfulness or meditation, kindness or compassion, or emotional intelligence.

SLIDE 47: LEADERSHIP ORGANIZATIONS EMBRACING MINDFULNESS

Untold numbers of leadership organizations and consulting and training companies have sprung up or have begun to provide education, coaching, and resources to help leaders and workers become more focused, more self-aware, and hopefully, more effective.

Here are some of the organizations that have embraced mindfulness in the workplace:

- The Center for Creative Leadership has done numerous whitepapers on "Empathy in the Workplace" [23] and "Mindfulness" [24].

Supplemental content to
J Med Libr Assoc. Jul;106(3):dx.doi.org/10.5195/jmla.2018.478
www.jmla.mlanet.org
© Sollenberger 2018

- The Mind and Life Institute is a nonprofit that sponsors international symposia and funds grants and scholarships to promote research in the field.
- The Institute for Mindful Leadership is a nonprofit started by a former vice president of General Mills and who is also a certified mindfulness trainer.

Yes, the corporate world has embraced mindfulness, and there is a growing use of mindfulness instruction in business schools. The Business and Management Schools at Stanford, NYU, Columbia, UC San Diego, Berkeley, Vanderbilt, Claremont, and London (many others) have incorporated courses in mindfulness in their master's of business administration (MBA) programs [25].

Nike, Facebook, General Mills, Aetna, Target, Intel, Green Mountain Coffee Roasters, Patagonia—these are companies that have all trained their employees in the practice of mindfulness [26].

SLIDE 48: GOOGLE

And then there's the most famous of all, Google. There's a mindfulness guru at Google, and his name is Chade-Meng Tan [27]. He calls himself Google's "Jolly Good Fellow, which Nobody Can Deny."

SLIDE 49: SEARCH INSIDE YOURSELF BOOK, AND DANIEL GOLEMAN

He is the cofounder and chairman of the Search Inside Yourself Leadership Institute, which provides experiential training that was first used at Google and is now offered across the globe [28]. His approach blends the concept of mindfulness with the contemporary field of emotional intelligence, the brainchild of Daniel Goleman. The forewords of Tan's book, called *Search Inside Yourself*, were written by Daniel Goleman and by Jon Kabat-Zinn [29].

Tan used this phrase "search inside yourself," because it was *Google*! The name first started out as a joke, but then it stuck. Because Tan is an engineer, a scientist, he says that everything he teaches is based in science—the neuroscience that we've already talked about, plus some social science and brain research that Goleman is involved in, at the Consortium for Research on Emotional Intelligence in Organizations, which is based at Rutgers.

Goleman and others have added another concept to their work on *emotional* intelligence [30], something called *social* intelligence [31]. He defines this as a "set of interpersonal competencies built on specific neural circuits (and related endocrine systems) that inspire others to be effective" [32].

SLIDE 50: MIRROR NEURONS

This is due to a class of brain cells called "mirror neurons" that, according to Goleman: "operate as neural Wi-Fi…When we consciously or unconsciously detect someone else's emotions through their actions, our mirror neurons reproduce those emotions." In organizations, "a leaders' emotions and actions prompt followers to mirror those feelings and deeds" [32].

So, we know that it's important to have the skills and knowledge to do our work. We need to be intellectually competent, just as the physicians we talked about earlier better be competent and knowledgeable, right? They're going to take care of us. That is indeed a requirement for a fully functioning health care organization or medical library. But if we don't also have these other pieces of emotional and social intelligence, then all the intellect in the world doesn't get us very far.

Emotions are central to building the culture of an organization. In a 2016 article in *Harvard Business Review,* "Manage Your Emotional Culture," two professors, (one at Wharton School of Business and the other at George Mason University) discuss the two kinds of culture at an organization [33].

SLIDE 51: COGNITIVE CULTURE

One is the cognitive culture: shared intellectual concepts, principles, and standards, like providing high-quality services or being innovative.

JMLA Journal of the Medical Library Association

SC

Supplemental content to
J Med Libr Assoc. Jul;106(3):dx.doi.org/10.5195/jmla.2018.478
www.jmla.mlanet.org
© Sollenberger 2018

SLIDE 52: EMOTIONAL CULTURE

The other is the emotional culture: the cues, values, and norms that govern which emotions people have and express at work. We're talking about things like compassion, joy, and yes, kindness. I will always remember the phrase that Catherine Deiss, the facilitator for the Association of Academic Health Sciences Libraries (AAHSL)/ National Library of Medicine (NLM) Leadership Fellows Program, used every year that I was involved.

SLIDE 53: CULTURE EATS STRATEGY FOR LUNCH

She would say, "CULTURE EATS STRATEGY FOR LUNCH." There is some controversy about who actually said this. It's a phrase attributed to Peter Drucker and made famous by one of the presidents of the Ford Motor company [34]. In any case, I like the sentiment. It's the organizational culture—and I'd like to suggest, maybe even the emotional culture—that takes priority over the strategy, plans and policies, and the processes.

SLIDE 54: TEDDY ROOSEVELT QUOTE

Teddy Roosevelt is quoted as saying, "Nobody cares how much you know until they know how much you care" [35]. I like this one. It captures so well my main theme, that caring, compassion, and kindness are the things that matter. What makes our work lives worthwhile? It's the people we work with that make us want to go in to work every day!

SLIDE 55: TOM PETERS BOOK

Tom Peters, in his book *The Little Big Things: 163 Ways to Pursue Excellence*, says that kindness is one of those little big things. Specifically, he says "Kindness is free!" [36]. In these days of budget reductions and worries about finances, that is a good thing to remember.

SLIDE 56: COUNTRY AND WORLD

I know it's risky, but how can I put together a lecture on kindness and compassion without mentioning our country's and our world's situation right now. If only—if only our leaders could show toward those who need it most the compassion and kindness that we all know is so central to success in our own lives and our own workplaces. If only…

SLIDE 57: BUDDHA QUOTE

I've talked a great deal about mindfulness and about kindness. I think I should make the connection between the two very explicit. A quote by Buddha says it very succinctly: "What we think, we become" [37]. When we are self-aware and we nurture our self-respect and self-confidence; when we listen attentively; when we focus on today rather than regretting yesterday or worrying about tomorrow; when we observe and embrace our own feelings and emotions and open ourselves up to being more sensitive and compassionate toward the people and situations around us, *then* we are being mindful of ourselves and others and we can *direct* our thoughts and emotions, rather than simply react to them.

Perhaps the most important part of all of this mindfulness stuff is what Fredrik Arnander says in his book, *We Are All Leaders* [38]. He says "that we always have complete control over two things—our interpretation of reality and our responses to it."

I'm going to tell you one more story. It happened during one of my very first jobs. I was a junior person in the organization, but I was working on some important projects because I had proven myself as a solid thinker and a hard worker. I was in a meeting with about ten other people, all of them above my paygrade—leaders, department and division heads—when the director of my department (I'm going to call her Dina the Difficult) said, in a very accusatory way, that I had neglected to do something that I was supposed to do. She really put me down. I don't even remember what that thing was; I don't remember the project. I just remember, in a very visceral way, how I felt.

JMLA Journal of the Medical Library Association

SC

Supplemental content to
J Med Libr Assoc. Jul;106(3):dx.doi.org/10.5195/jmla.2018.478
www.jmla.mlanet.org
© Sollenberger 2018

Dina was absolutely brilliant; she was important; she was animated and captivating; she was a star; she could also fly off the handle pretty easily, but everyone just accepted her volatility and let her do and say what she wanted. This was the first time that I had been the target of her wrath, and I felt about two inches tall!

SLIDE 58: MELT INTO THE GROUND

I wanted to melt into the ground and disappear. I remember replying to Dina's rebuff in a somewhat submissive manner, quietly and calmly trying to make the point that I really *had* done what she thought I should have. At least the others in the room might know that I wasn't a flunky, but she was definitely not listening, and when she left the room, I knew she still thought I was (a flunky, that is)!

I was humiliated. I was angry that she could get away with this. Unfortunately, the culture of the organization at that point in time was not one that held mindfulness or kindness in high regard. Rather, many of the people who rose to the top were definitely *very* smart, sometimes arrogant, and always focused on the work and not necessarily the people. There was one big exception to that, my immediate supervisor (I'll call her Shawn), who reported to Dina. She was my hero, the leader I wanted to be one day. She was smart, kind, and supportive. I went in and talked to Shawn; she assured me that no one in the meeting would think any less of me because of the incident. Not to worry. I wasn't convinced. I told Shawn, though, that I just must talk with Dina and tell her how I was feeling. What she had done wasn't right, and I needed her to know. Shawn encouraged me and gave me the confidence I needed to go ahead and talk with Dina.

SLIDE 59: STATUES SHOWING COMMUNICATION

I waited until most people had gone home, everyone but Dina, who always came in late and stayed late. I walked to her office door with my heart pounding and my hands shaking, but with a strong inner resolve to let Dina know how her behavior had affected me, that what she said about my work was not correct, but the way she said it had hurt me, had shaken my self-confidence and had made me feel demeaned. I tried to approach her, however, with calm, with self-respect, and with sincerity. The encounter was a positive one. I let Dina know how I felt, and I made it clear that I hoped she would not do that in the future. She listened. I saw a side of her that I hadn't seen before.

SLIDE 60: HANDSHAKE

She apologized. And she *never* did anything like that to me again. I'm not sure it changed the culture, but perhaps it changed her just a little bit. And it made a big impression on me; it was a turning point. I realized that self-respect and respect for others go hand in hand. That no matter what happens, truthfulness and integrity, along with a kind approach, can go a long way.

SLIDE 61: BRIGHT, COLORFUL WORKPLACE

All right, how does that relate to mindfulness? I think that by bringing mindfulness to work, we can change the way our bosses, our colleagues, and our customers affect us. We can change the way we respond to difficult situations. And perhaps we can change, little by little, the emotional culture of our organizations.

I want you to know that I have not become a master meditator. I do it sporadically, and I don't always feel I have been successful at calming my mind. I did it this morning before I came here, and it helped. The mindfulness that I tried to bring to the workplace was also fleeting, at best.

SLIDE 62: JOY AT WORK

But what I am sure of is that compassion, kindness, thoughtfulness, caring, and joy—these belong in our workplaces just as much, if not more, than searching skills, or strategic planning, or big-picture visions.

It really is all about the people!

Supplemental content to
J Med Libr Assoc. Jul;106(3):dx.doi.org/10.5195/jmla.2018.478
www.jmla.mlanet.org
© Sollenberger 2018

SLIDE 63: QUOTE FROM ROBERT JOYNT, MD

I'd like to share with you two quotes. The first is from Dr. Robert Joynt, former chief executive officer and dean at the University of Rochester Medical Center. He is often quoted as saying, "You can't always be right, but you can always be kind" [39].

SLIDE 64: QUOTE FROM JON KABAT-ZINN

The second is from Jon Kabat-Zinn, from his book *Arriving at Your Own Door.* He says, "In Asian languages, the word for mind and the word for heart are the same word. So when we hear the word mindfulness, we have to inwardly also hear heartfulness" [40].

I know it's getting late, but I actually have a brief mindfulness experience in store for you before you leave, but before we get to that, I want to say thank you. First, to Lucretia McClure, Barbara Epstein, AHIP, FMLA, and M.J. Tooey, former Janet Doe lecturers and friends who supported me along the way.

SLIDE 65: MLA AND AAHSL FRIENDS

Next, all of you, my colleagues in MLA, and in AAHSL—especially my two friends who unfortunately could not be here today, Jacqueline D. Doyle, FMLA, and Mary Ryan, FMLA.

SLIDE 66: MINER LIBRARY COLLEAGUES

Next, the wonderful people at the Edward G. Miner Library. Some of you are in the audience: Donna, Angela, Daniel, Linda, Liz, Helene, Jennifer. You are the people who have made me want to go to work every day!

SLIDE 676: FAMILY

Then there is this family of mine: my husband of forty-five years Dale; my sons Ian and Dane, and their wives Lori and Katie. You have been there when I needed you.

SLIDE 68: MINDFULNESS EXERCISE

Now, a very brief mindfulness experience. My son Ian (who is an actor by profession and has a much more soothing voice than I do) is going to lead you through this three-minute exercise [41].

[CHIME SOUND]

[Ian Sollenberger comes to podium:]

Hi there. Real quick. She didn't ask me to do this, but I just want to say that I went to my mom's retirement about a month and a half ago, and to see the way that that culture in her library revolved around people and compassion and kindness, and the leader that she's been, was absolutely incredible. I hope that this presentation brings some of that into your workplaces as well.

All right. Make sure you're seated, and if you would, please close your eyes. Trust me; nothing weird will happen.

I invite you, when you take your next breath, to simply be more conscious. Allow your breath to bring you into the present moment, into the here and now. Breathe in through your nose. Inhale cool air and nurture yourself. Exhale warm air, and expel any tension and any negative emotions. Breathe in through your nose and out through your mouth. In and out. Feel your feet connecting you to the floor and the earth.

Gently correct your posture by slowly lifting your chin up until the top of your head radiates toward the sky. Keep breathing. Relax your shoulders. Let them melt down like ice melting in a hot spring and feel your neck growing longer. Notice your breath, and allow it to bring you into the present moment where you are safe, relaxed, and doing something positive for yourself.

Try to quiet your mind. When thoughts do come, because they will, let them go by, like leaves floating in a mountain stream. Bring your attention back to your breath. Disengage from the past and

Journal of the Medical Library Association

Supplemental content to
J Med Libr Assoc. Jul;106(3):dx.doi.org/10.5195/jmla.2018.478
www.jmla.mlanet.org
© Sollenberger 2018

from the future. As you breathe, enjoy fullness in the present moment. In and out. Begin to cultivate a safe place in your heart, filled with love and light.

Breathe. Open your eyes. Practice kindness. Enjoy your day.

REFERENCES

1. Brainy Quotes. Ralph Waldo Emerson quotes [Internet]. [cited 29 Jan 2017]. <http://www.brainyquote.com/quotes/ralph_waldo_emerson_386697>.
2. Rees AM. Communication in the physician-patient relationship [Doe lecture, 19 May 1992]. Bull Med Libr Assoc. 1993 Jan;81(1):1–10. (Available from: <http://www.ncbi.nlm.nih.gov/pmc/articles/PMC225721/>. [cited 20 Jan 2017].)
3. Holmes OW. Valedictory address, delivered to the graduating class of the Bellevue Hospital College, March 2, 1971. NY Med J. 1871;13:436–7.
4. Peabody FW. Doctor and patient: papers on the relationship of the physician to men and institutions. New York, NY: Macmillan; 1930. p. 33.
5. Engel GL. The need for a new medical model: a challenge for biomedicine. Science. 1977 Apr 8;196(4286):129–36.
6. Engel GL. The clinical application of the biopsychosocial model. Am J Psych. 1980 May;137:5.
7. Epstein R. Attending: medicine, mindfulness, and humanity. New York, NY: Scribner; 2017.
8. Frankel RM, Quill TE, McDaniel SH, eds. The biopsychosocial approach: past, present, future. Rochester, NY: University of Rochester Press; 2003. p. 26.
9. Dannefer EF, Hundert EM, Henson LC. Medical education reform at the University of Rochester and the biopsychosocial tradition. In: Frankel RM, Quill TE, McDaniel SH, eds. The biopsychosocial approach: past, present, future. Rochester, NY: University of Rochester Press; 2003. p. 135–47.
10. Epstein, RM. Mindful practice. JAMA. 1999 Sep;282(9):833–9.
11. University of Rochester Center for Experiential Learning. Invitation: mindful practice workshop. In: CME Registration email discussion list [Internet]. Rochester, NY: The Center; 26 Jul 2016 [cited 13 Jan 2016]. [1 screen].
12. Kabat-Zinn J. Wherever you go there you are: mindfulness meditation in everyday life. New York, NY: Hachette Books; 1994. p. 4.
13. Doty JR. Into the magic shop: a neurosurgeon's true story of the life-changing magic of compassion and mindfulness. London, UK: Yellow Kite; 2016.
14. Youngson R. Time to care – how to love your patients and your job [Internet]. Raglan, New Zealand: Hearts in Healthcare; 2013 [cited 15 Feb 2017]. <http://heartsinhealthcare.com/learn/time-to-care/>.
15. Schwartz KB. A patient's story. Boston Globe Mag [Internet]. 16 Jul 1995. (Available from: <https://www.bostonglobe.com/magazine/1995/07/16/patient-story/q8ihHg8LfyinPA25Tg5JRN/story.html>. [cited 2 Feb 2017].)
16. Schwartz rounds® [Internet]. Boston, MA: The Schwartz Center; 2017 [cited 16 Feb 2017]. <http://www.theschwartzcenter.org/supporting-caregivers/schwartz-center-rounds/>.
17. Gregoire C. Actually TIME, this is what the 'mindful revolution' really looks like. Huffington Post [Internet]. 2014 Feb 4 [cited 20 Feb 2017]. <http://www.huffingtonpost.com/2014/02/04/this-is-proof-that-mindfu_n_4697734.html>.
18. Davidson RJ, Begley S. The emotional life of your brain: how its unique patterns affect the way you think, feel, and live—and how you can change them. New York, NY: Plume; 2013.
19. Siegel DJ. The mindful brain: reflection and attunement in the cultivation of well-being. New York, NY: W.W. Norton; 2007.
20. Hamilton DR. Why kindness is good for you. Carlsbad CA: Hay House; 2010.
21. Loizzo J. Meditation research, past, present, and future: perspectives from the Nalanda contemplative science tradition. Ann NY Acad Sci. 2014 Jan;1307(1):43–54.
22. Black DS, Slavich GM. Mindfulness meditation and the immune system: a systematic review of randomized controlled trials. Ann NY Acad Sci. 2016 Jun;1373(1):13–24.
23. Gentry WA, Weber TJ, Sadri G. Empathy in the workplace: a tool for effective leadership [white paper based on a poster presented at the Society of Industrial Organizational Psychology Conference; NY; Apr 2007] [Internet]. Greensboro, NC: Center for Creative Leadership; 2016 [cited 3 Feb 2017]. <https://www.ccl.org/wp-content/uploads/2015/04/EmpathyInTheWorkplace.pdf>.
24. Center for Creative Leadership. Mindfulness: a simple way to lead better—an interview with Janice Marturano, author of Finding the Space to Lead [Internet]. Greensboro, NC: The Center; 2016 [cited 3 Feb 2017]. <http://www.ccl.org/articles/leading-effectively-articles/mindfulness-a-simple-way-to-lead-better/>.
25. Gardiner B. Business skills and Buddhist mindfulness. Wall Street J. 2012 Apr 3. (Available from: <http://www.wsj.com/articles/SB10001424052702303816504577305820565167202/>. [cited 10 Feb 2017].)

Supplemental content to
J Med Libr Assoc. Jul;106(3):dx.doi.org/10.5195/jmla.2018.478
www.jmla.mlanet.org
© Sollenberger 2018

26. Schaufenbuel K. Why Google, Target, and General Mills are investing in mindfulness. Harvard Bus Rev. 28 Dec 2015. (Available from: <http://hbr.org/2015/12/why-google-target-and-general-mills-are-investing-in-mindfulness/>. [cited 11 Feb 2017].)
27. Kelly C. O.K., Google, take a deep breath. NY Times [Internet]. 28 Apr 2012. (Available from: <http://www.nytimes.com/2012/04/29/technology/google-course-asks-employees-to-take-a-deep-breath.html>. [cited 12 Feb 2017].)
28. Search Inside Yourself Leadership Institute [Internet]. San Francisco, CA: The Institute; 2017 [cited 2 Mar 2017]. <http://siyli.org>.
29. Tan CM. Search inside yourself: the unexpected path to achieving success, happiness (and world peace). New York, NY: Harper One; 2012.
30. Goleman D. Emotional intelligence: why it can matter more than IQ. New York, NY: Bantam Books; 2005.
31. Goleman D. Social intelligence: the new science of human intelligence. New York, NY: Bantam Books; 2006.
32. Goleman D, Boyatzis R. Social intelligence and the biology of leadership. Harvard Bus Rev. Sep 2008. (Available from: <http://hbr.org/2008/09/social-intelligence-and-the-biology-of-leadership/>. [cited 20 Feb 2017].)
33. Barsade S, O'Neill OA. Mange your emotional culture. Harvard Bus Rev. Jan–Feb 2016. (Available from: <http://hbr.org/2016/01/manage-your-emotional-culture/>. [cited 20 Feb 2017].)
34. Quote Investigator. Culture eats strategy for breakfast [Internet]. [cited 4 Mar 2017]. <http://quoteinvestigator.com/2017/05/23/culture-eats/>.
35. Brainy Quotes. Theodore Roosevelt quotes [Internet]. [cited 4 Mar 2017]. <http://www.brainyquote.com/citation/quotes/theodore_roosevelt_140484/>.
36. Peters T. The little big things: 163 ways to pursue excellence. New York, NY: Harper Business; 2010. p. 77–8.
37. Brainy Quotes. Buddha quotes [Internet]. [cited 4 Mar 2017]. <http://www.brainyquote.com/authors/buddha/>.
38. Arnander F. We are all leaders: leadership is not a position—it's a mindset. West Sussex, UK: Capstone; 2013.
39. Shoulson I. In memoriam: Robert J. Joynt, MD, PhD (1925–2012). Arch Neur. 2012;69(11):1518–9.
40. Kabat-Zinn J. Arriving at your own door: 108 lessons in mindfulness. New York, NY: Hachette Books; 2007.
41. Weinblatt V. 3-minute guided meditation script for any occasion [Internet]. The Genius of Yoga [cited 12 Mar 2017]. <https://thegeniusofyoga.com/3-minute-guided-meditation-script/>.

SLIDE IMAGE CREDITS

1. Child blue eye looking at camera close up. Image 187014512. Shutterstock. Standard License.
2. Sollenberger J, photographer. Monkey on my monitor.
3. Lincoln A, photographer. 9 Jun 2010. Photo used with permission of Lucretia McClure.
4. Mabel J, photographer. 3 Aug 2014. Mount Rainier National Park, WA. View in the righthand rear view mirror approaching Longmire, driving east on US Route 12. Wikimedia Commons: Creative Commons Attribution-Share Alike 3.0 License.
5. Dragan A, photographer. 23 Nov 2015. Future world.
6. Close-up of eyes. Image 27113176. Adobe Stock Images. Standard License.
7. Sun T, photographer. Image 594502556. Shutterstock. Standard License.
8. Upper left: Grandma's best sugar cookies. http://showmetheyummy.com/new-years-eve-recipes-2015/. Permission granted by blog owner.
   Upper right: Good 'N Plenty. Shoofly pie, Lancaster County. 31 May 2016. https://commons.wikimedia.org/wiki/File:Shoofly-pie-lancaster-county.jpg. Wikimedia commons. Creative Commons Attribution-Share Alike 4.0 International.
   Middle: MachineKeebler, photographer. 1 May 2009. Whoopie pies. http://commons.wikimedia.org/wiki/File:Whoopiepies1.jpg. Wikimedia Commons. Creative Commons Attribution-Share Alike 3.0.
   Lower right: Elliott M, photographer. 28 Apr 2010. Sticky buns-2. Flickr. https://www.flickr.com/photos/10181902@N00/4561125084. Creative Commons Attribution No-Derivs 2.0 Generic.
   Lower left: Apple cider fritters. Sugarhero.com. http://www.sugarhero.com/apple-cider-fritters/.
9. Upper left: Eytan T, photographer. Mennonites conducting an event in Farragut Square, Washington, DC. 8 Oct 2011; image 5934. Flickr. Creative Commons Share-Alike 2.0 Generic.
   Upper right: Pxhere; image 1003201. Creative Commons CCO.
   Lower left: Reese DB, photographer. http://reesephoto.me/all-galleries-3/amish/#jp-carousel-253/. Permission granted by the photographer.
   Lower middle: Collins M, photographer. Nov 1942. Lititz, PA. Old Mennonite. Library of Congress, Prints & Photographs Division, FSA/OWI Collection, [LC-DIG-fsa-8d23489]. Public domain.
   Lower right: Reese DB, photographer. http://reesephoto.me/all-galleries-3/amish/#jp-carousel-254/. Permission granted by the photographer.

Supplemental content to
J Med Libr Assoc. Jul;106(3):dx.doi.org/10.5195/jmla.2018.478
www.jmla.mlanet.org
© Sollenberger 2018

10. Schaefer E, designer. Pixabay. http://pixabay.com/en/info-icon-button-web-help-blue-1459077/. Creative Commons CCO.
11. Consumer health information source book, Alan M. Rees, ed. Book cover. Amazon. https://www.amazon.ca/Consumer-Health-Information-Source-Book/dp/1573565091/.
12. Anderson H, illustrator. Permission granted by Museum of Illustration and Comic and Cartoon Art at the Society of Illustrators, New York, NY.
13. William McCann at bedside, Strong Memorial Hospital, with group of residents. 1950. Permission granted by the Photographic Archive, Rare Books & Manuscripts Section, Edward G. Miner Library, University of Rochester Medical Center.
14. No attribution required.
15. Stefancik S, photographer. Man in gray shirt portrait. http://www.pexels.com/photo/man-young-happy-smiling-91227/. Creative Commons CCO.
16. Left: George Engel. Permission granted by Photographic Archive, Rare Books & Manuscripts Section, Edward G. Miner Library. University of Rochester Medical Center.
    Right: James Palis, photographer, mid-1970s. John Romano. Permission granted by the Photographic Archive, Rare Books & Manuscripts Section, Edward G. Miner Library, University of Rochester Medical Center.
17. George Engel. 1987. Photo from the Papers of George Libman Engel, MD Permission granted by the Rare Books & Manuscripts Section, Edward G. Miner Library, University of Rochester Medical Center.
18. Raths A, photographer. Female senior patient tells the doctor about her health complaints. Image 456100060. Shutterstock. Standard license.
19. Poggemann KL, photographer. 2 Mar 2017. DNA-double-helix through an electron microscope. http://www.flickr.com/photos/hinkelstone/33255693331/in/photostream/. Creative Commons Attribution 2.0 Generic.
20. Reardon SS, photographer. Ronald Epstein, MD, website. http://www.ronaldepstein.com.
21. Attending, by Ronald M. Epstein. Book cover. Amazon. http://www.amazon.com/Attending-Dr-Ronald-Epstein-M-D/dp/1501121715/.
22. No attribution required.
23. Leverich R, rendering artist. Chapin Mill Retreat Center. Permission granted by Bero Architecture, designers.
24. Retreat Center dinner. Permission granted by Rochester Zen Center.
25. Retreat Center Zen room. Permission granted by Rochester Zen Center.
26. Sollenberger J, photographer. Autumn day at the Chapin Mill Retreat Center.
27. Stress reduction, Jon Kabat-Zinn. MP3 album cover. Amazon. http://www.amazon.com/gp/product/B00BADZNU8/ref=dm_ws_sp_ps_dp/.
28. Carlsen B, photographer. Mindfulness retreat moment. Website of Ronald Epstein, MD. http://www.ronaldepstein.com.
29. Tears in eyes. http://commons.wikimedia.org/wiki/File:USMC-04952.jpg#metadata. Wikimedia Commons. Public domain (image taken by US Department of Defense employee).
30. No attribution required.
31. Schwartz Rounds. Permission granted by the Schwartz Center.
32. No attribution required.
33. No image.
34. Seung Labs, photographers. 31 Jul 2014. Eyewire. Neurons—Dense. Flickr. http://www.flickr.com/photos/123689703@N04/14608708208/in/album-72157646065956112/. Creative Commons Non-Commercial Share-Alike 2.0 Generic.
35. Left: The emotional life of your brain, Richard J. Davidson and Sharon Begley. Book cover. Richard J. Davidson website. http://www.richardjdavidson.com/the-emotional-life-of-your-brain/.
    Right: Richard J. Davidson website. https://centerhealthyminds.org/about/founder-richard-davidson.
36. Left: The mindful brain, Daniel J. Siegel. Book cover. Daniel J. Siegel website. http://www.drdansiegel.com/images/MindfulBrain_LG.jpg.
    Right: Why kindness is good for you, David R. Hamilton. Book cover. Amazon. https://www.amazon.com/Why-Kindness-Good-David-Hamilton/dp/1848501781.
37. Panisanun, creator. Dopamine: optimized geometry. Wikimedia Commons. http://commons.wikimedia.org/wiki/File:Dopamine_finalver.png. Creative Commons Attribution-Share Alike 4.0 International.
38. Left: Benjah-bmm27, creator. Cortisol. Wikimedia Commons. http://commons.wikimedia.org/wiki/File:Cortisol-3D-vdW.png. Public domain.
    Right: Blood pressure measurement. Wikimedia Commons. https://commons.wikimedia.org/wiki/Category:Blood_pressure_measurement. Creative Commons Attribution-ShareAlike 3.0 Unported.
39. No attribution required.

Supplemental content to
J Med Libr Assoc. Jul;106(3):dx.doi.org/10.5195/jmla.2018.478
www.jmla.mlanet.org
© Sollenberger 2018

40. Marcellno JG, creator. Connectome. Wikimedia Commons. http://commons.wikimedia.org/wiki/File:Connectome.jpg. Creative Commons Attribution 2.0 Generic.
41. Hetrick Z, photographer. Oboe player. Wikimedia Commons. http://commons.wikimedia.org/wiki/File:Oboe-player.jpg. Creative Commons Attribution-Share Alike 2.0 Generic.
42. National Institute of Mental Health, National Institutes of Health. Multi-color image of whole brain for brain imaging research. 5 Jan 2010. http://images.nimh.nih.gov/public_il/image_details.cfm?id=407. Public domain.
43. Kaufman L, source. National Cancer Institute. 1980. Brain MRI. http://visualsonline.cancer.gov/details.cfm?imageid=2416. Public domain.
44. Bru-nO. Pixabay. http://pixabay.com/en/defense-protection-threat-1403072/. Creative Commons CCO.
45. Students in Miner Library. Permission granted by the Rare Books & Manuscripts Section, Edward G. Miner Library, University of Rochester Medical Center.
46. Kind word on red keyboard button. Image 529267879. Shutterstock. Standard license.
47. Logos used with permission of Center for Creative Leadership, the Mind & Life Institute, and the Institute for Mindful Leadership.
48. No attribution required.
49. Left: Search inside yourself, Chade-Meng Tan. Book cover. Amazon. http://www.amazon.com/Search-Inside-Yourself-Unexpected-Achieving/dp/0062116932.
Center: Emotional intelligence, Daniel Goleman. Book cover. Amazon. http://www.amazon.com/Emotional-Intelligence-10th-Anniversary-Matter/dp/055380491X/.
Right: Social intelligence. Daniel Goleman. Book cover. Amazon. http://www.amazon.com/Emotional-Intelligence-Matter-More-Than/dp/055338371X/.
50. Neural pathways artistically think. Pixabay. http://pixabay.com/en/neural-pathways-artistically-think-221719/. Creative Commons CCO.
51. Vladgrin, creator. Image 385748146. Shutterstock. Standard License.
52. Alexas_Fotos, creator. Pixabay. http://pixabay.com/en/meeting-lecture-group-team-2044759/. Creative Commons CCO.
53. jjfbbennett, creator. 9 Dec 2012. Culture eats strategy. Flickr. http://www.flickr.com/photos/jjfbbennett/8256143475/. Creative Commons Attribution-NonCommercial-ShareAlike 2.0 Generic.
54. Theodore Roosevelt. Copyright 1915. Wikimedia commons. http://en.wikipedia.org/wiki/File:T_Roosevelt.jpg. Public domain.
55. The little big things, by Tom Peters. Book cover. Amazon. http://www.amazon.com/Little-Big-Things-Pursue-EXCELLENCE/dp/0061894087/.
56. Left: GDJ—http://openclipart.org/user-detail/GDJ, creator. Various variants of world globe. 30 May 2015. Wikimedia Commons. http://commons.wikimedia.org/wiki/File:GDJ-World-Flags-Globe.svg. Public domain.
Right: El bart089, creator. Taken from Alex Covarrubias' map of North America. Wikimedia Commons. http://commons.wikimedia.org/wiki/File:North_America_map_coloured.svg#/media/File:North_America_map_coloured.svg. GNU Free Documentation License.
57. Geralt, creator. Pixabay. http://pixabay.com/en/yoga-buddha-deity-shiva-water-386611/. Creative Commons CCO.
58. Solberg AA, photographer. 20 Mar 2007. Snowman melting. Flickr. http://www.flickr.com/photos/andreassolberg/428262012/. Creative Commons Attribution-NonCommercial-ShareAlike 2.0 Generic.
59. Couleur, photographer. Pixabay. http://pixabay.com/en/sculpture-bronze-2196139/. Creative Commons CCO.
60. Johnhain, creator. Pixabay. http://pixabay.com/en/handshake-regard-cooperate-connect-2009195/. Creative Commons CCO.
61. Geralt, creator. Pixabay. http://pixabay.com/en/man-businessmen-woman-economy-1470839/. Creative Commons CCO.
62. Andersen S, photographer. April 2017. Edward G. Miner librarians.
63. No image.
64. Puckillustrations, creator. Image 25842161. Adobe Stock Images. Standard License.
65. No attribution required.
66. Andersen S, photographer. Edward G. Miner Library staff.
67. Upper Left: Photo by Erica Camille Photography.
Upper Right: Photo by Logan Rose Photography.
68. ForestWander, photographer. Source: http://www.forestwander.com/2009/02/fall-leaves-floating/. Wikimedia Commons. http://commons.wikimedia.org/wiki/File:Fall-leaves-floating_-_West_Virginia_-_ForestWander.jpg. Creative Commons CCO.
